# Discovery of Polyoxypregnane Derivatives From *Aspidopterys obcordata* With Their Potential Antitumor Activity

**DOI:** 10.3389/fchem.2021.799911

**Published:** 2022-01-05

**Authors:** Hong-Wei Guo, Yun-Gang Tian, Yi-Han Liu, Jia Huang, Jian-Xia Wang, Hua Long, Hua Wei

**Affiliations:** ^1^ College of Biology and Environmental Science, Jishou University, Jishou, China; ^2^ School of Medicine, Jishou University, Jishou, China; ^3^ School of Pharmaceutical Sciences, Jishou University, Jishou, China; ^4^ Tujia Medicine Research Center in Hunan (Jishou University), Jishou, China

**Keywords:** *Aspidopterys obcordata*, ethnic medicine, polyoxypregnane derivatives, HL-60, structure–activity relationship

## Abstract

The bioassay-guided phytochemical study of an ethnic medicinal plant *Aspidopterys obcorda* ta Hemsl. var. obcordata results in the isolation of eight new polyoxypregnane derivatives, named aspidatasides A–H (1–8), along with ten known analogs (9–18). The series polyoxypregnane derivatives were screened for their cytoxic activity against HL-60 cells, and compound 2 showed the highest potency with an IC_50_ 8.03 μM. Preliminary structure–activity relationship studies displayed that the sugar chain and double bond could notably impact their biological activity.

## 1 Introduction

Natural products, as major chemical resources, make up significant agents in modern drug discovery, and play an important role in treating and preventing diseases with novel molecular mechanisms over the last 200 years (Hanson, 2017). The latest report shows that half of the new drugs in the market come from natural products or structural modifications based on a natural chemical framework (Newman and Cragg, 2020).

The polyoxypregnanes and their derivatives as valuable therapeutic agents are naturally occurring C_21_ steroidal skeleton or substituted by oligosaccharide chain, benzoyl, acylated tigloyl ester groups, as well as an extra epoxide ring (Bai et al., 2007; Zheng et al., 2014), which exhibited diverse biological and medicinal activities, such as anti-inflammatory effect, immune-suppressive effect; chondro-protective effect; and antifungal, antioxidant, antifertility, anti-AchE, and anti-HIV activities (Abe et al., 2000; Niranjan et al., 2002; Plaza et al., 2004; Deng et al., 2005; Li et al., 2007; Sanyacharernkul et al., 2009; Ni and Ye, 2010; Wang et al., 2010; Itthiarbha et al., 2012; Pang et al., 2015; Zhang et al., 2015; Gu and Hao, 2016; Wang et al., 2016; Pang et al., 2017; Zhan et al., 2019). In particular, many of them have shown prominent anticarcinogenic or cancer inhibitory activities with great research and development potential, and received widespread attention by pharmacologists (Plaza et al., 2005; Panda et al., 2006; Bai et al., 2009; Shen and Zhang, 2010; Zhang et al., 2010; Xue et al., 2012; Sun et al., 2014; Yao et al., 2014; Zhang et al., 2015; Liu et al., 2018; Song et al., 2018). Natural monomeric polyoxypregnanes have been recognized as characteristic constituents in Asclepiadaceae plants, and there have been effective reports on the use of *Marsdenia tenacissima* (Asclepiadaceae) as a raw material in the treatment of leukemia, gastric carcinoma, liver cancer, prostate cancer, and lung cancer in China, notably (Zhao et al., 2007; Tong et al., 2015; Ge et al., 2019; Wang et al., 2019; Yu, 2019; Pu et al., 2020; Zhu et al., 2020). However, polyoxypregnanes are rarely isolated from the other families. Meanwhile, their antitumor mechanism and the structure–activity relationship of polyoxypregnane derivatives from the plant are not clearly demonstrated.

**FIGURE 1:**
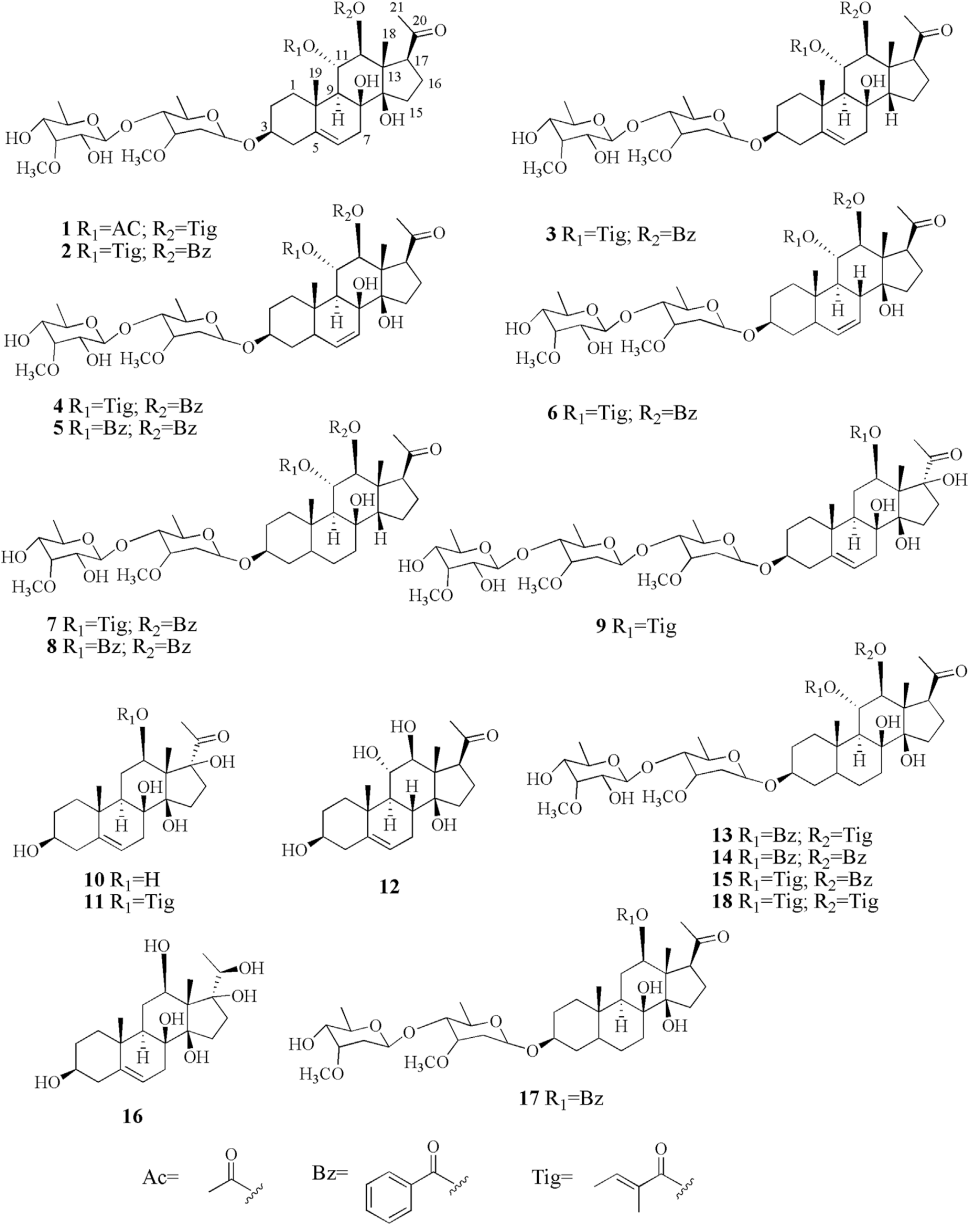
Chemical structures of compounds 1–18.

*A. obcordata* is a woody liana herb, mainly distributed in sunny and rainfall areas in Xishuangbanna, Yunnan Province. As a “Dai Medicine,” *A. obcordata* has been used frequently and commonly in the Dai Minority Hospital of Xishuangbanna, Southwest China, for the treatment of various diseases, such as cystitis, chronic nephritis, urinary tract infections, rheumatic bone pain, and postpartum body deficiency (Chen and Funston, 1997; Li et al., 2011). Although the effectiveness of the medicinal plant has been verified in clinic, there are few related studies about its material basis (Li et al., 2016; Li et al., 2019). In a continuous attempt to search for structurally diverse cytotoxicity, interesting C_21_ steroids from *A. obcordata*, eight new polyoxypregnane derivatives, named aspidatasides A–H (1–8), together with ten known analogs (9–18) (Figure 1) were obtained from the 95% EtOH extract of plant’s vines, which exhibited moderate antitumor effects, especially its antihuman myeloid leukemia (HL-60) activity. Herein, the isolated procedures, structural elucidation of the new compounds, as well as their inhibitory activity evaluation against HL-60 were reported in order to understand the structure–activity relationship (SAR).

## 2 Results and Discussion

### 2.1 Isolation and Structure Elucidation

Compound 1 was isolated as a white amorphous powder. Its IR spectrum exhibited absorptions for the groups of hydroxyl (3,382 cm^−1^) and carbonyl (1,717 cm^−1^). The molecular formula was established as C_42_H_64_O_15_ based on the molecular ion peak at *m/z* [M + Na]^+^ 831.4139 in the HR–ESI–MS (calculated for 831.4143 C_42_H_64_O_15_Na). In the ^1^H NMR spectrum (Table 1), signals for three angular methyl protons at δ_H_ 1.29 (3H, s, CH_3_-18), 1.32 (3H, s, CH_3_-19), and 2.16 (3H, s, CH_3_-21), and one olefinic proton signal at δ_H_ 5.36 (H-6) in conjunction with three carbons at δ_C_ 216.5, 140.6, and 119.5 (Table 2) suggested the basic skeleton of pregen-5-en-20-one (Wang et al., 2010; Zhang et al., 2015; Wang et al., 2019). The proton signals at δ_H_ 1.81 (3H, s, 11-OAc) and 6.73 (1H, q, *J* = 7.2 Hz, Tig-H-3), 1.57 (1H, d, *J* = 7.2 Hz, Tig-H-4), and 1.43 (3H, s, Tig-H-5), together with carbon resonances at δ_C_ 21.0, 172.9 (11-OAc), and δ_C_ 168.4, 129.9, 140.7, 14.8, and 12.3 (Tig-C), suggested the presence of acetoxyl function and tigloyl unit in the molecular structure. Two anomeric signals at δ_H_ 4.65 (1H, d, *J* = 9.6 Hz) and 4.72 (1H, d, *J* = 7.8 Hz) indicated the existence of two sugar moieties in compound 1. Two methoxyls at δ_H_ 3.39 (s) and 3.60 (s) were attributed to the sugar moiety. The IR and ^1^H and ^13^C-NMR spectra data identified that compound 1 is a polyoxypregnane glycoside derivative.

**TABLE 1:** ^1^H NMR spectroscopic data (600 MHz, 1 in MeOD and 2–8 in DMSO) for compounds 1–8 (δ_H_ in ppm, *J* in Hz).

Position	1	2	3	4	5	6	7	8
1	1.19, m	1.22, m	1.18, m	1.26, m	1.20, m	1.21, m	1.20, m	1.22, m
	1.83, m	1.81, m	1.85, m	1.82, m	1.83, m	1.83, m	1.88, m	1.90, m
2	1.95, m	1.72, m	1.69, m	1.72, m	1.74, m	1.73, m	1.71, m	1.67, m
	1.72, m	1.91, m	1.97, m	1.97, m	1.99, m	1.98, m	1.91, m	1.98, m
3	3.69, m	3.61, m	3.47, m	3.74, m	3.60, m	3.71, m	3.47, m	3.59, m
4	2.27, m	2.21, m	2.27, m	2.27, m	2.21, m	2.27, m	2.12, m	2.17, m
	2.41, m	2.40, m	2.38, m	2.38, m	2.49, m	2.41, m	1.44, m	1.44, m
5	—	—	—	3.40, m	3.40, m	3.40, m	1.17, m	1.18, m
6	5.36, d (5.4)	5.27, d (4.2)	5.46, d (2.4)	—	6.20, dd (16.2)	5.46, dd (16.2)	1.04, m	1.04, m
	—	—	—	6.50, dd (10.2)	—	—	1.57, m	1.57, m
7	1.89, m	1.86, m	1.87, m	6.6, dd (10.2)	6.28, dd (16.2)	6.52, dd (16.2)	1.37, m	1.37, m
	2.27, m	2.25, m	2.29, m	—	—	—	1.66, m	1.67, m
8	—	—	—	—	—	1.92, m	—	—
9	1.95, d (10.8)	1.96, d (10.2)	2.29, d (10.2)	1.66, d (10.8)	1.83, d (10.8)	1.76, m	2.00, d (10.2)	2.17, d (10.2)
10	—	—	—	—	—	—	—	—
11	5.81, t (10.8)	5.70, t (10.8)	5.53, t (10.2)	5.80, t (10.8)	5.98, t (10.8)	5.28, t (10.2)	5.42, t (10.2)	5.61, t (10.2)
12	5.15, d (10.8)	4.89, d (10.8)	5.23, d (10.2)	4.92, d (10.8)	5.04, d (10.8)	5.44, d (10.2)	5.18, d (10.2)	5.17, d (10.2)
13	—	—	—	—	—	—	—	—
14	—	—	1.64, m	—	—	—	1.64, m	1.64, m
15	1.51, m	1.52, m	1.48, m	1.60, m	1.52, m	1.54, m	1.47, m	1.47, m
	1.53, m	1.58, m	1.37, m	1.54, m	1.61, m	1.56, m	1.53, m	1.53, m
16	2.20, m	1.77, m	1.81, m	1.78, m	1.75, m	1.77, m	1.71, m	1.71, m
	1.78, m	2.12, m	2.38, m	2.38, m	2.12, m	2.14, m	2.11, m	2.17, m
17	3.17, m	3.17, m	3.16, m	3.16, m	3.15, m	3.15, m	3.16, m	3.14, m
18	1.29, s	1.27, s	1.18, s	1.26, s	1.23, s	1.25, s	1.17, s	1.12, s
19	1.32, s	1.31, s	1.25, s	1.29, s	1.31, s	1.27, s	1.39, s	1.37, s
20	—	—	—	—	—	—	—	—
21	2.16, s	2.11, s	1.91, s	2.06, s	2.12, s	2.06, s	2.07, s	2.06, s
11-O	Ac	Tig	Tig	Tig	Bz	Tig	Tig	Bz
2	1.81, s	—	—	—	—	—	—	—
3	—	6.70, q (7.2)	6.52, q (7.2)	6.60, q (5.4)	7.81, dd (7.2.1.2)	6.59, q (5.4)	6.50, q (7.2)	7.70, qq (7.2.1.2)
4	—	1.70, d (7.2)	1.46, d (7.2)	1.55, d (5.4)	7.78, t (7.2)	1.57, d (5.4)	1.45, d (7.2)	7.34, t (7.2)
5	—	1.48, s	1.48, s	1.60, s	7.50, t (7.2)	1.45, s	1.37, s	7.50, t (7.2)
6	—	—	—	—	7.78, t (7.2)	—	—	7.34, t (7.2)
7	—	—	—	—	7.81, dd (7.2.1.2)	—	—	7.70, qq (7.2.1.2)
12-O	Tig	Bz	Bz	Bz	Bz	Bz	Bz	Bz
3	6.73, q (7.2)	7.80, dd (7.2.1.2)	7.81, dd (7.2.1.2)	7.75, dd (7.8.2.4)	7.49, dd (7.2.1.2)	7.43, dd (7.8.2.4)	7.74, dd (7.2.1.2)	7.65, dd (7.2.1.2)
4	1.57, d (7.2)	7.43, t (7.2)	7.49, t (7.2)	7.43, t (7.8)	7.47, t (7.2)	7.36, t (7.8)	7.48, t (7.2)	7.31, t (7.2)
5	1.43, s	7.58, t (7.2)	7.63, t (7.2)	7.43, t (7.8)	7.35, t (7.2)	7.57, t (7.8)	7.63, t (7.2)	7.63, t (7.2)
6	—	7.43, t (7.2)	7.49, t (7.2)	7.43, t (7.8)	7.47, t (7.2)	7.36, t (7.8)	7.48, t (7.2)	7.31, t (7.2)
7	—	7.80, dd (7.2.1.2)	7.81, dd (7.2.1.2)	7.75, dd (7.8.2.4)	7.49, dd (7.2.1.2)	7.43, dd (7.8.2.4)	7.74, dd (7.2.1.2)	7.65 dd (7.2.1.2)
Ole-1	4.65, d (9.6)	4.59, d (9.0)	4.62, d (9.6)	4.92, d (10.2)	4.55, d (8.4)	4.92, d (10.2)	4.60, d (9.6)	4.57, d (9.6)
2	1.93, m	1.91, m	1.91, m	1.15, m	1.99, m	1.98, m	1.91, m	1.93, m
	2.24, m	2.21, m	2.23, m	2.06, m	2.21, m	2.21, m	2.25, m	2.21, m
3	3.51, m	3.52, m	3.51, m	3.26, m	3.46, m	3.46, m	3.34, m	3.47, m
4	3.19, m	3.16, m	3.16, m	3.02, m	3.18, m	3.15, m	3.27, m	3.21, m
5	3.36, m	3.35, m	3.35, m	3.14, m	3.35, m	3.34, m	3.35, m	3.34, m
6	1.23, d (6.6)	1.09, d (6.0)	1.24, d (6.0)	1.24, d (6.0)	1.14, d (6.6)	1.23, d (6.0)	1.09, d (6.6)	1.08, d (6.0)
3-OCH3	3.39, m	3.39, s	3.45, s	3.26, m	3.35, s	3.37, s	3.45, s	3.44, s
Allo-1	4.72, d (7.8)	4.56, d (8.4)	4.62, d (8.4)	4.75, d (7.2)	4.74, d (10.8)	4.55, d (7.2)	4.62, d (8.4)	4.74, d (7.2)
2	3.48, m	3.47, m	3.47, m	3.47, m	3.44, m	3.47, m	3.47, m	3.47, m
3	3.66, m	3.62, m	3.80, m	3.45, m	3.61, m	3.61, m	3.61, m	3.61, m
4	3.39, m	3.37, m	3.35, m	3.04, m	3.34, m	3.27, m	3.34, m	3.34, m
5	3.68, m	3.62, m	3.53, m	3.19, m	3.66, m	3.71, m	3.66, m	3.60, m
6	1.32, d (6.6)	1.24, d (6.0)	1.46, d (6.0)	1.09, d (6.0)	1.20, d (6.6)	1.20, d (6.0)	1.24, d (6.6)	1.20, d (6.0)
3-OCH3	3.60, s	3.50, s	3.80, s	3.45, m	3.35, s	3.90, s	3.51, s	3.51, s

**TABLE 2:** ^13^C NMR spectroscopic data (150 MHz, 1 in MeOD, and 2–8 in DMSO) for compounds 1–8.

Position	1	2	3	4	5	6	7	8
1	40.3	40.5	40.6	40.3	39.9	40.5	40.5	40.5
2	30.5	29.0	31.5	29.3	28.9	29.3	31.2	28.8
3	79.3	76.7	74.1	79.0	73.0	73.0	74.4	74.4
4	41.3	41.2	41.2	41.3	41.0	41.3	36.6	36.5
5	140.6	138.3	140.1	55.2	54.4	52.2	56.2	58.7
6	119.5	118.3	119.3	131.1	133.2	145.1	26.3	26.3
7	37.6	38.0	38.6	129.4	130.5	117.3	36.6	34.3
8	76.8	75.0	70.1	77.8	77.4	30.8	65.9	71.2
9	50.0	47.6	56.3	50.2	49.8	48.9	50.5	50.5
10	39.9	40.0	40.1	40.0	39.2	40.0	38.4	38.5
11	72.7	70.6	70.5	73.5	73.0	70.8	68.1	70.5
12	79.4	77.6	78.5	79.0	75.1	75.1	74.9	78.5
13	56.2	54.5	45.4	55.1	54.8	53.9	45.3	45.4
14	86.5	84.5	48.0	84.9	84.4	82.8	42.7	42.7
15	36.2	36.5	36.5	35.2	36.5	36.6	34.3	36.8
16	25.1	29.0	26.4	24.1	24.3	23.0	28.8	26.4
17	60.1	54.5	56.3	59.4	61.3	61.3	58.6	56.2
18	13.5	11.6	11.4	13.0	12.6	11.5	11.4	12.5
19	18.4	18.4	18.5	18.4	13.4	18.4	18.3	18.3
20	216.5	211.6	210.1	212.0	211.5	211.1	210.1	210.1
21	31.0	30.6	30.2	31.2	30.8	30.8	30.1	31.2
11-O	Ac	Tig	Tig	Tig	Bz	Tig	Tig	Bz
1	172.9	166.9	166.2	166.7	166.1	166.1	166.4	165.4
2	21.0	127.5	127.6	128.4	133.6	127.9	127.7	129.3
3	—	138.0	138.3	138.5	128.8	138.1	137.9	128.9
4	—	14.2	16.1	14.6	128.5	11.8	13.9	128.5
5	—	12.8	14.0	12.1	130.5	11.7	12.4	133.3
6	—	—	—	—	128.5	—	—	128.5
7	—	—	—	—	128.8	—	—	128.9
12-O	Tig	Bz	Bz	Bz	Bz	Bz	Bz	Bz
1	168.4	165.9	165.1	166.5	164.9	166.4	165.1	165.1
2	129.9	128.0	128.9	134.3	129.6	133.8	128.9	128.5
3	140.7	129.2	129.1	129.4	128.2	128.5	129.0	128.8
4	14.8	129.0	128.5	128.8	129.1	128.4	128.5	128.4
5	12.3	129.3	133.5	131.1	133.2	130.7	133.4	133.2
6	—	129.0	128.5	128.8	129.1	128.4	128.5	128.4
7	—	129.2	129.1	129.4	128.2	128.5	129.0	128.8
Ole-1	98.9	96.7	96.6	96.7	96.2	96.3	96.3	96.3
2	38.0	38.7	37.9	37.7	37.2	37.0	34.3	38.2
3	80.7	78.6	75.9	79.1	78.5	78.6	78.5	74.9
4	84.2	82.8	82.8	83.2	82.8	82.3	82.7	82.8
5	72.4	70.5	69.3	72.0	70.5	70.5	70.5	69.3
6	19.0	18.0	18.3	18.8	18.3	17.9	17.9	17.9
3-OCH3	57.6	56.3	58.8	56.7	56.2	56.3	56.2	56.2
Allo-1	102.4	100.8	100.8	101.2	100.7	100.8	100.7	100.7
2	73.8	71.6	71.6	73.5	71.1	70.8	71.1	71.5
3	84.1	82.3	82.2	82.7	82.2	82.2	82.2	82.2
4	75.2	73.0	73.0	75.6	71.5	71.6	73.0	73.0
5	71.4	69.4	68.3	71.0	69.3	69.3	69.3	68.8
6	18.4	17.2	17.9	18.4	17.9	14.1	16.3	16.4
3-OCH3	62.7	61.4	61.3	61.8	61.3	61.3	61.3	61.3

**FIGURE 2:**
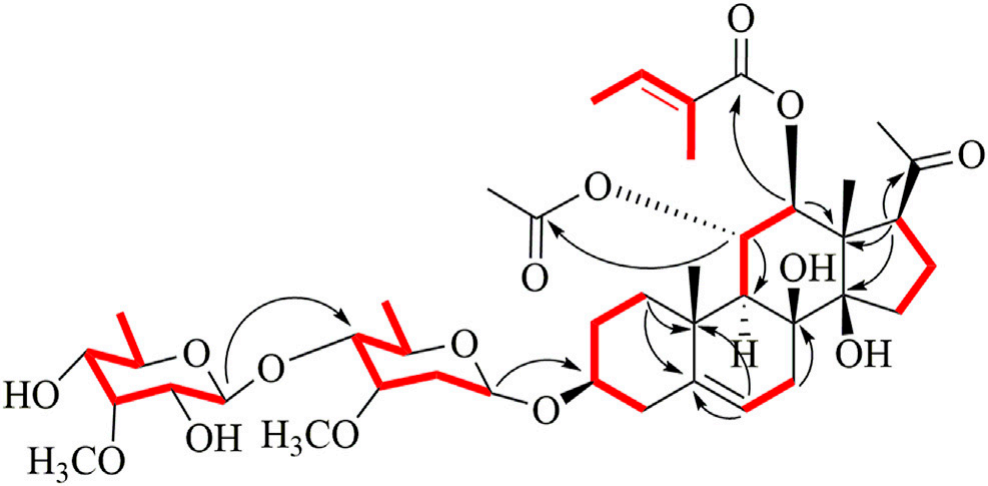
Key HMBC and ^1^H-^1^H COSY correlations of compound 1.

All assignments of proton signals were assigned by its HSQC spectrum (Supporting Information, Supplementary Figure S3). The connectivities of compound 1 were deduced mainly by ^1^H-^1^H COSY and HMBC spectra (Figure 2). The correlations among H-1/H-2/H-3/H-4, H-6/H-7, H-9/H-11/H-12, and H-15/H-16/H-17 were found in the ^1^H-^1^H COSY spectrum. The position of acetoxyl and tigloyl groups was determined at C-11 and C-12, respectively, as the HMBC correlations between δ_H_5.81 (1H, t, *J* = 10.8 Hz, H-11) and δ_C_ 172.9 (11-OAc), δ_H_5.15 (1H, d, *J* = 10.8 Hz, H-12), and δ_C_ 168.4 (Tig-C-1). Two hydroxyl substituents were located at C-8 and C-14, respectively, based on their chemical shifts (δ_C_ 76.8 and 86.5) together with the molecular formula above. The existence of D-oleandrose (Ole) and 6-deoxy-3-*O*-methyl-D-allopyranose (Allo) units was confirmed by co-TLC and further followed by gas chromatography in comparison with standard monosaccharides. The sugar sequence and linkage position were established by the HMBC correlations of δ_H_ 4.72 (H-1 of Allo) to δ_C_ 84.2 (C-4 of Ole) and δ_H_ 4.65 (H-1 of Ole) to δ_C_ 79.3 (C-3 of aglycone), as shown in Figure 2.

Meanwhile, the NOE correlations of H-3/H-1*α*, H_3_-19/H-1*β*, H-12/H-9, and H-12/H-17 indicated the *α*-orientations of H-3/12/17. The ^3^ *J* coupling constant (*J* = 10.8 Hz) between H-11 and H-12 also confirmed their anti-periplanar relationship. Meanwhile, the *β* configurations of the two sugars were established based on the large coupling constants (each ^3^ *J*_1, 2_ > 7.0 Hz). Thus, the structure of 1 was established and named aspidataside A.

Compound 2 was obtained as a white amorphous powder. Its molecular formula was established as C_47_H_66_O_15_ according to the HR–ESI–MS spectrum at *m/z* [M + Na]^+^ 893.4305 (calculated for C_47_H_66_O_15_Na, 893.4299). An analysis of the ^1^H and ^13^CNMR data (Table 1; Table 2) displayed that the structure of 2 was similar to that of 1, except for the OAc group in 1 was absent in 2, extra signals of a benzoyl group at δ_H_7.80 (2H, dd, *J* = 7.2, 1.2 Hz), 7.43 (2H, t, *J* = 7.2 Hz), 7.58 (1H, t, *J* = 7.2 Hz), and δ_C_ 165.9, 128.0, 129.2, 129.0, and 129.3 were found in 2. In the HMBC spectrum, the correlations from δ_H_ 5.70 (H-11) to the carbonyl signal at δ_C_ 166.9 (Tig-1), and from δ_H_ 4.89 (H-12) to δ_C_ 165.9 indicated that the acetoxyl group at C-11 in 1 was replaced by the tigloyl group in 2, while the tigloyl group at C-12 was changed to the benzoyl group. The absolute configuration of 2 was established by the analysis of the NOESY and CD spectra. Therefore, compound 2 was identified and named aspidataside B.

Compound 3 was suggested to possess the molecular formula C_47_H_66_O_14_, based on the *m/z* 877.4348 [M + Na]^+^ (calculated for C_47_H_66_O_14_Na, 877.4350), in the HR–ESI–MS, which was 16 amu less than that of 2. In the NMR spectra of 3 and 2 (Table 1; Table 2), the structure of 3 corresponded to 2, except a hydroxyl group absent at C-14. The up-field chemical shift of C-14 at δ_C_ 48.0 in 3 compared with C-14 (δ_C_ 84.5) in 2 together with the molecular weight of 3 in the HR–ESI–MS exhibited the disappearance of hydroxyl function at C-14 in 3. The difference was fully confirmed by the 2D NMR experiments. With the analysis of NOESY data (Supporting Information, Supplementary Figure S23), compound 3 was determined as shown and named aspidataside C.

Compound 4, with the molecular formula of C_47_H_66_O_15_ determined by the HR–ESI–MS ion at *m/z* [M + H]^+^ 871.4461 (calculated for C_47_H_66_O_15_Na, 871.4480), was an isomer of 2. NMR data analysis between 4 and 2 suggested that they had almost identical structures except for the location of double bond in the B-ring portion. The double bond located at C-6/C-7 was confirmed by the correlations from δ_H_ 3.40 (m, H-5) to δ_C_ 55.2 (C-5), δ_H_ 6.50 (dd, *J* = 10.2 Hz, H-6) to δ_C_ 131.1 (C-7), and δ_H_ 6.60 (dd, *J* = 10.2 Hz, H-7) to δ_C_ 129.4 (C-7) in the HSQC spectrum, and correlations from H-6 to C-5 and C-8 in the HMBC experiment. By the combined use of ^1^H-^1^H COSY and NOESY experiments (Supporting Information, Supplementary Figures S31, S32), the structure of 4 was finally elucidated and named aspidataside D.

Compound 5 with the molecular formula of C_49_H_64_O_15_ was determined by the HR–ESI–MS (*m/z* 893.4305, calcd for C_49_H_64_O_15_Na [M + H]^+^, 893.4323) and had the similar structure as 4 based on comparison of their NMR data (Table 1; Table 2). The differences displayed that the tigloyl group in 4 was replaced by a benzoyl unit in 5. In the ^13^C NMR data, the typical signals at δ_C_ 166.1, 133.6, 128.8, 128.5, 130.5, 164.9, 129.6, 128.2, 129.1, and 133.2, and the absence signals at δ_C_ 14.6 and 12.1 supported the differences above. The additional benzoyl function was linked to C-12, based on the correlations of the HMBC spectrum. As a result, the structure of 5 was illustrated and named aspidataside E.

Compound 6 exhibited its molecular ion peak at *m/z* 877.3334 [M + Na]^+^, corresponding to the molecular formula of C_47_H_66_O_14_. The ^1^H NMR and ^13^C NMR (APT) data were strongly related to those of 4 (Table 1; Table 2). The difference was that the signal at C-8 (δ_C_ 77.8) in 4 was changed to δ_C_ 30.8 in 6. Considering the loosening of 16 units in molecular weight of 6, it was deduced that the hydroxyl group at C-8 in 4 was missed in 6, which was fully supported by the 2D NMR spectra (Supporting Information, Supplementary Figures S47–S50). Taken together with the NOESY data, compound **6** was established and named aspidataside F.

Compound 7 was given a molecular formula as C_47_H_68_O_14_ based on the HR–ESI–MS ion peak at *m/z* 875.4196 (calcd for C_47_H_68_O_14_Na [M + Na]^+^, 875.4207). Its NMR data were almost identical to those of 3, except for one less degree of unsaturation, which suggested the disappearance of one double bond. In the ^13^CNMR data (Table 2), the carbon signals at δ_C_ 74.4 (C-3), 36.6 (C-4), 56.2 (C-5), 26.3 (C-6), 36.6(C-7), and 18.3 (C-19) indicated that there was no double bond between C-5 and C-6 in 7. In the HMBC spectrum, the correlations from δ_H_ 1.17 (1H, m, H-5) to δ_C_ 26.3 (C-6) and 36.6(C-7) confirmed the difference. As a result, compound 7 was a ductile product of compound 3. Therefore, compound 7 was finally established and named aspidataside G.

Compound 8 was determined as C_49_H_66_O_14_ according to the HR–ESI–MS at m/z 905.4664 (calcd for C_49_H_66_O_14_Na [M + Na]+, 905.4750). The ^1^H NMR and ^13^C APT spectra displayed that 8 was analogous to 7 (Table 1; Table 2), except for the tigloyl group at C-11 in 7 was substituted by a benzoyl group (δ_C_ 165.4, 129.3, 128.9, 128.5, and 133.3) in 8. The 2D NMR of HMBC experiments fully confirmed the difference (Supporting Information, Supplementary Figures S65–S68). Together with its NOESY data, the structure of compound 8 was fully identified and named aspidataside H.

So far, the skeleton connections of A, B, C, and D rings in all pregnanes were *trans*/*trans*/*cis* types, and the CH_3_-18, CH_3_-19, 8-H, and 14-H configurations in all the structures were *β*-oriented (Liu et al., 2017; Pang et al., 2017; Li et al., 2019). Considering the identical biosyntenic relationship, and the comparison of the experimental and calculated CD data (Supporting Information, Supplementary Figures S49–S56), the absolute configurations of the chiral carbons in the pregnane skeleton were determined to be 3*S*, 8*S*, 9*S*, 10*R*, 11*S*, 12*S*, 13*S*, 14*R*, and 17*S*.

The known polyoxypregnanes were identified as sinomarinoside E (9) (Chen et al., 1999), deacylmetaplexigein (10) (Chen et al., 1999), incisagenin (11) (Chen et al., 1999), drevogenin P (12) (Niranjan et al., 2002), obcordata G (13) (Li et al., 2019), obcordata H (14) (Li et al., 2019), obcordata I (15) (Li et al., 2019), sarcostin (16) (Ma et al., 2017), caretroside A (17) (Halim and Khalil, 1996), and 3-*O*-[6-deoxy-3-*O*-methyl-β-allopyanosyl (1→4)-β-oleandropyranosyl]-5,6-dihydro gen-11α,12β-di-*O*-tigloyl-17β-marsdenin (18) (Liu et al., 2017), based on the comparison of the observed spectroscopic data with those reported in the literature.

### 2.2 Inhibitory Effects of Compounds 1–18 on Human HL-60 Cell Line

The cytotoxic activity of the isolates (1–18) against HL-60 human promyelocytic acute leukemia cell *in vitro* was evaluated using the MTT method with the positive control doxorubicin IC_50_ 3.87 μM, and the results are shown in Table 3. Compounds 2 and 9 exhibited potent activities with IC_50_ values of 8.03 ± 0.35 and 9.25 ± 0.45 μM, respectively. Compounds 1 and 3 displayed moderate activity with IC_50_ values ranging from 10.53 to 13.27 μM, and approximately two-fold more active than the compounds 7–8, 13–15, 17, and 18, and about three-fold more active comparing with the compounds 4–6, 10, and 11. Compounds 12 and 16 showed week cytotoxic activity against anti–HL-60 cell (IC_50_ > 50 *μ*M).

**TABLE 3:** *In vitro* cytotoxic activity of the compounds (1–18) against HL-60 cell.

Compound	IC_50_ (*μ*M)	Compound	IC_50_ (*μ*M)
1	13.27±0.64^a^	10	41.56±4.12
2	8.03±0.35	11	40.85±3.67
3	10.53±0.92	12	57.33±4.62
4	30.72±2.15	13	21.68±1.83
5	32.80±1.80	14	24.86±2.35
6	32.40±1.28	15	27.50±2.24
7	29.13±2.02	16	55.62±4.83
8	24.94±2.55	17	29.45±2.50
9	9.25±0.45	18	26.36±2.48
Doxorubicin^b^	3.87±0.28	Doxorubicin	3.87±0.28

a Value presents mean ± SD of triplicate experiments.

b Positive control substance.

### 2.3 Structure–Activity Relationships

**FIGURE 3:**
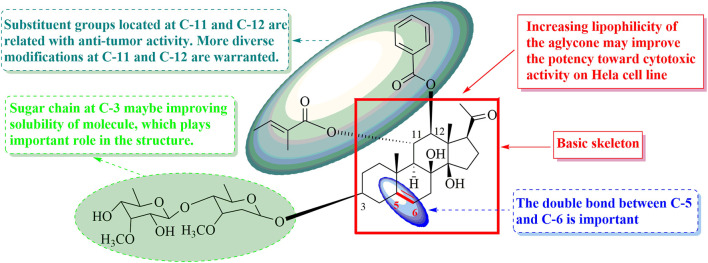
Graphical explanation of the general SAR for cytotoxic activity of 2 and its derivatives.

The structure–activity relations of polyoxypregnane derivatives were described, as shown in Figure 3. It was found that all the isolates exhibited moderate inhibitory activity against HL-60 cell lines, which may be due to their unique pregen-5-en-20-one skeleton. It was reported that pregnane, as a component of steroids, plays an important role in regulating the metabolism of life. However, the presence of sugar moieties in the side chain also influenced the cytotoxic activity. Compounds 10–12, and 16 without sugar chain on the hydroxyl at C-3 displayed weak activity (39.0 < IC_50_ < 60.0 μM) when comparing with possessing oligosaccharides located at C-3, such as compounds 1–8, 9, 13–15, 17, and 18 with IC_50_ values ranging from 8.03 to 32.80 μM. These results indicated that the presence of sugar moiety at C-3 maybe improving the solubility of structures and could significantly enhance the cytotoxic activity, and played important roles in the structure.

Four (1–3, and 9, 8.03 < IC_50_ < 13.27 μM) compounds with a double bond between C-5 and C-6 showed the best potency among the 14 isolated constituents with sugar side chain. Compounds 7–8, 13–15, and 17–18 (21.68 < IC_50_ < 29.45 μM, vs. 1–3, and 9) characterizing without a double bond in skeleton exhibited moderate cytotoxic activity. In addition, the cytotoxic activities were obviously decreased due to a double bond at C-6/C-7, such as compounds 4–6 with the IC_50_ values from 30.72 to 32.80 μM comparing with 1–3 and 9. Thus, it is likely that the presence and location of double bond was important to the activity in this type of compounds. Furthermore, substituent groups, including *O*-Bz, *O*-Ac, and *O*-Tig, located at C-11 and C-12 were related with antitumor activity. More diverse modifications at C-11 and C-12 are warranted. These findings suggested that the sugar chain, the location of double bond, and polyester substitution in the C-11 and C-12 can substantially contribute to improve their cytotoxic activity.

In summary, the most promising compound, that is, compound 2, exerted significant cytotoxicity against the tested cancerous cell, and might be considered as a potential candidate for drug development and worthy of more detailed studies.

## 3 Conclusion

In conclusion, chemical investigation of the stems of *A. obcordata* has led to the discovery of eight new polyoxypregnanes (1–8) and ten known derivatives (9–18). In the *in vitro* assays, compounds 4–8 and 10–18 showed weak activity against HL-60 cells, while compounds 1–3 and 9 displayed strong inhibitory effects toward HL-60. Especially, compound 2 exhibited the best inhibition of HL-60 with an IC_50_ value of 8.03 μM, which was likely due to its hydrophilic sugar chains and bulky hydrophobic aliphatic chains linked with the complex conjugated system by SAR analysis. Nevertheless, the antitumor mechanism of action of compound 2 as anti–HL-60 agent is necessary for further studies.

## 4 Experimental

### 4.1 General Experimental Procedures

Optical rotations were measured with a Perkin-Elmer 341 digital polarimeter (PerkinElmer, Norwalk, CT, United States). UV and IR spectra were obtained on Shimadzu UV2550 and FTIR-8400S spectrometers (Shimadzu, Kyoto, Japan), respectively. CD spectra were obtained using a JASCO J-815 spectropolarimeter (Tokyo, Japan). NMR spectra were obtained using a Bruker AV III 600 NMR spectrometer with chemical shift values presented as *δ* values with TMS as the internal standard (Bruker, Billerica, German). HR–ESI–MS were performed on an LTQ-Orbitrap XL spectrometer (Thermo Fisher Scientific, Boston, MA, United States). Column chromatography (CC) was performed using silica gel (100–200 and 200–300 mesh, Qingdao Marine Chemical Plant, Qingdao, China). TLC analyses were carried out on silica gel GF_254_ precoated plates (Zhi Fu Huang Wu PilotPlant of Silica Gel Development, Yantai, China) with detection accomplished by spraying with 5% H_2_SO_4_ followed by heating at 100°C. Preparative HPLC was performed on a Lumtech K-1001 analytic LC equipped with two K-501 pumps, a K-2600 UV detector, and a YMC Pack C_18_ semi-preparative column (250 mm × 10 mm, i. d., 5 μM, YMC Co. Ltd., Japan), and eluted with CH_3_OH–H_2_O at a flow rate of 2 ml/min. All solvents used were of analytical grade (Beijing Chemical Works).

### 4.2 Plant Material

The vines of *A. obcordata* were collected in September 2016 from Jinghong, Yunnan Province, People’s Republic of China and identified by Prof. Dai-Gui Zhang, at the School of Biological Resources and Environmental Science, Jishou University, where a voucher specimen (JS161248) was deposited.

### 4.3 Extraction and Isolation

The dried and powdered vines of *A. obcordata* (4.0 kg) were extracted with 95% EtOH (20 L) three times (each time for 1 h). The solvents were filtrated and evaporated under reduced pressure to give the total extract (249.0 g), and dissolved successively with water (1.5 L), in the order, petroleum ether (MSO), dichloromethane (DCM), ethyl acetate (EtOAc), and n-butanol (NBA) to re-extract the water solution of the crude extract, and then have yielded diﬀerent fractions. EtOAc fraction was selected for further separation due to its better inhibitory effect against HL-60 cells.

The EtOAc fraction (112.0 g) was subjected to CC over silica gel (100–200 mesh, 10 × 150 cm), eluting with a gradient of CH_2_Cl_2_–MeOH (from 1:0 to 0:1), and obtained twelve fractions, A–L. Fraction A (7.8 g) was subjected to chromatography using ODS MPLC elution with MeOH–H_2_O (30:70, 70:30, 90:10, and 100:0; v/v), to yield four fractions (Fr. A1–4), and Fr. A2 (0.5280 g) was separated through semi-preparative HPLC using a mobile phase of MeOH–H_2_O (70:30, v/v) to afford 12 (14.6 mg, *R*_t_ 23.5 min) and 16 (13.8 mg, *R*_t_ 28.8 min). Compounds 10 (17.2 mg, *R*_t_ 20.4) and 11 (13.2 mg, *R*_t_ 25.6) were isolated from Fr. A3 (0.2468 g) purified with MeOH–H_2_O (75:25, v/v). Fraction D (10.4 g) was subjected over ODS column chromatography elution with MeOH–H_2_O in a gradient manner (30:70, 50:50, 70:30, 80:20, and 100:0; v/v) to give five sub-fractions (Fr. D1–D5). Fr. D2 (0.348 g) was purified by HPLC with an isocratic of 55% MeOH–H_2_O on a YMC C_18_ column to get compounds 1 (8.5 mg, *R*_t_ 13.1 min), 2 (9.8 mg, *R*_t_ 16.3 min), 6 (10.4 mg, *R*_t_ 21.6 min), and 9 (7.5 mg, *R*_t_ 28.4 min). Compounds 3 (8.6 mg, *R*_t_ 18.2 min), 4 (7.5 mg, *R*_t_ 22.4 min), and 5 (12.2 mg, *R*_t_ 27.8 min) were yielded from Fr. D3 (0.41 g) separated by semi-preparative HPLC with MeOH–H_2_O (50:50, v/v). Similarly, Fr. E (5.8 g) was isolated through ODS MPLC elution with MeOH–H_2_O (30:70, 50:50, 70:30, 90:10; 100:0, v/v) to give Fr. E1–5, and Fr. E2 (0.55 g) was prepared by semi-preparative HPLC elution with MeOH–H_2_O (55:45, v/v), to obtain compounds 7 (14.5 mg, *R*_t_ 18.2 min), 8 (10.6 mg, *R*_t_ 22.3 min), 13 (8.2 mg, *R*_t_ 28.5 min), and 18 (14.8 mg, *R*_t_ 31.5 min). Compounds 14 (15.4 mg, *R*_t_ 17.5 min), 15 (7.8 mg, *R*_t_ 21.5 min), and 17 (13.5 mg, *R*_t_ 27.8 min) were yielded from Fr. E3 (0.3550 g) separated by semi-preparative HPLC with MeOH–H_2_O (50:50, v/v).

### 4.4 New Compounds

#### 4.4.1 Aspidataside A

C_42_H_64_O_15_, white amorphous powder; [*α*]^20^_D_+35^o^ (*c* 0.15, MeOH); UV *λ*_max_ (MeOH) nm (log *ε*): 225 and 272; IR (KBr) ν_max_ cm^−1^: 3,423, 1,660; HR–ESI–MS *m/z* 831.4139 (calculated for 831.4143 C_42_H_64_NaO_15_). ^1^H-NMR (MeOD, 600 MHz) and ^13^C-NMR (MeOD, 150 MHz) (see Table 1; Table 2).

#### 4.4.2 Aspidataside B

C_47_H_66_O_15_, white amorphous powder; [*α*]^20^_D_+42.5^o^ (*c* 0.12, MeOH); UV *λ*_max_ (MeOH) nm (log *ε*): 272; IR (KBr) ν_max_ cm^−1^: 3,350, 1,665, 1,605, 1,525; HR–ESI–MS *m/z* [M + Na]^+^ 893.4305 (calculated for C_47_H_66_NaO_15_, 893.4299). ^1^H-NMR (MeOD, 600 MHz) and ^13^C-NMR (DMSO, 150 MHz) (see Table 1; Table 2).

#### 4.4.3 Aspidataside C

C_47_H_66_O_14_, white amorphous powder; [*α*]^20^_D_+40.2^o^ (*c* 0.15, MeOH); UV *λ*_max_ (MeOH) nm (log *ε*): 275; IR (KBr) ν_max_ cm^−1^: 3,318, 1,680, 1,600, 1,524; HR–ESI–MS *m/z* 877.4348 [M + Na]^+^ (calculated for C_47_H_66_NaO_14_, 877.4350). ^1^H-NMR (MeOD, 600 MHz) and ^13^C-NMR (DMSO, 150 MHz) (see Table 1 and Table 2).

#### 4.4.4 Aspidataside D

C_47_H_66_O_15_, white amorphous powder; [*α*]^20^_D_+52.5^o^ (*c* 0.12, MeOH); UV *λ*_max_ (MeOH) nm (log *ε*): 274; IR (KBr) ν_max_ cm^−1^: 3,320, 1,675, 1,604, 1,525; HR–ESI–MS *m/z* 871.4461 [M + H]^+^ (calculated for C_47_H_66_NaO_15_, 871.4480). ^1^H-NMR (MeOD, 600 MHz) and ^13^C-NMR (DMSO, 150 MHz) see Table 1 and Table 2.

#### 4.4.5 Aspidataside E

C_49_H_64_O_15_, white amorphous powder; [*α*]^20^_D_+44.8^o^ (*c* 0.10, MeOH); UV *λ*_max_ (MeOH) nm (log *ε*): 275; IR (KBr) ν_max_ cm^−1^: 3,315, 1,678, 1,605, 1,520; HR–ESI–MS *m/z* 893.4305 [M + H]^+^ (calculated for C_49_H_64_NaO_15_, 893.4323). ^1^H-NMR (MeOD, 600 MHz) and ^13^C-NMR (DMSO, 150 MHz) see Table 1 and Table 2.

#### 4.4.6 Aspidataside F

C_47_H_66_O_14_, white amorphous powder; [*α*]^20^_D_+50.3^o^ (*c* 0.15, MeOH); UV *λ*_max_ (MeOH) nm (log *ε*): 272; IR (KBr) ν_max_ cm^−1^: 3,324, 1,692, 1,600, 1,524; HR–ESI–MS *m/z*877.3334 [M + Na]^+^ (calculated for C_47_H_66_NaO_14_, 877.3350). ^1^H-NMR (MeOD, 600 MHz) and ^13^C-NMR (DMSO, 150 MHz) (see Table 1 and Table 2).

#### 4.4.7 Aspidataside G

C_47_H_68_O_14_, white amorphous powder; [*α*]^20^_D_+60.4^o^ (*c* 0.15, MeOH); UV *λ*_max_ (MeOH) nm (log *ε*): 275; IR (KBr) ν_max_ cm^−1^: 3,384, 1,689, 1,610, 1,532; HR–ESI–MS *m/z* 875.4196 [M + Na]^+^ (calculated for C_47_H_68_NaO_14_, 875.4207). ^1^H-NMR (MeOD, 600 MHz) and ^13^C-NMR (DMSO, 150 MHz) (see Table 1 and Table 2).

#### 4.4.8 Aspidataside H

C_49_H_66_O_14_, white amorphous powder; [*α*]^20^_D_+45.2^o^ (*c* 0.15, MeOH); UV *λ*_max_ (MeOH) nm (log *ε*): 275; IR (KBr) ν_max_ cm^−1^: 3,372, 1,674, 1,538; HR–ESI–MS *m/z* 905.4664 [M + Na]^+^ (calculated for C_49_H_66_NaO_14_, 905.4750). ^1^H-NMR (MeOD, 600 MHz) and ^13^C-NMR (DMSO, 150 MHz) (see Table 1 and Table 2).

Acid hydrolysis of compounds was accomplished by the procedure described previously (Wang et al., 2019).

### 4.6 *In vitro* Cytotoxicity Bioassay

Compounds 1–18 were assessed for cytotoxicity against human cancer cell line HL-60. This used the MTT method as described in the previously published literature (Halim and Khalil, 1996). Briefly, cells were grown in DMEM supplied with 10% fetal bovine serum and cultured at a density of 1.1 × 10^5^ cells/ml per well in a 96-well plate at 37°C in a 5% CO_2_ incubator overnight. Each concentration was tested in triplicate. Then, the cells were incubated with 10 µL of MTT (5 mg/ml) for additional 4 h. The residual liquid was removed, and 200 µL DMSO was added. The absorbance was recorded on a microplate reader at a wavelength of 570 nm. The experiments were conducted a minimum of three times.

### 4.7 Statistical Analysis

Each experiment was repeated for at least three times. Results were expressed as mean ± SD. The statistical significance of differences between groups was evaluated by the unpaired Student’s *t*-test and indicated with (**) *p* < 0.01, (*) *p* < 0.05.

Statistical software SPSS version 15.0 was used for statistical analysis. ANOVA analysis was used with Fisher’s LSD multiple comparison test for multiple comparisons. All *p*-values < 0.05 were considered statistically significant.

## Data Availability

The original contributions presented in the study are included in the article/Supplementary Materials; further inquiries can be directed to the corresponding author.
