# Supplementary material for: Discovery of Polyoxypregnane Derivatives From Aspidopterys obcordata With Their Potential Antitumor Activity
Source: Front Chem. 2022 Jan 5;9:799911. doi: 10.3389/fchem.2021.799911 (PMC8766633; doi:10.3389/fchem.2021.799911)

Discovery polyoxypregnane derivatives from *Aspidopterys obcordata* with their potential anti-tumor activity

Hong-Wei Guo [1], Yun-Gang Tian [1], Yi-Han Liu [1], Jia Huang [1], Jian-Xia Wang [3], Hua Long [1], Hua Wei [2, 4,*]

Affiliation

[a] College of Biology and Environmental Science, Jishou University, Jishou 416000, China

[b] School of Pharmaceutical Sciences, Jishou University, Jishou 416000, China

[c] School of Medicine, Jishou University, Jishou 416000, China

[d] Tujia Medicine Research Center in Hunan (Jishou University), Jishou 416000, China

*Corresponding author. Tel./fax: + 86 0743-8564416.

E-mail address: weihua20@126.com

List of Figures S1-S68

Figure S1. ^{1}H-NMR (600 MHz, MeOD) spectrum of the new compound **1**

Figure S2. ^{13}C-APT (150 MHz, MeOD) spectrum of the new compound **1**

Figure S3. HSQC spectrum of the new compound **1**

Figure S4. HMBC spectrum of the new compound **1**

Figure S5. ^{1}H-^{1}H COSY spectrum of the new compound **1**

Figure S6. NOESY spectrum of the new compound **1**

Figure S7. IR spectrum of the new compound **1**

Figure S8. HRESIMS spectrum of the new compound **1**

Figure S9. ^{1}H-NMR (600 MHz, DMSO) spectrum of the new compound **2**

Figure S10. ^{13}C-APT (150 MHz, DMSO) spectrum of the new compound **2**

Figure S11. HSQC spectrum of the new compound **2**

Figure S12. HMBC spectrum of the new compound **2**

Figure S13. ^{1}H-^{1}H COSY spectrum of the new compound **2**

Figure S14. NOESY spectrum of the new compound **2**

Figure S15. IR spectrum of the new compound **2**

Figure S16. HRESIMS spectrum of the new compound **2**

Figure S17. Key HMBC and ^{1}H-^{1}H COSY correlations of the new compound **2**

Figure S18. ^{1}H-NMR (600 MHz,DMSO) spectrum of the new compound **3**

Figure S19. ^{13}C-APT (150 MHz, DMSO) spectrum of the new compound **3**

Figure S20. HSQC spectrum of the new compound **3**

Figure S21. HMBC spectrum of the new compound **3**

Figure S22. ^{1}H-^{1}H COSY spectrum of the new compound **3**

Figure S23. NOESY spectrum of the new compound **3**

Figure S24. IR spectrum of the new compound **3**

Figure S25. HRESIMS spectrum of the new compound **3**

Figure S26. Key HMBC and ^{1}H-^{1}H COSY correlations of the new compound **3**

Figure S27. ^{1}H-NMR (600 MHz, DMSO) spectrum of the new compound **4**

Figure S28. ^{13}C-APT (150 MHz, DMSO) spectrum of the new compound **4**

Figure S29. HSQC spectrum of the new compound **4**

Figure S30. HMBC spectrum of the new compound **4**

Figure S31. ^{1}H-^{1}H COSY spectrum of the new compound **4**

Figure S32. NOESY spectrum of the new compound **4**

Figure S33. IR spectrum of the new compound **4**

Figure S34. HRESIMS spectrum of the new compound **4**

Figure S35. Key HMBC and ^{1}H-^{1}H COSY correlations of the new compound **4**

Figure S36. ^{1}H-NMR (600 MHz, DMSO) spectrum of the new compound **5**

Figure S37. ^{13}C-APT (150 MHz, DMSO) spectrum of the new compound **5**

Figure S38. HSQC spectrum of the new compound **5**

Figure S39. HMBC spectrum of the new compound **5**

Figure S40. ^{1}H-^{1}H COSY spectrum of the new compound **5**

Figure S41. NOESY spectrum of the new compound **5**

Figure S42. IR spectrum of the new compound **5**

Figure S43. HRESIMS spectrum of the new compound **5**

Figure S44. Key HMBC and ^{1}H-^{1}H COSY correlations of the new compound **5**

Figure S45. ^{1}H-NMR (600 MHz, DMSO) spectrum of the new compound **6**

Figure S46. ^{13}C-APT (150 MHz, DMSO) spectrum of the new compound **6**

Figure S47. HSQC spectrum of the new compound **6**

Figure S48. HMBC spectrum of the new compound **6**

Figure S49. ^{1}H-^{1}H COSY spectrum of the new compound **6**

Figure S50. NOESY spectrum of the new compound **6**

Figure S51. IR spectrum of the new compound **6**

Figure S52. HRESIMS spectrum of the new compound **6**

Figure S53. Key HMBC and ^{1}H-^{1}H COSY correlations of the new compound **6**

Figure S54. ^{1}H-NMR (600 MHz, DMSO) spectrum of the new compound **7**

Figure S55. ^{13}C-APT (150 MHz, DMSO) spectrum of the new compound **7**

Figure S56. HSQC spectrum of the new compound **7**

Figure S57. HMBC spectrum of the new compound **7**

Figure S58. ^{1}H-^{1}H COSY spectrum of the new compound **7**

Figure S59. NOESY spectrum of the new compound **7**

Figure S60. IR spectrum of the new compound **7**

Figure S61. HRESIMS spectrum of the new compound **7**

Figure S62. Key HMBC and ^{1}H-^{1}H COSY correlations of the new compound **7**

Figure S63. ^{1}H-NMR (600 MHz,DMSO) spectrum of the new compound **8**

Figure S64. ^{13}C-APT (150 MHz, DMSO) spectrum of the new compound **8**

Figure S65. HSQC spectrum of the new compound **8**

Figure S66. HMBC spectrum of the new compound **8**

Figure S67. ^{1}H-^{1}H COSY spectrum of the new compound **8**

Figure S68. NOESY spectrum of the new compound **8**

Figure S69. IR spectrum of the new compound **8**

Figure S70. HRESIMS spectrum of the new compound **8**

Figure S71. Key HMBC and ^{1}H-^{1}H COSY correlations of the new compound **8**

Figure S72. Calculated and experimental ECD spectrum of **1**

Figure S73. Calculated and experimental ECD spectrum of **2**

Figure S74. Calculated and experimental ECD spectrum of **3**

Figure S75. Calculated and experimental ECD spectrum of **4**

Figure S76. Calculated and experimental ECD spectrum of **5**

Figure S77. Calculated and experimental ECD spectrum of **6**

Figure S78. Calculated and experimental ECD spectrum of **7**

Figure S79. Calculated and experimental ECD spectrum of **8**

Figure S1. ^{1}H-NMR (600 MHz, MeOD) spectrum of the new compound **1**

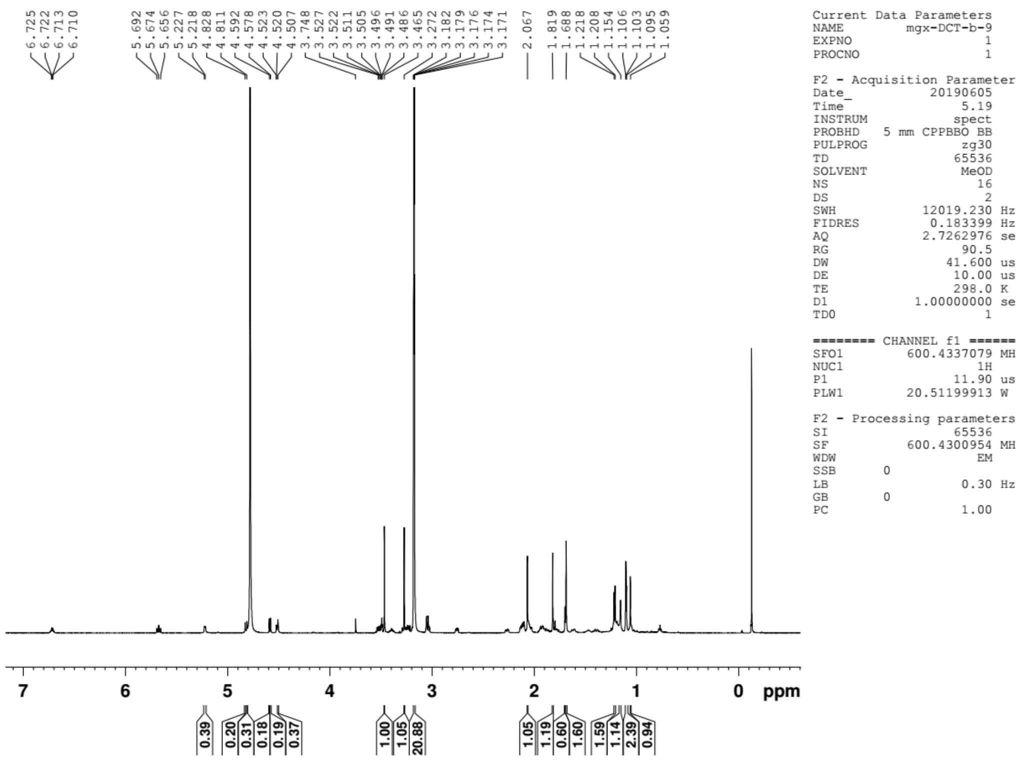

Figure S2. ^{13}C-APT (150 MHz, MeOD) spectrum of the new compound **1**

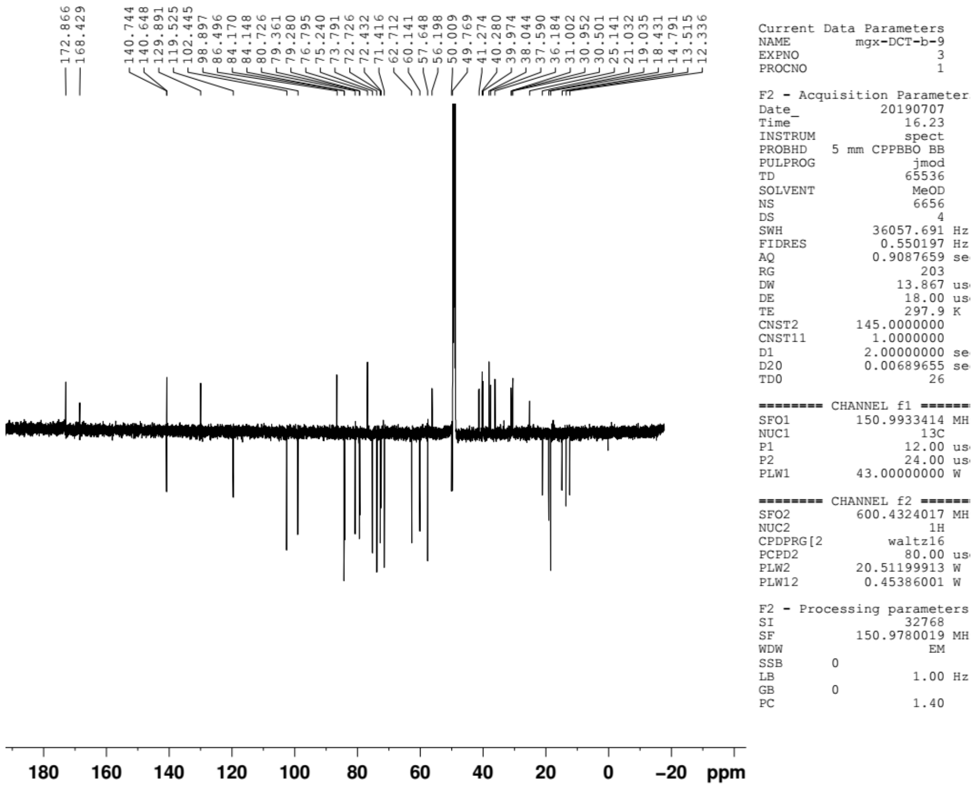

Figure S3. HSQC spectrum of the new compound **1**

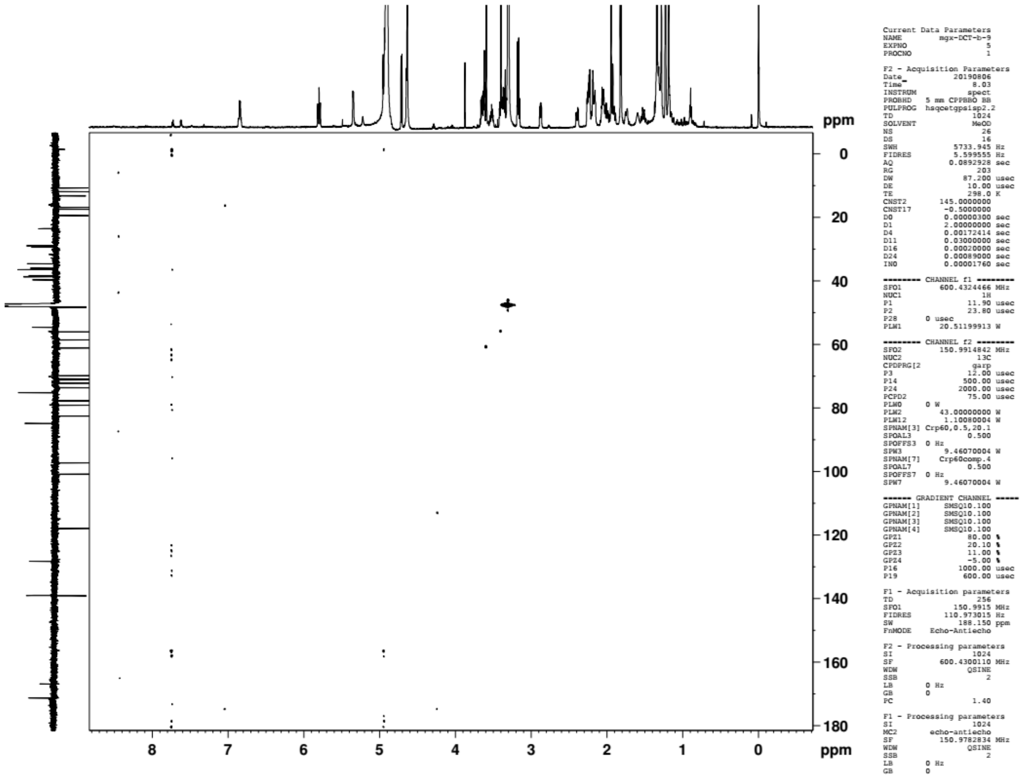

Figure S4. HMBC spectrum of the new compound **1**

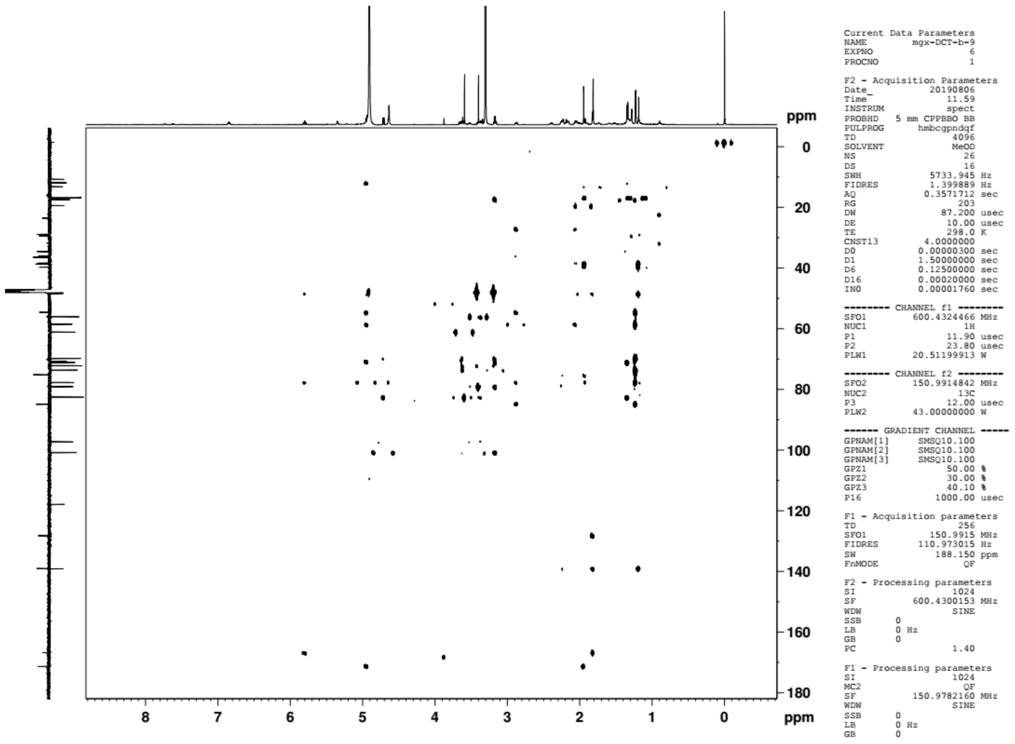

Figure S5. ^{1}H-^{1}H COSY spectrum of the new compound **1**

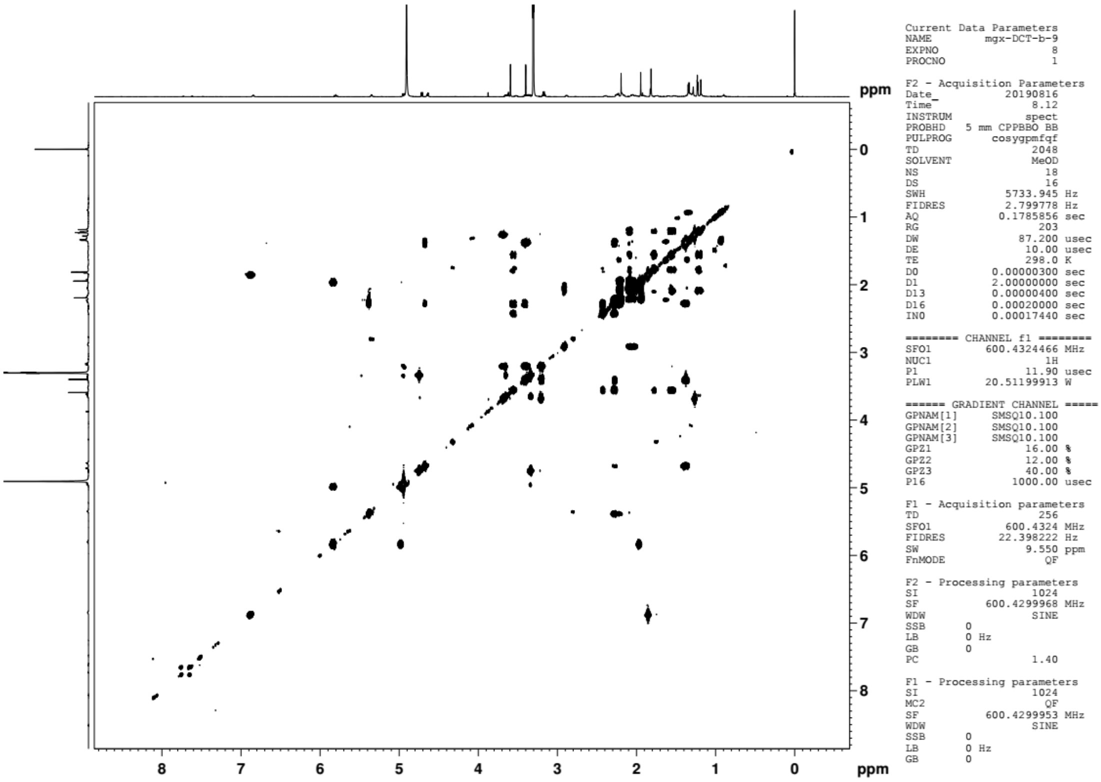

Figure S6. NOESY spectrum of the new compound **1**

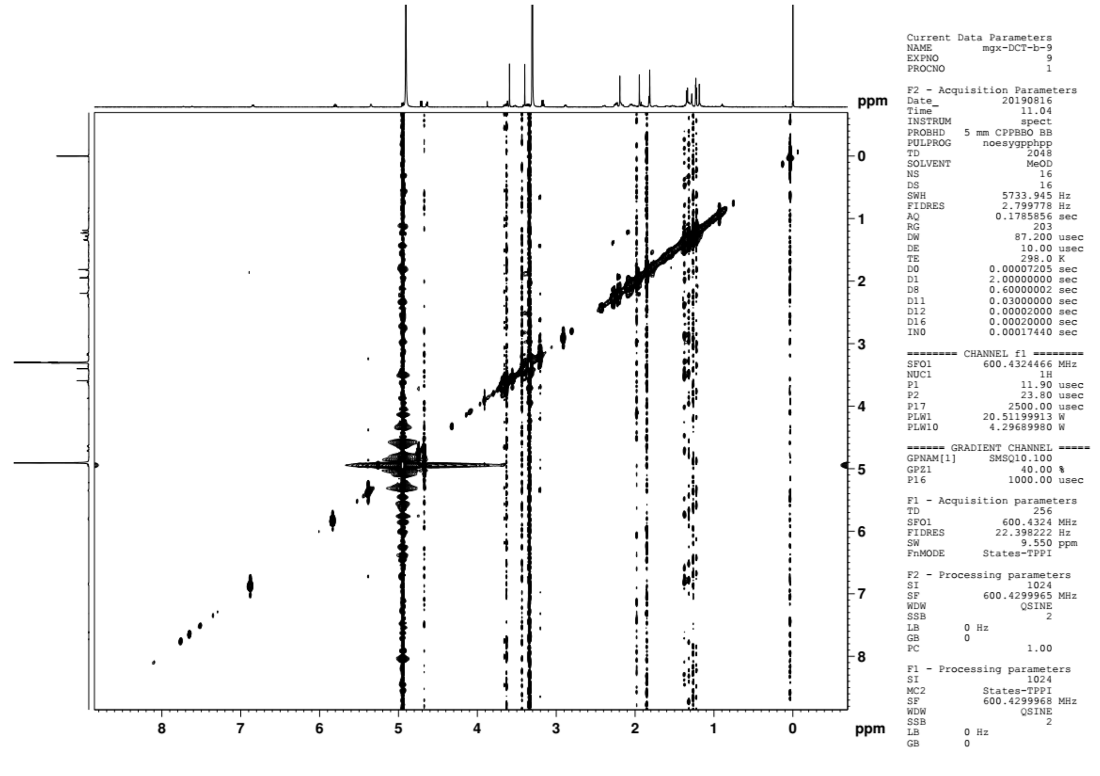

Figure S7. IR spectrum of the new compound **1**

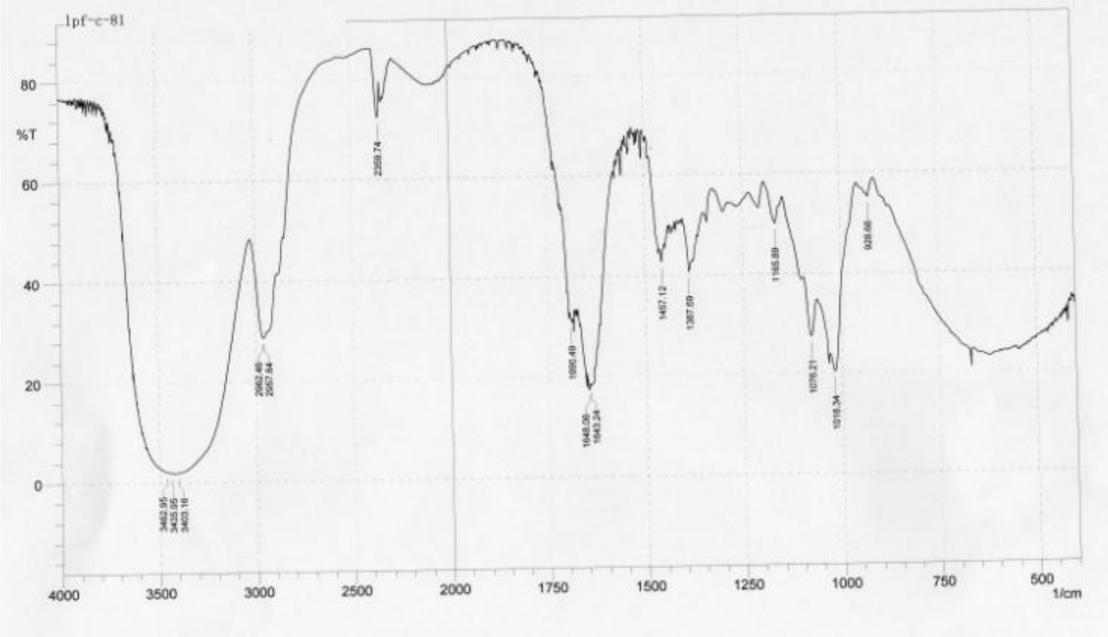

Figure S8. HRESIMS spectrum of the new compound **1**

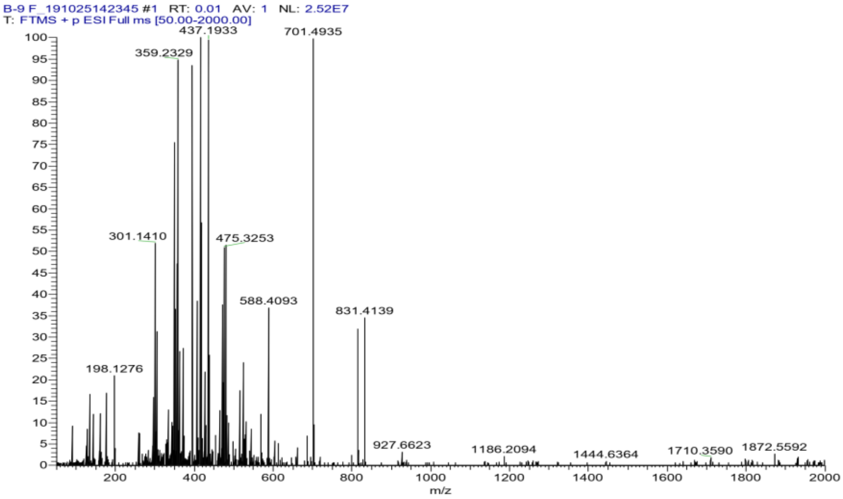

Figure S9. ^{1}H-NMR (600 MHz, DMSO) spectrum of the new compound **2**

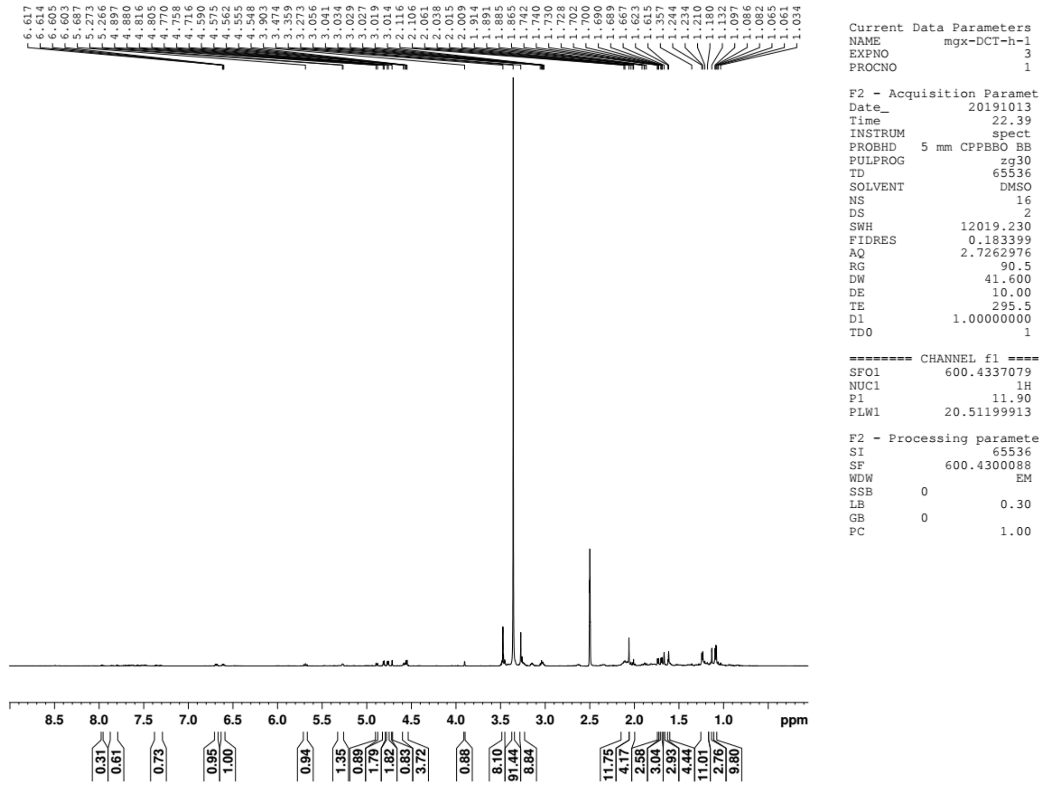

Figure S10. ^{13}C-APT (150 MHz, DMSO) spectrum of the new compound **2**

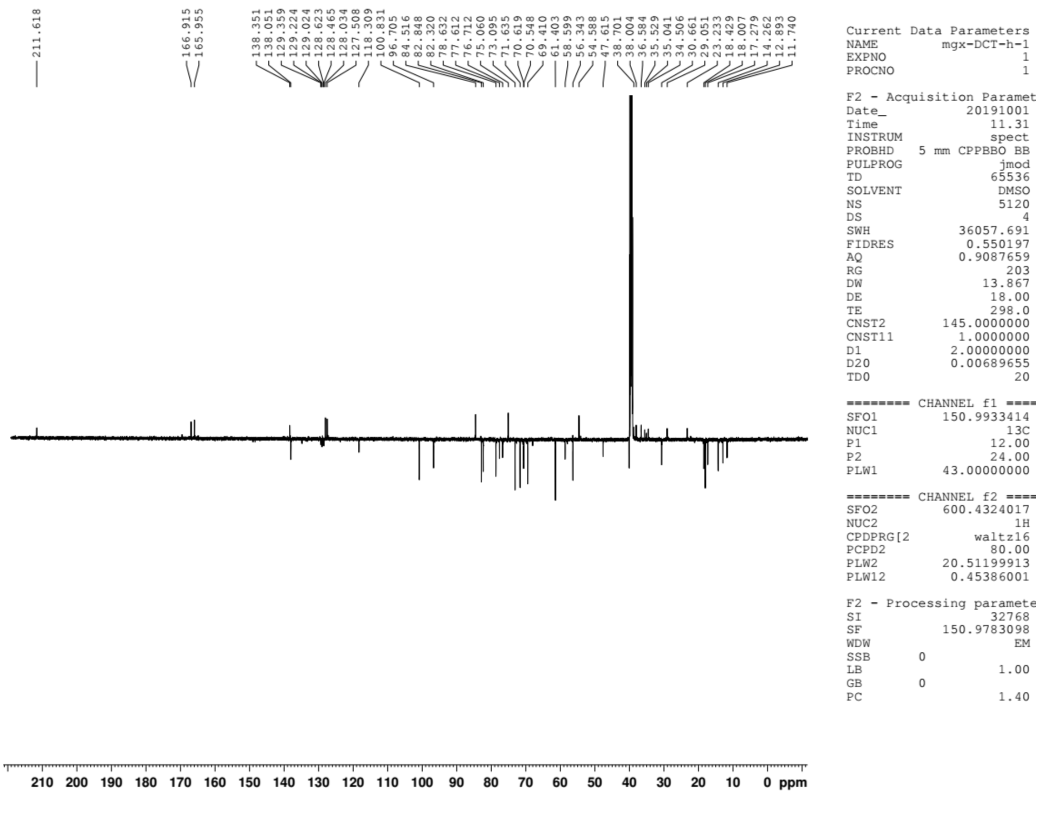

Figure S11. HSQC spectrum of the new compound **2**

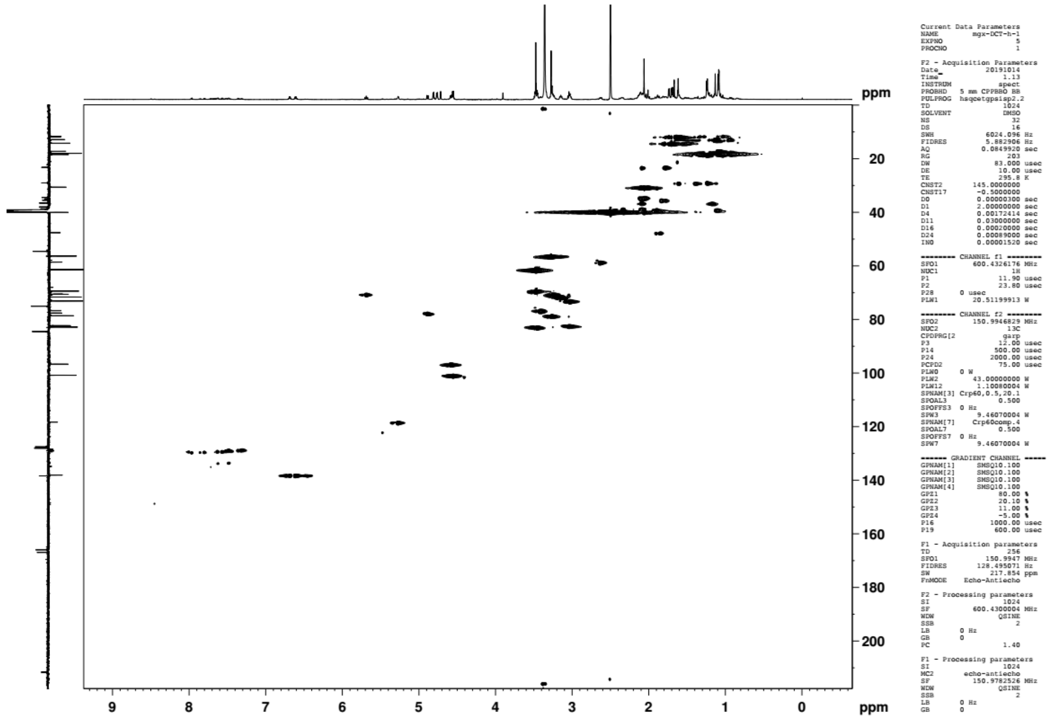

Figure S12. HMBC spectrum of the new compound **2**

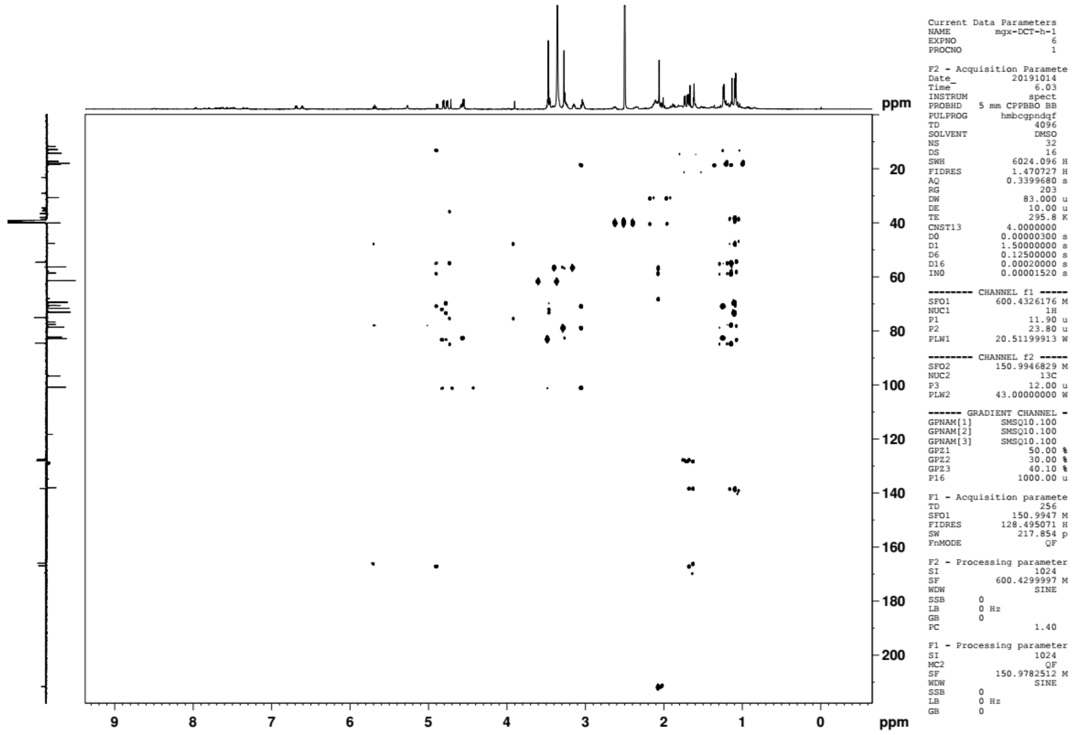

Figure S13. ^{1}H-^{1}H COSY spectrum of the new compound **2**

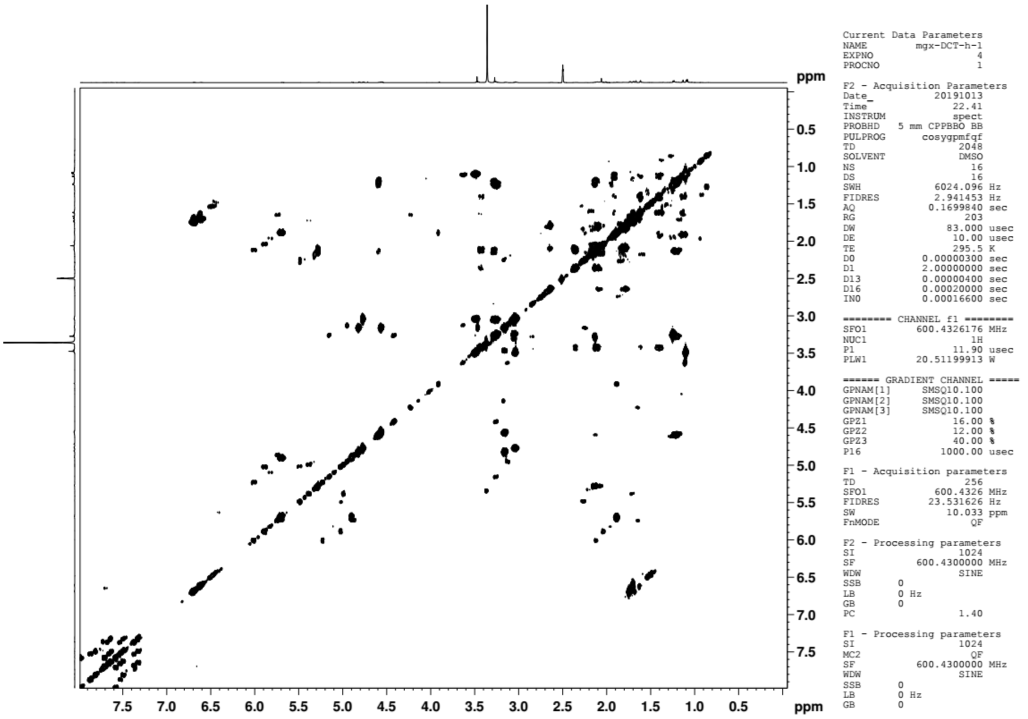

Figure S14. NOESY spectrum of the new compound **2**

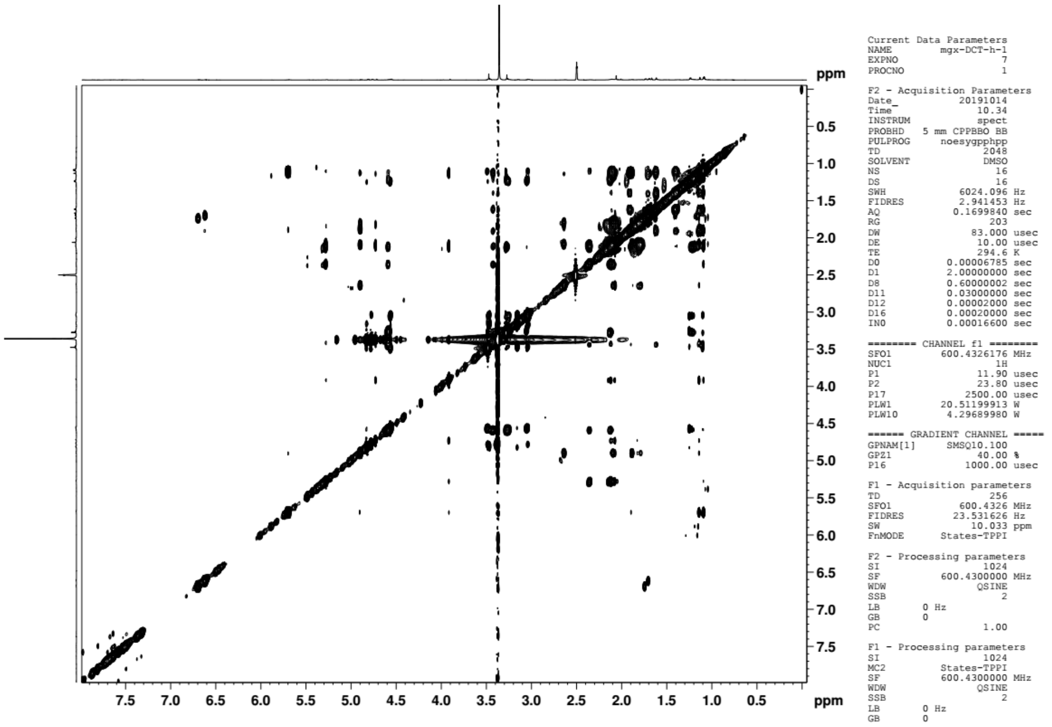

Figure S15. IR spectrum of the new compound **2**

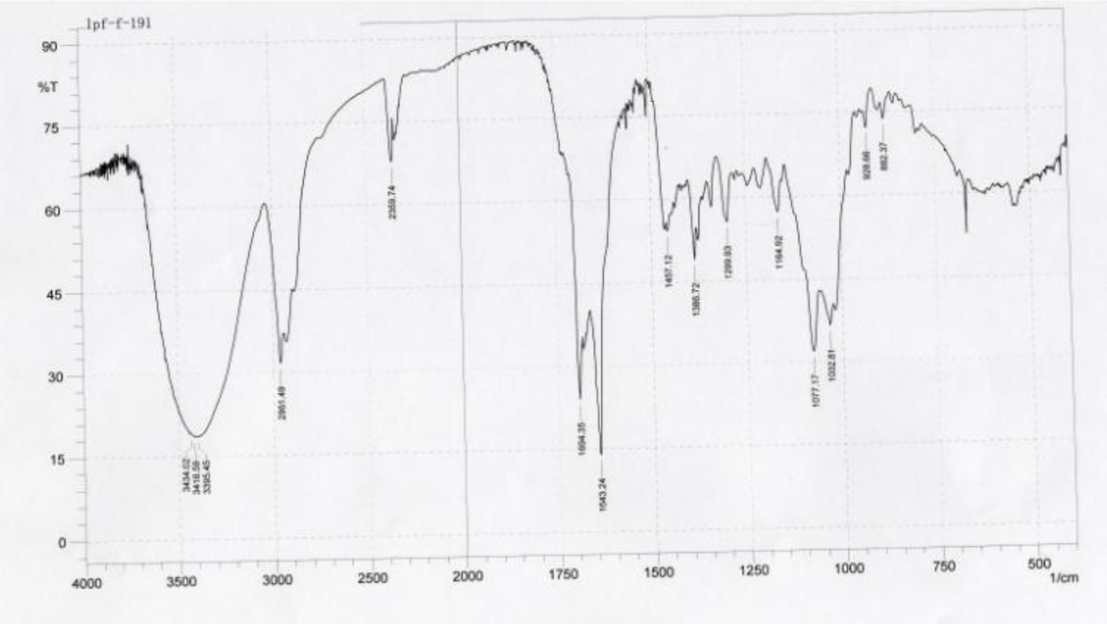

Figure S16. HRESIMS spectrum of the new compound **2**

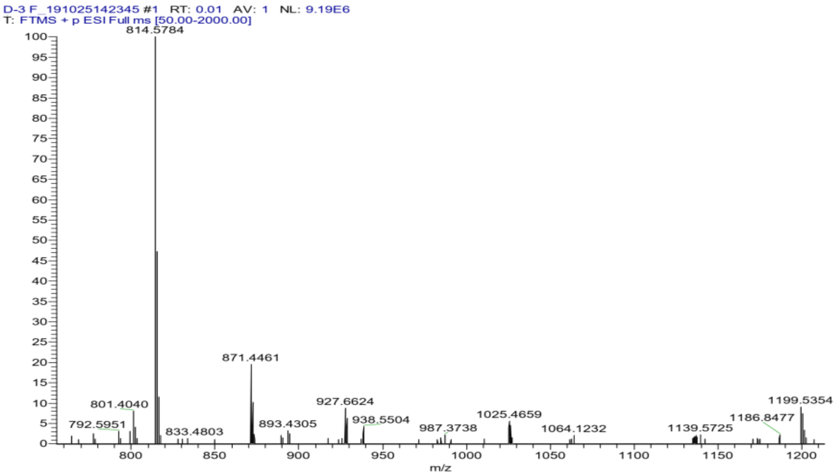

Figure S17. Key HMBC (Arrows) and ^{1}H-^{1}H COSY (Bonds) correlations of the new compound **2**

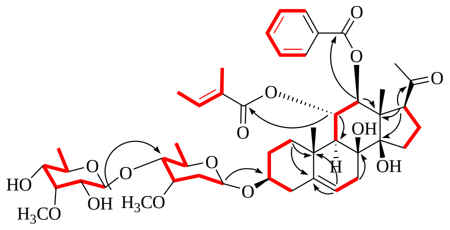

Figure S18. ^{1}H-NMR (600 MHz,DMSO) spectrum of the new compound **3**

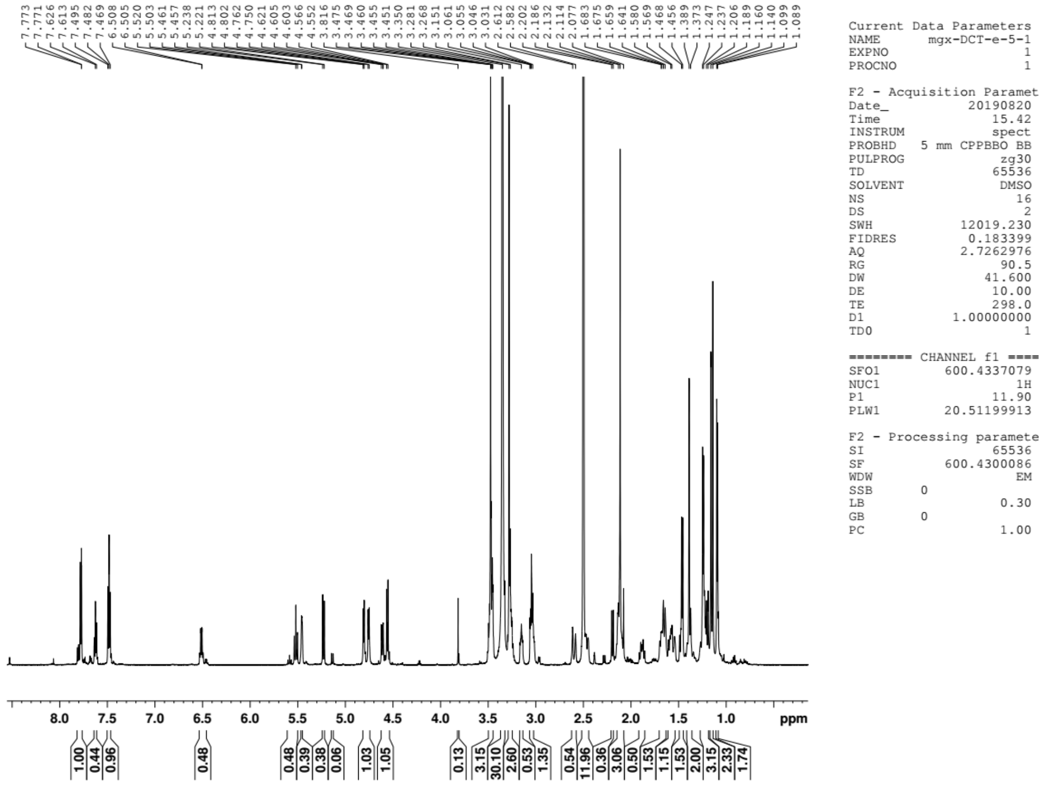

Figure S19. ^{13}C-APT (150 MHz, DMSO) spectrum of the new compound **3**

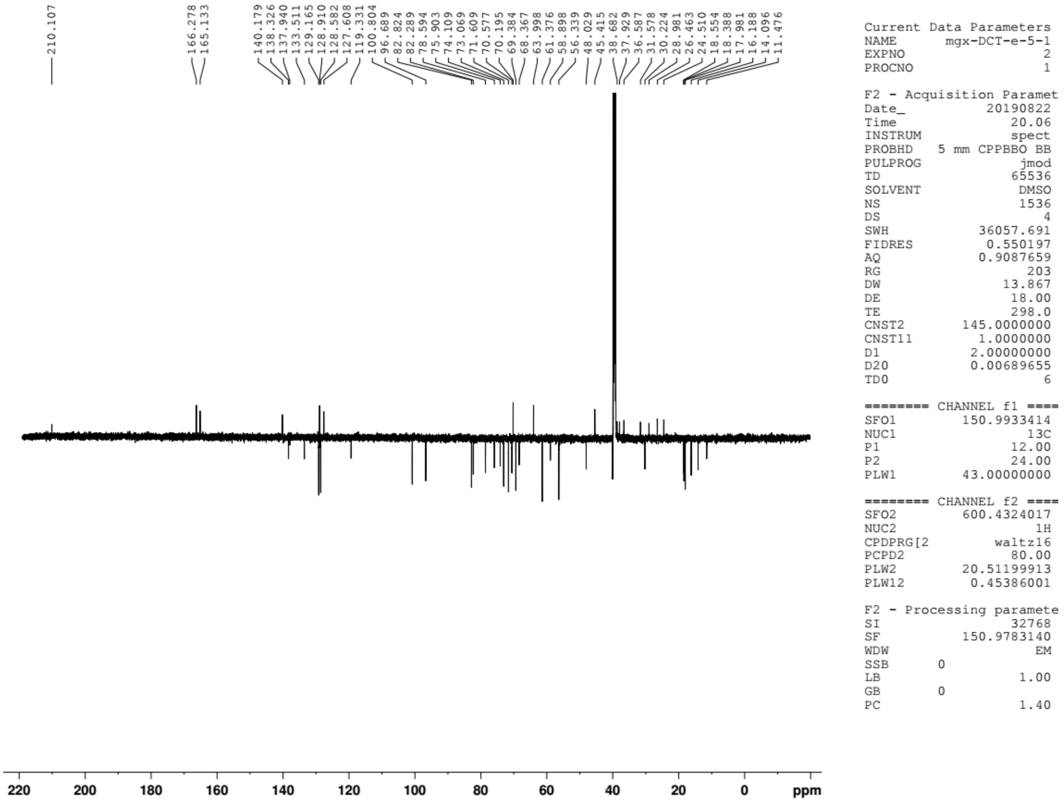

Figure S20. HSQC spectrum of the new compound **3**

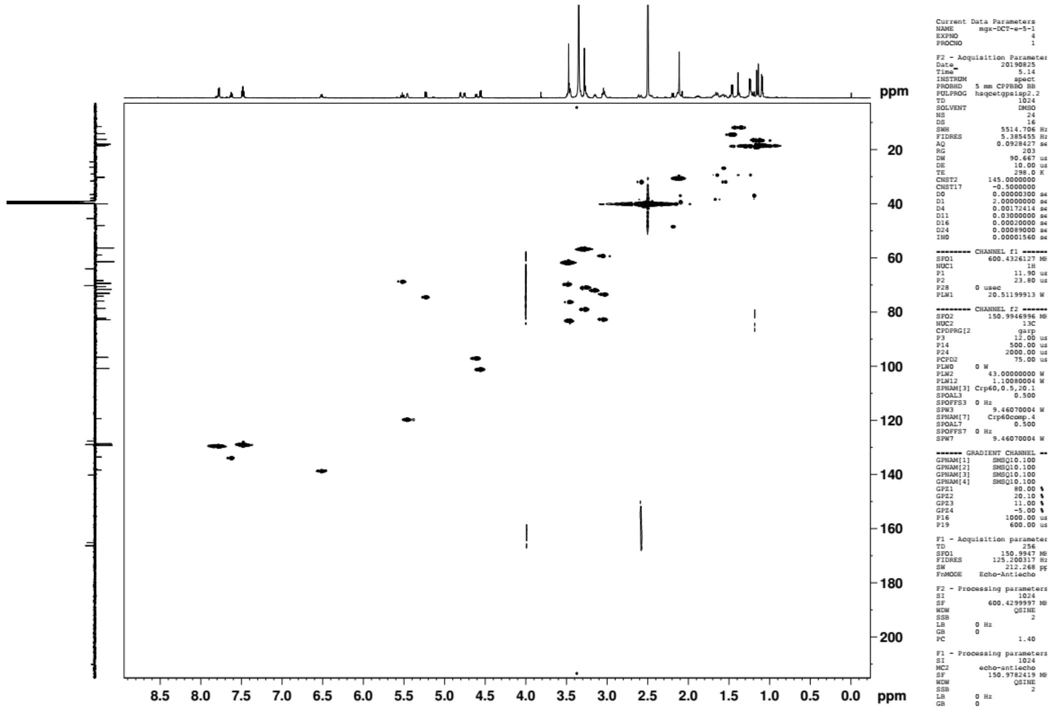

Figure S21. HMBC spectrum of the new compound **3**

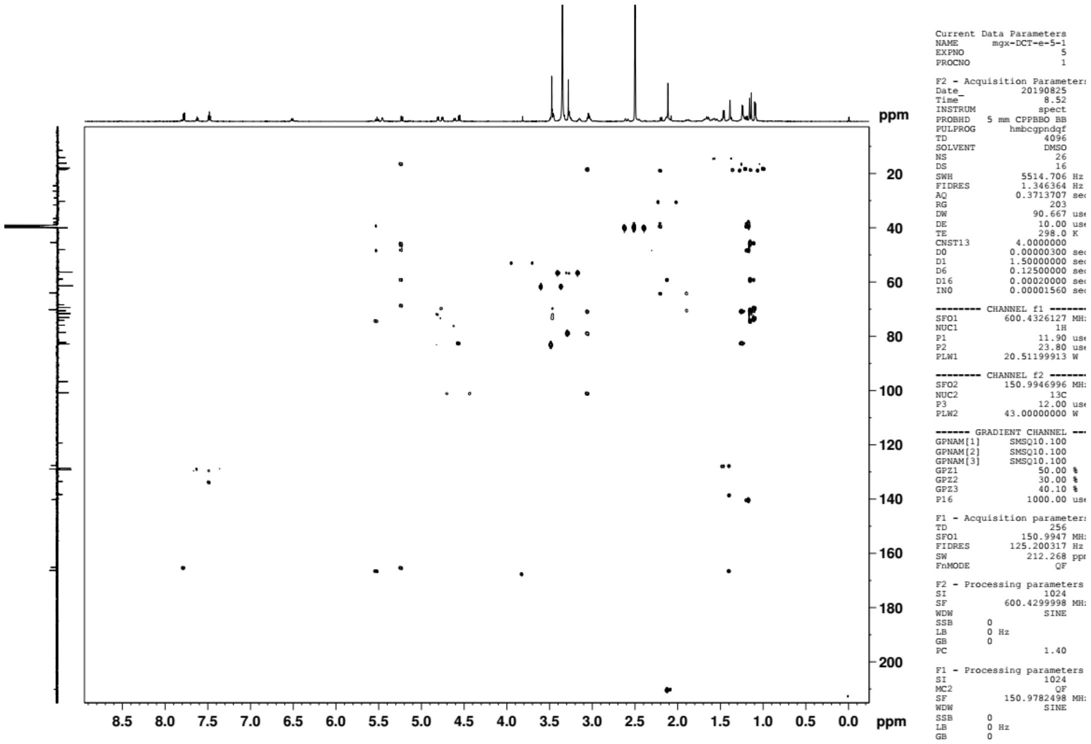

Figure S22. ^{1}H-^{1}H COSY spectrum of the new compound **3**

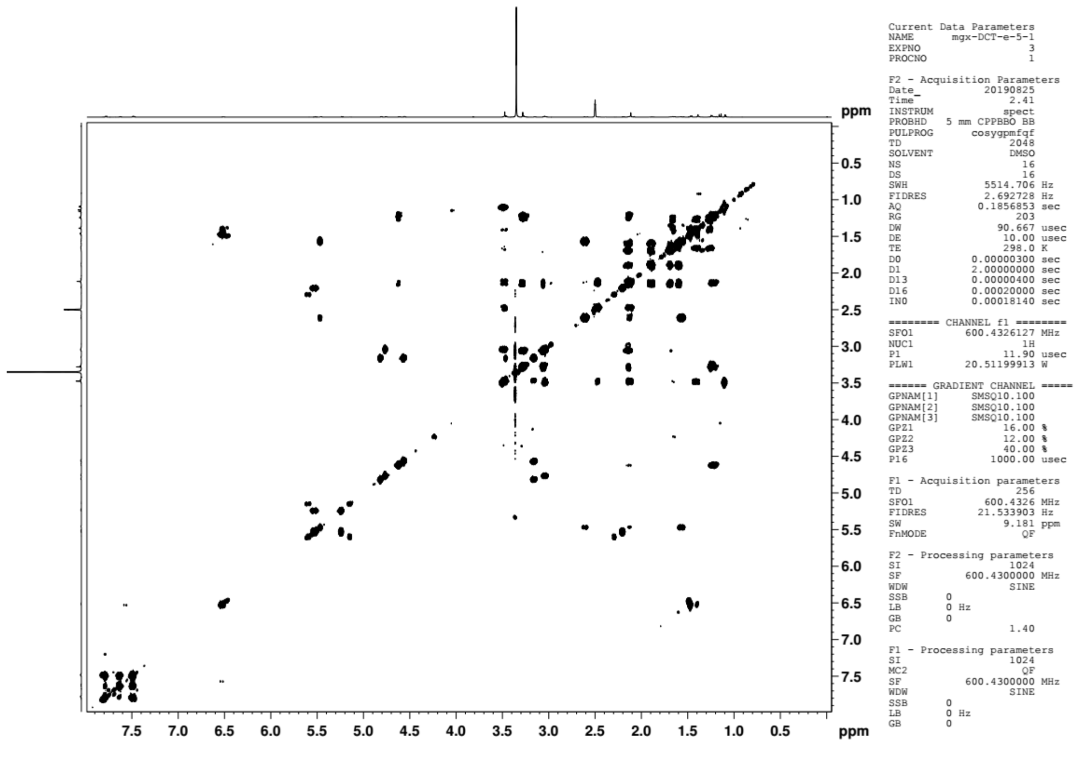

Figure S23. NOESY spectrum of the new compound **3**

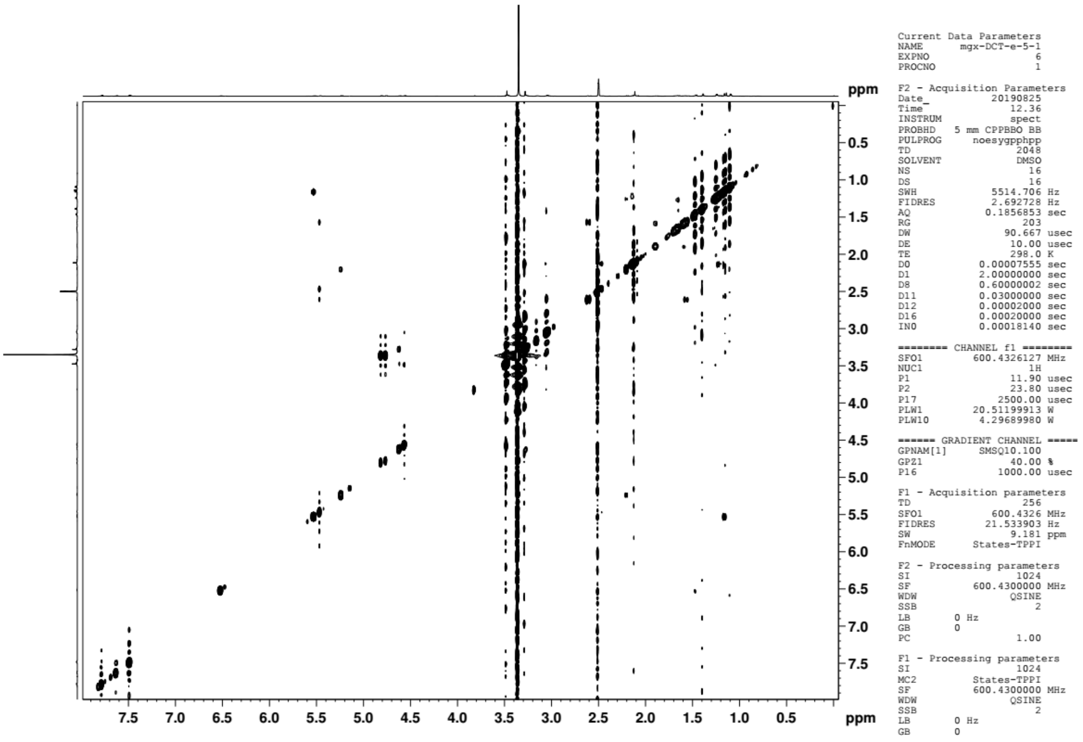

Figure S24. IR spectrum of the new compound **3**

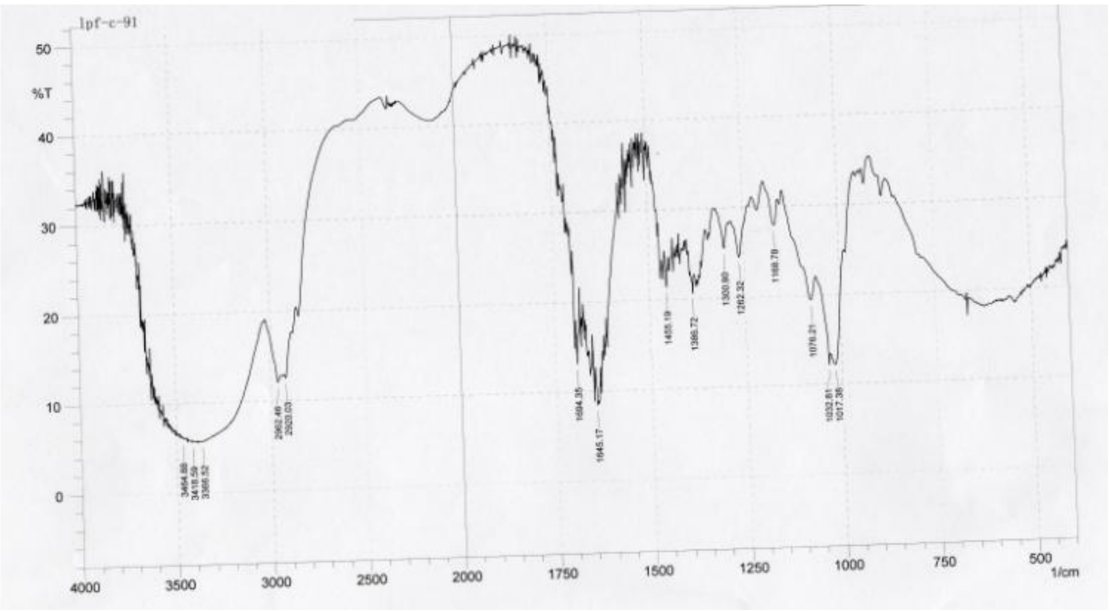

Figure S25. HRESIMS spectrum of the new compound **3**

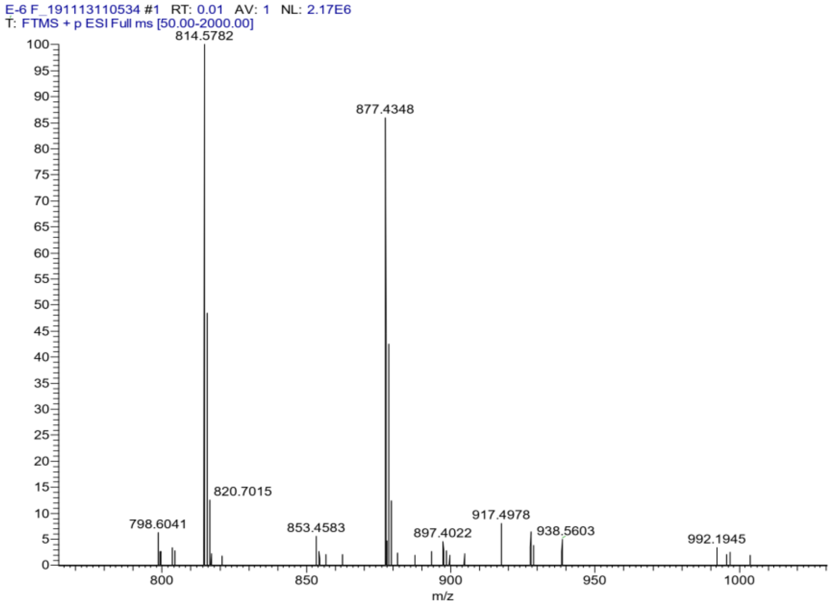

Figure S26. Key HMBC (Arrows) and ^{1}H-^{1}H COSY (Bonds) correlations of the new compound **3**

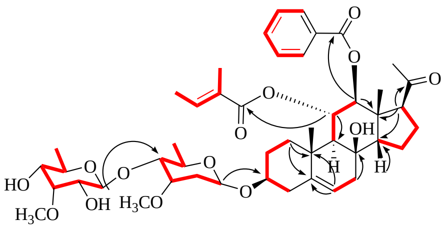

Figure S27. ^{1}H-NMR (600 MHz, DMSO) spectrum of the new compound **4**

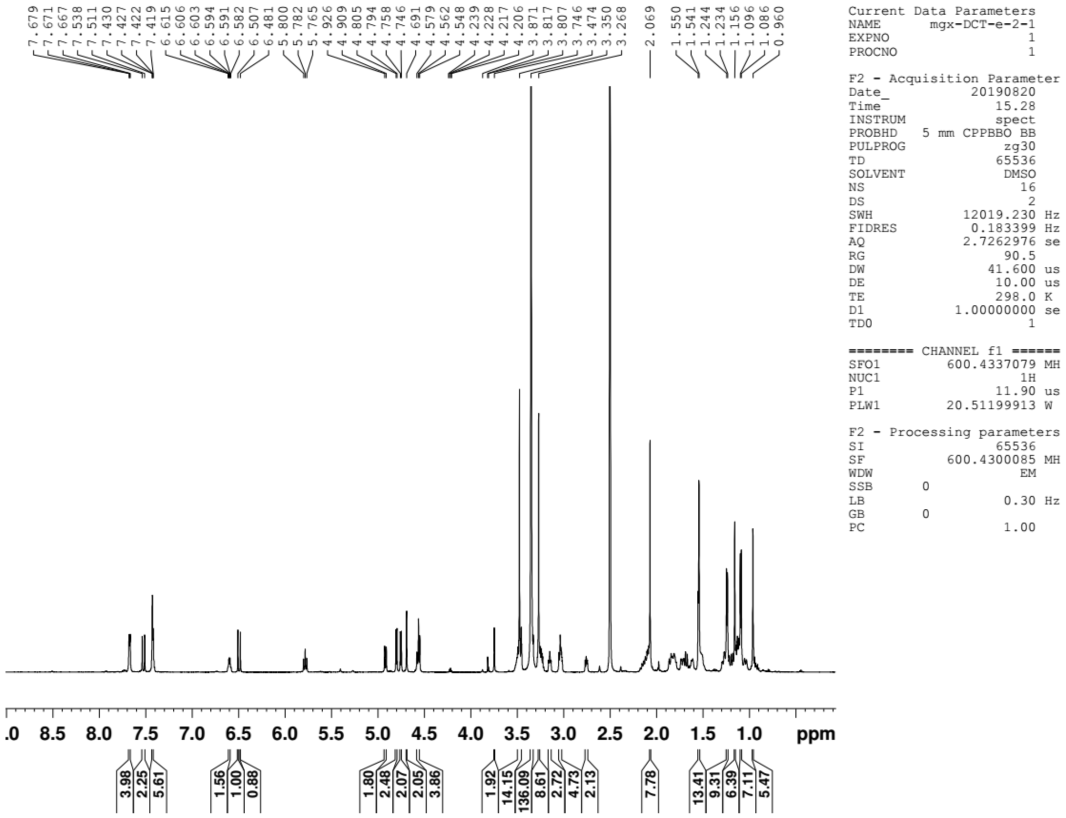

Figure S28. ^{13}C-APT (150 MHz, DMSO) spectrum of the new compound **4**

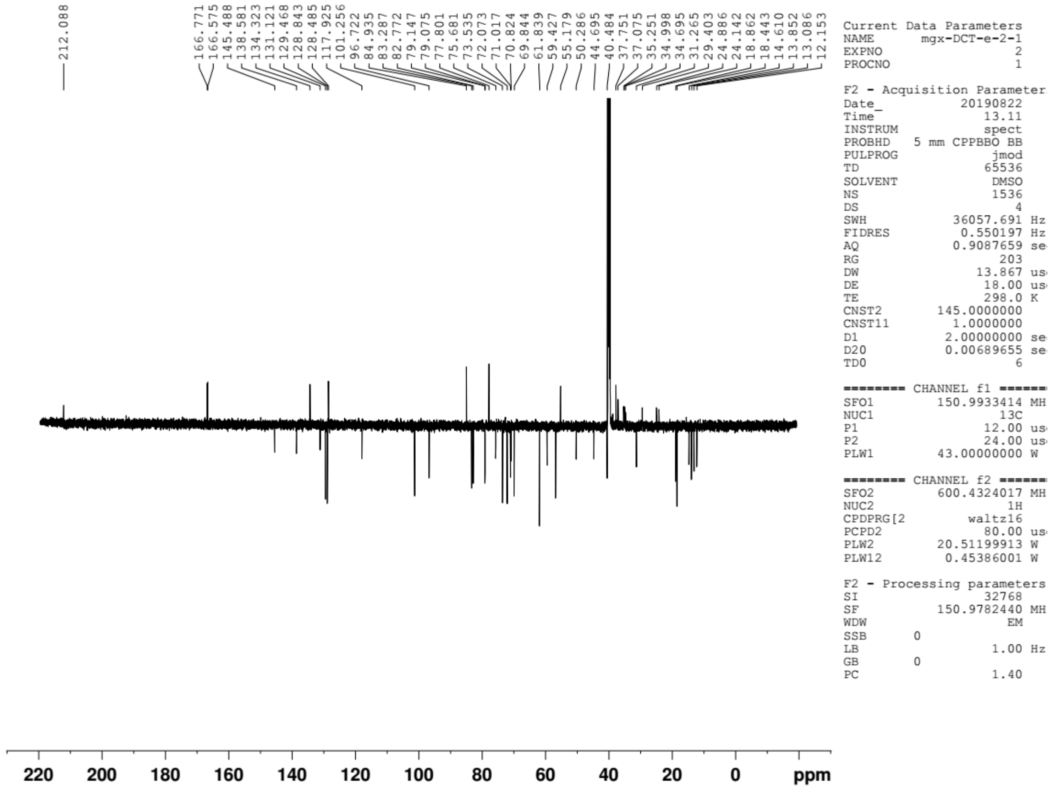

Figure S29. HSQC spectrum of the new compound **4**

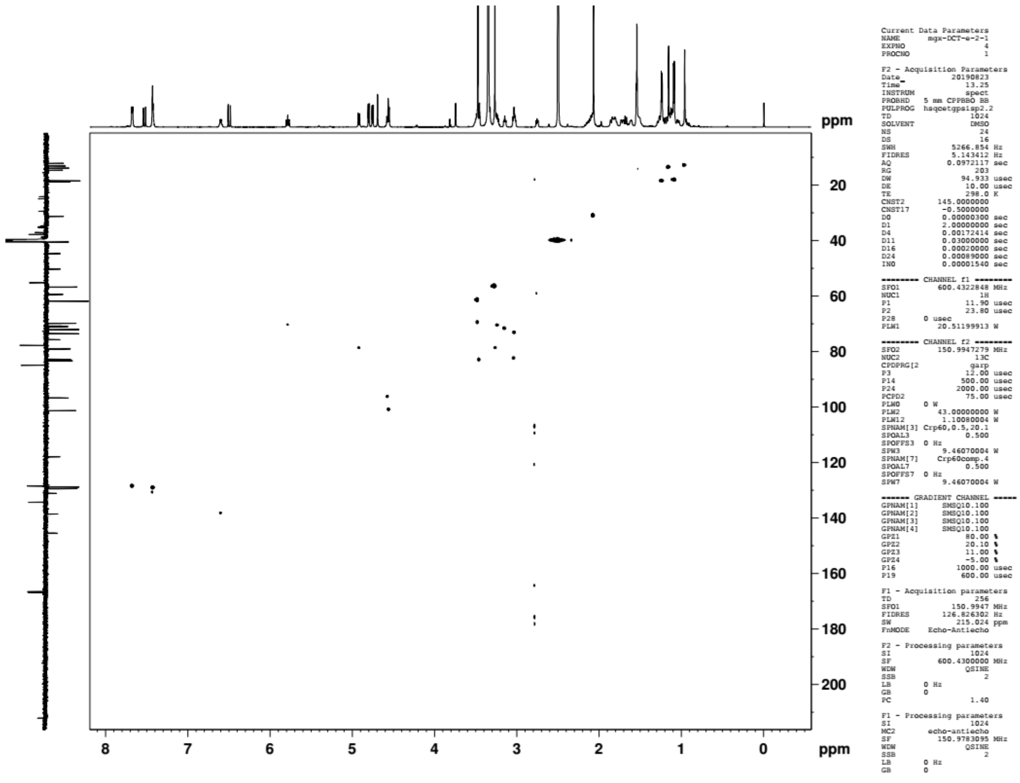

Figure S30. HMBC spectrum of the new compound **4**

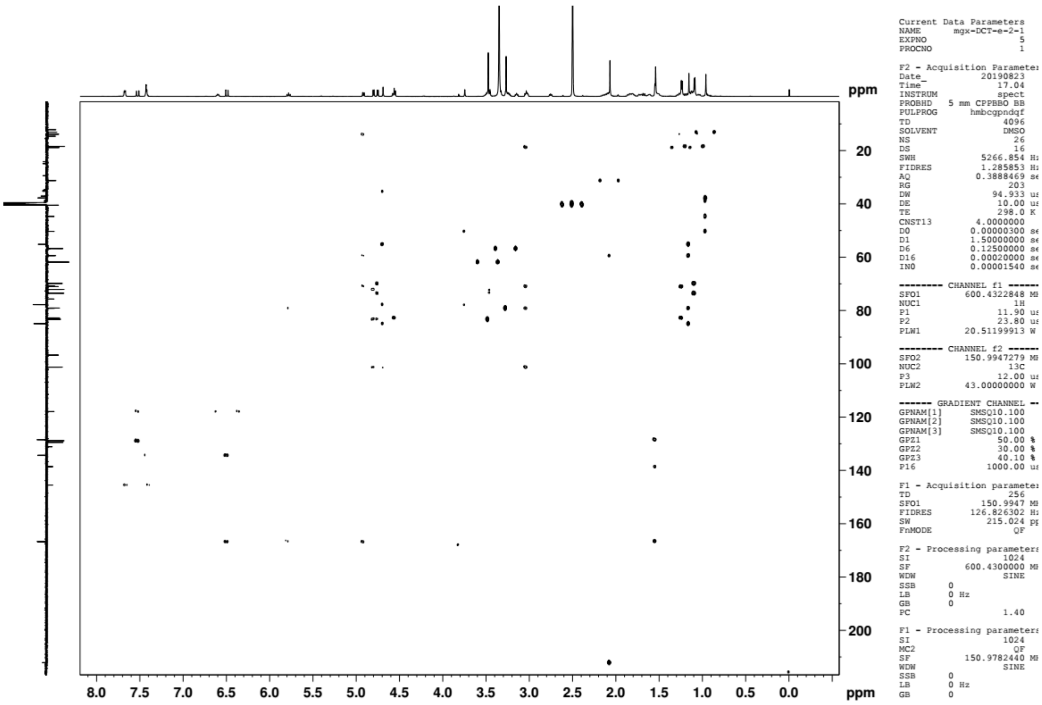

Figure S31. ^{1}H-^{1}H COSY spectrum of the new compound **4**

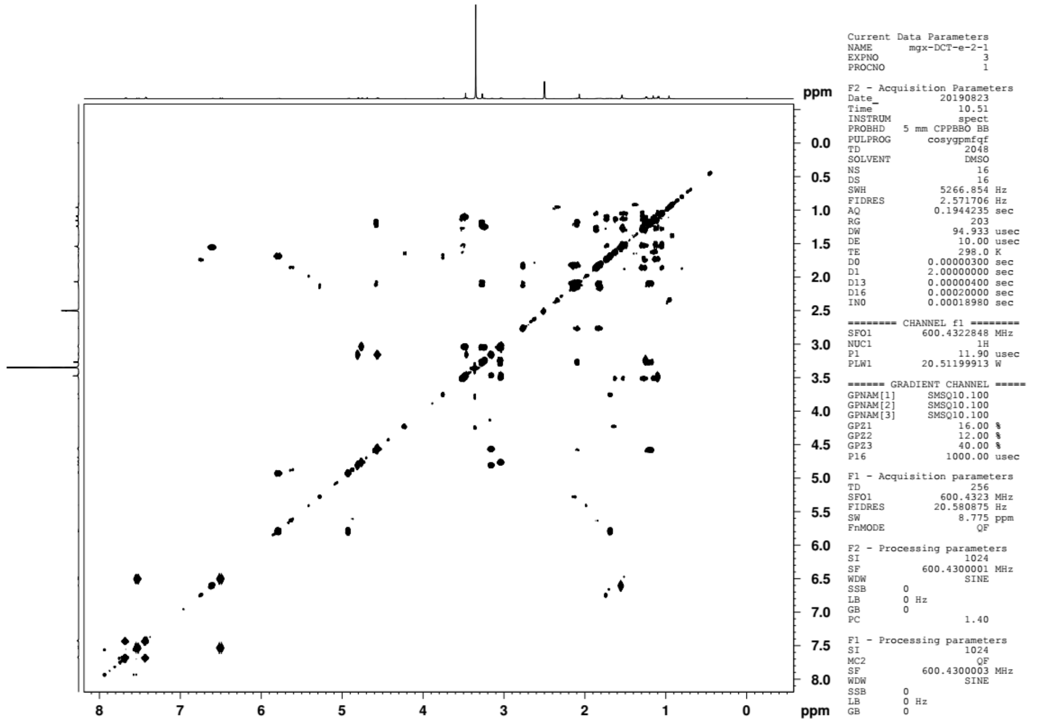

Figure S32. NOESY spectrum of the new compound **4**

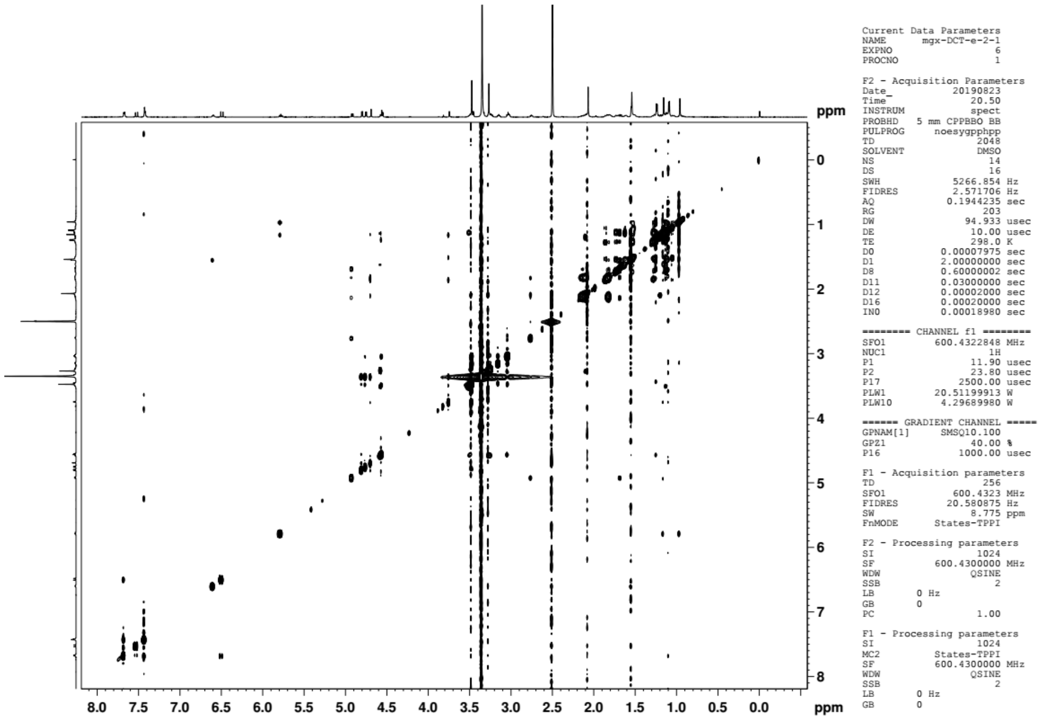

Figure S33. IR spectrum of the new compound **4**

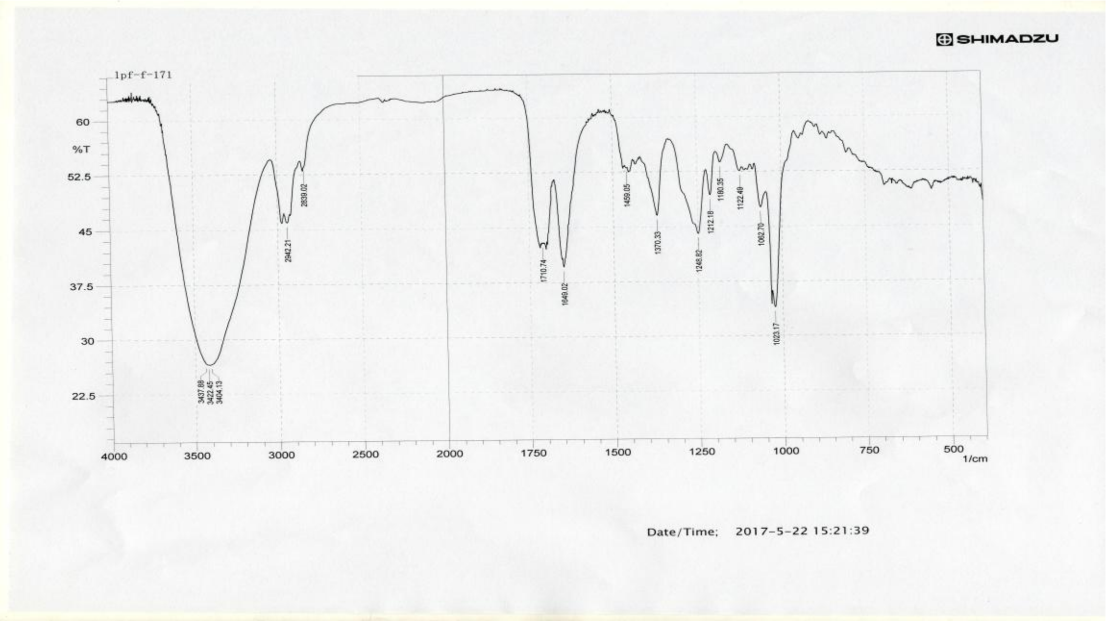

Figure S34. HRESIMS spectrum of the new compound **4**

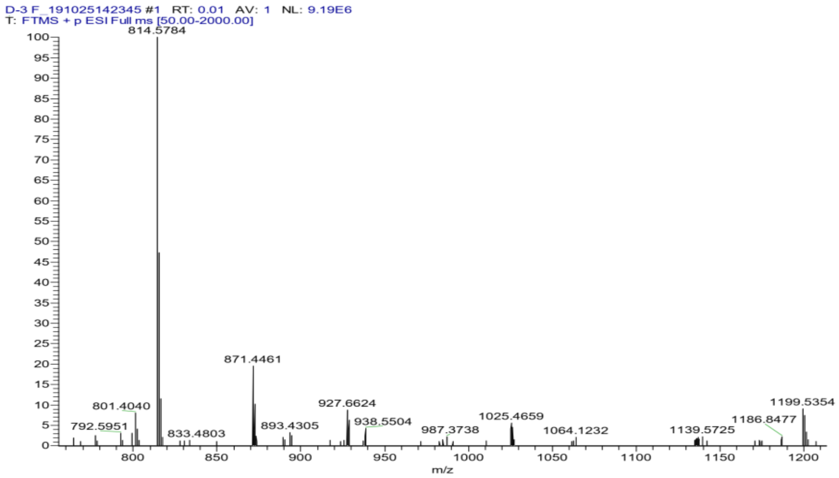

Figure S35. Key HMBC (Arrows) and ^{1}H-^{1}H COSY (Bonds) correlations of the new compound **4**

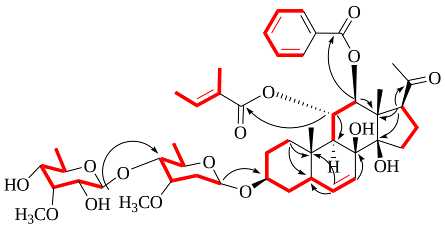

Figure S36. ^{1}H-NMR (600 MHz, DMSO) spectrum of the new compound **5**

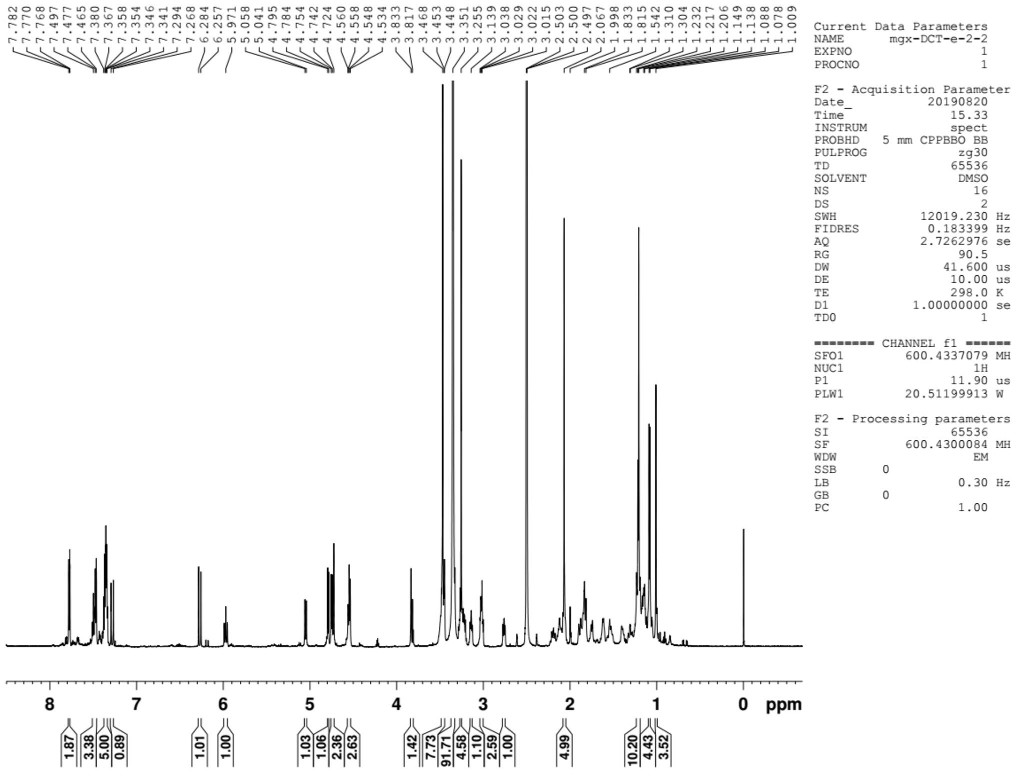

Figure S37. ^{13}C-APT (150 MHz, DMSO) spectrum of the new compound **5**

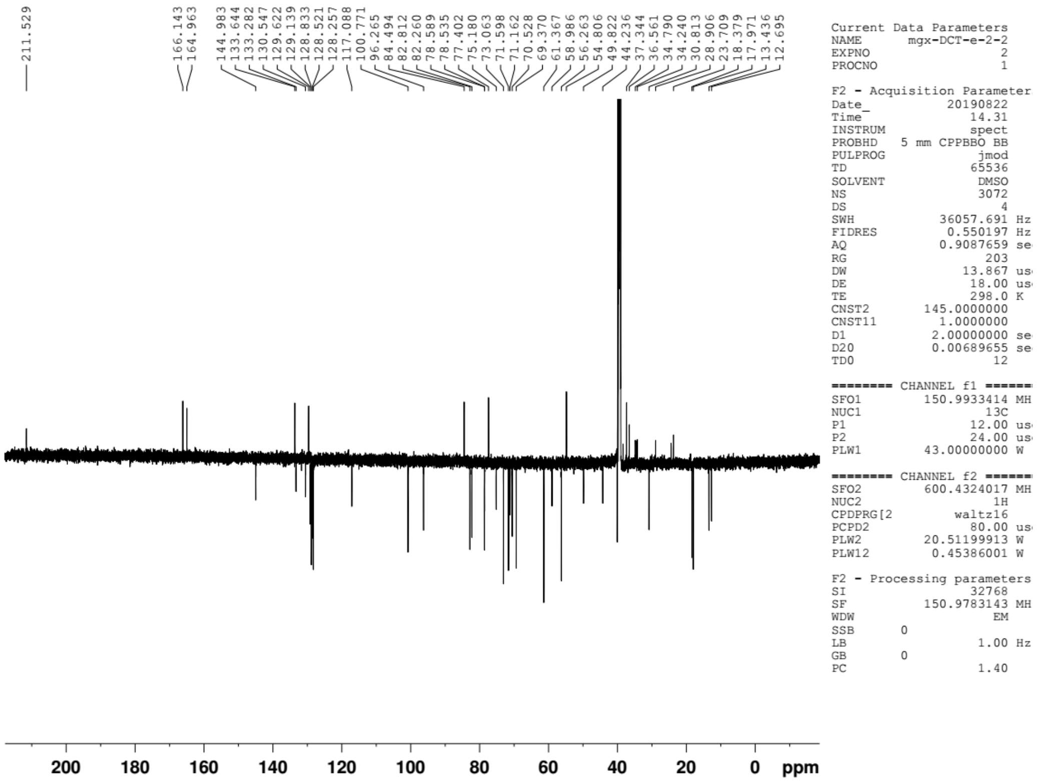

Figure S38. HSQC spectrum of the new compound **5**

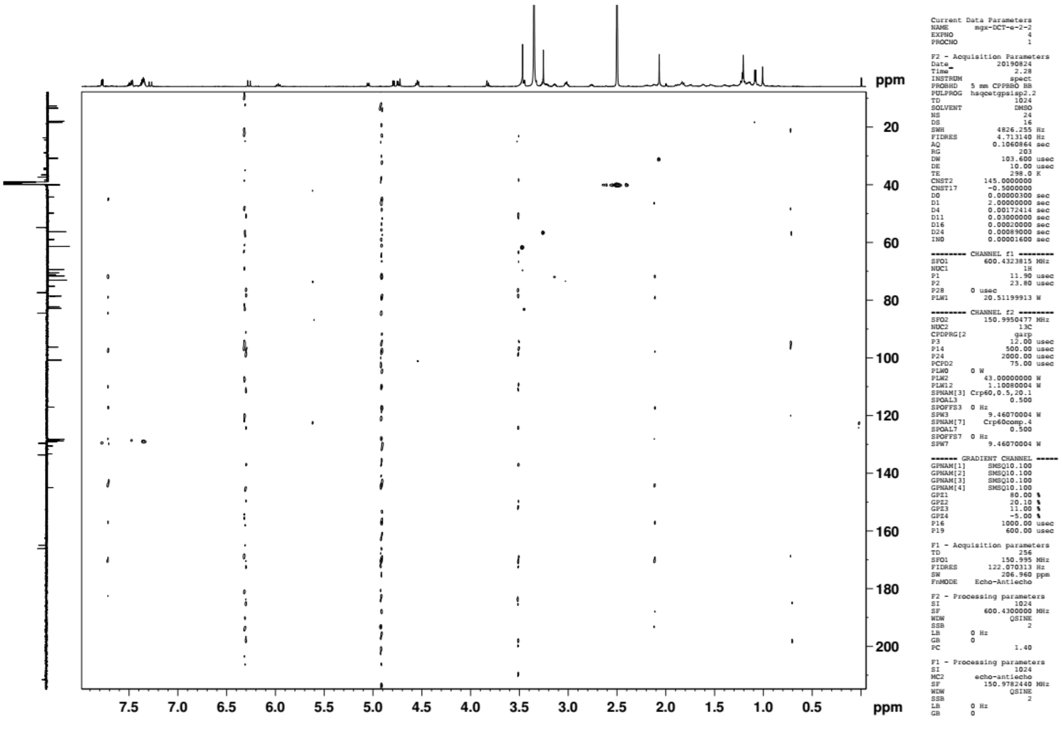

Figure S39. HMBC spectrum of the new compound **5**

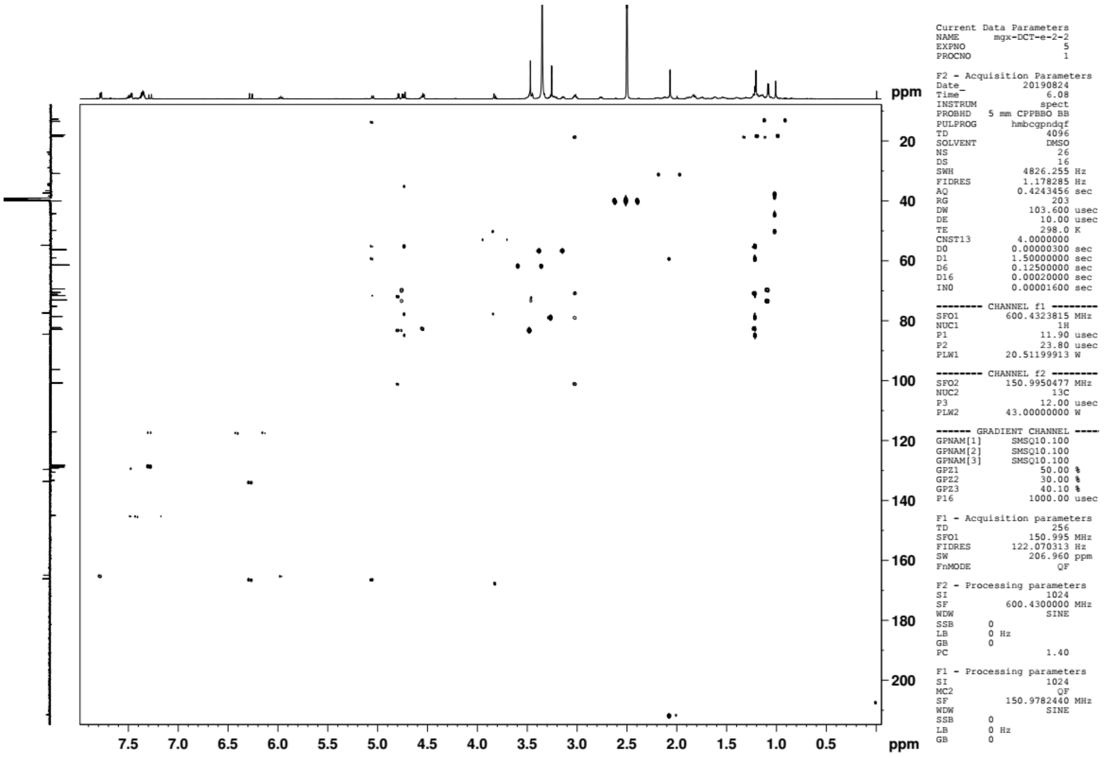

Figure S40. ^{1}H-^{1}H COSY spectrum of the new compound **5**

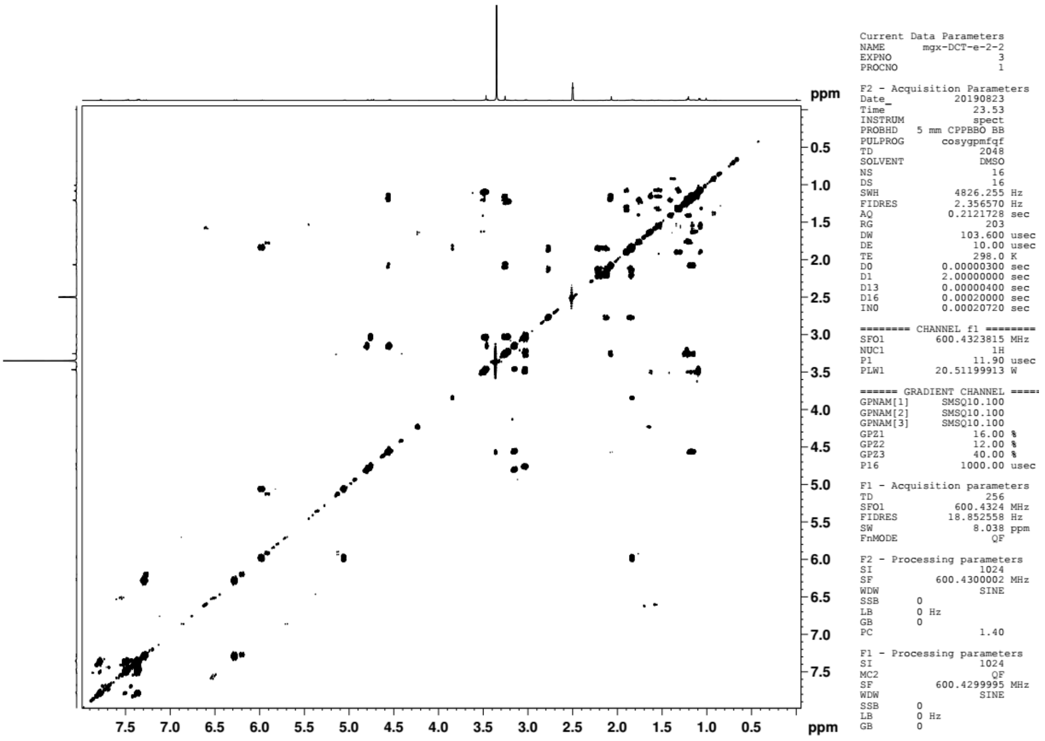

Figure S41. NOESY spectrum of the new compound **5**

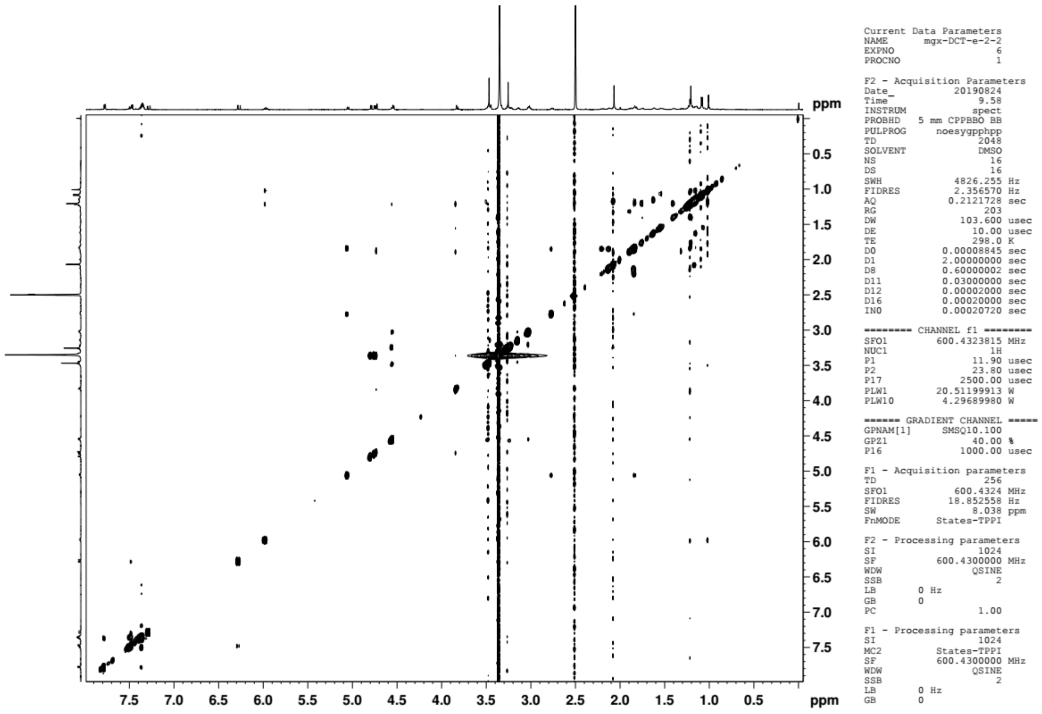

Figure S42. IR spectrum of the new compound **5**

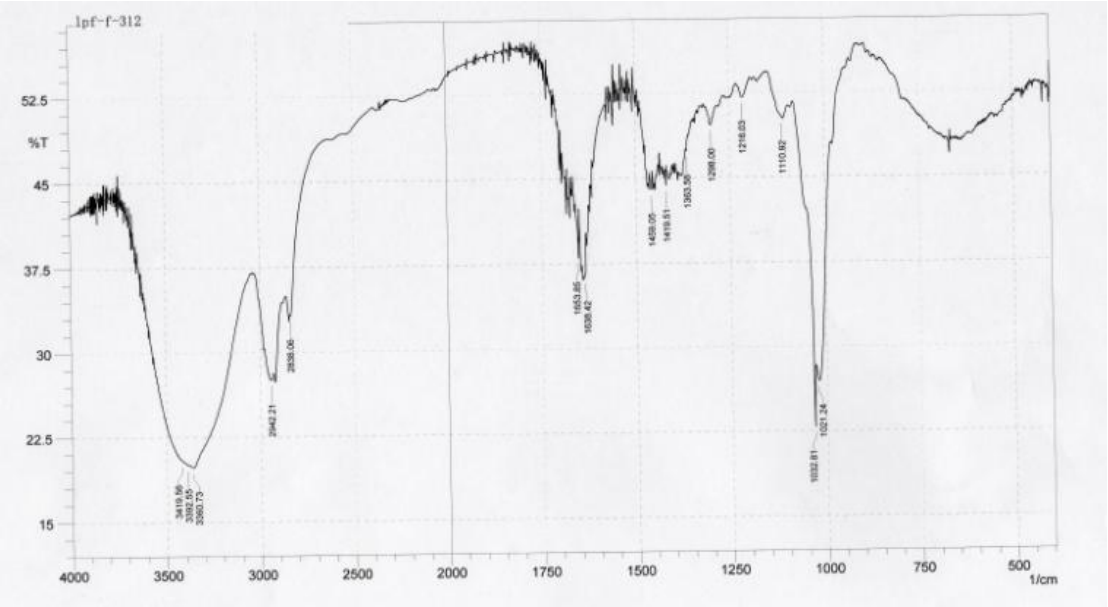

Figure S43. HRESIMS spectrum of the new compound **5**

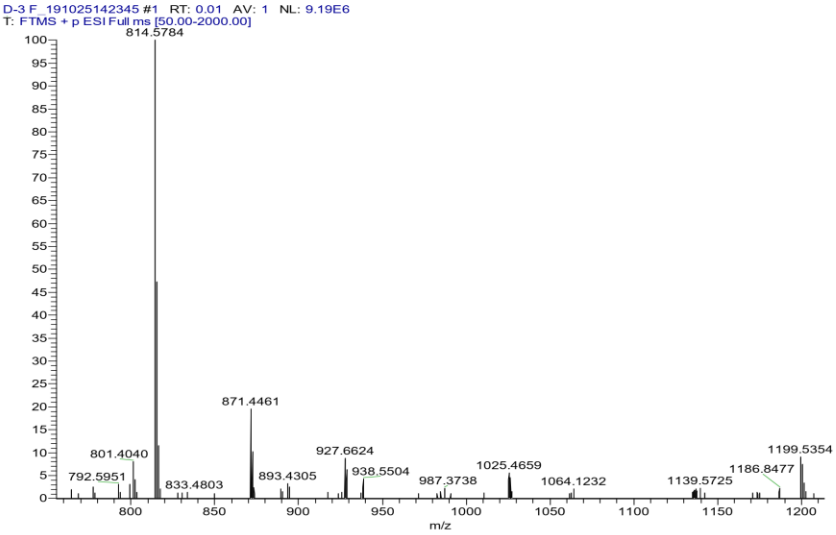

Figure S44. Key HMBC (Arrows) and ^{1}H-^{1}H COSY (Bonds) correlations of the new compound **5**

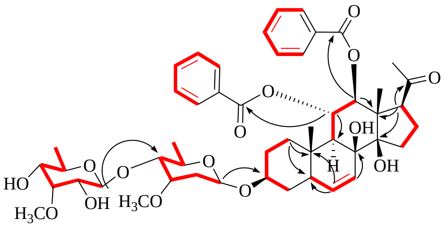

Figure S45. ^{1}H-NMR (600 MHz, DMSO) spectrum of the new compound **6**

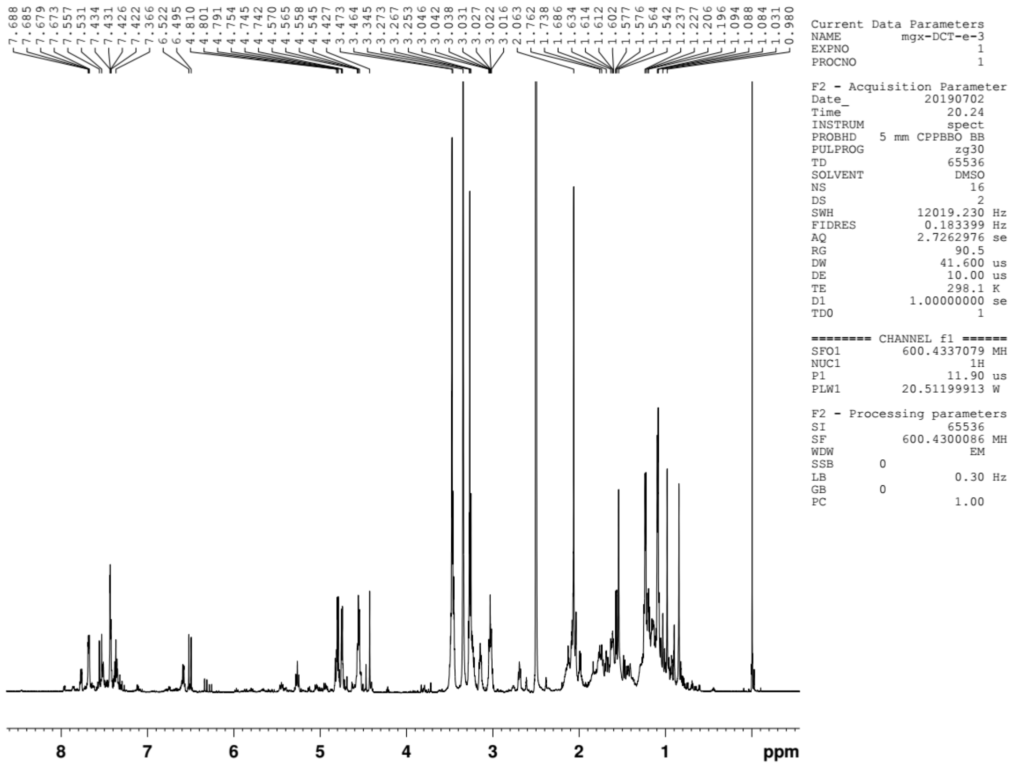

Figure S46. ^{13}C-APT (150 MHz, DMSO) spectrum of the new compound **6**

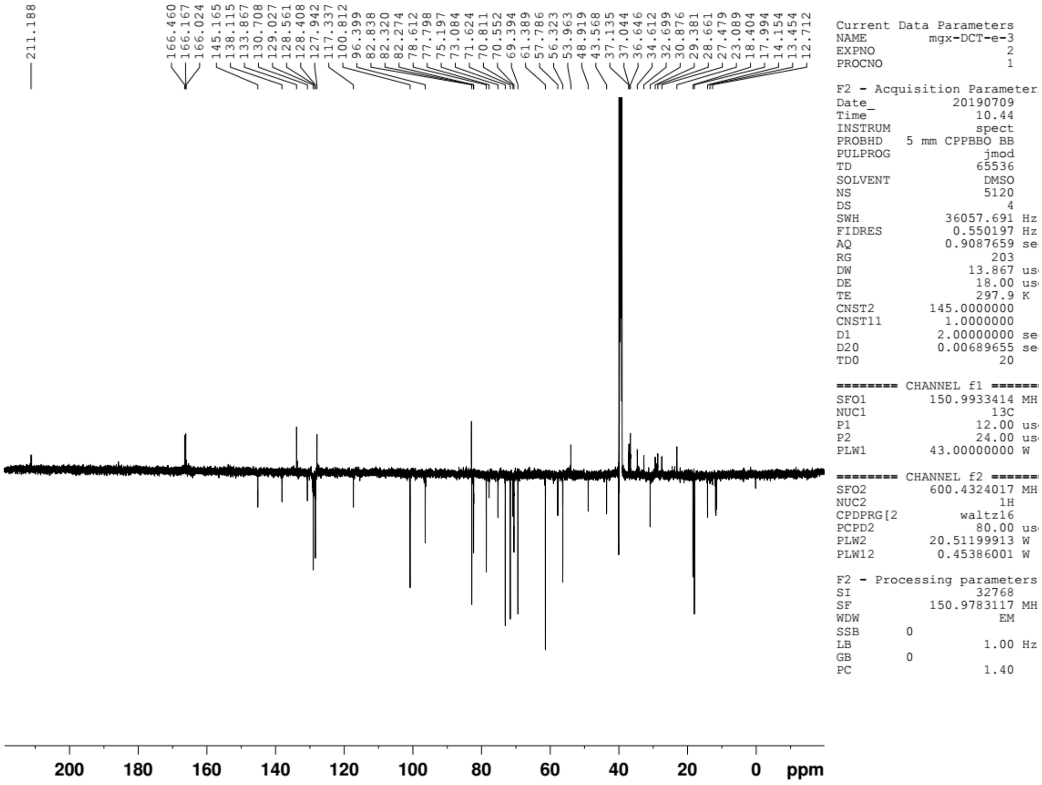

Figure S47. HSQC spectrum of the new compound **6**

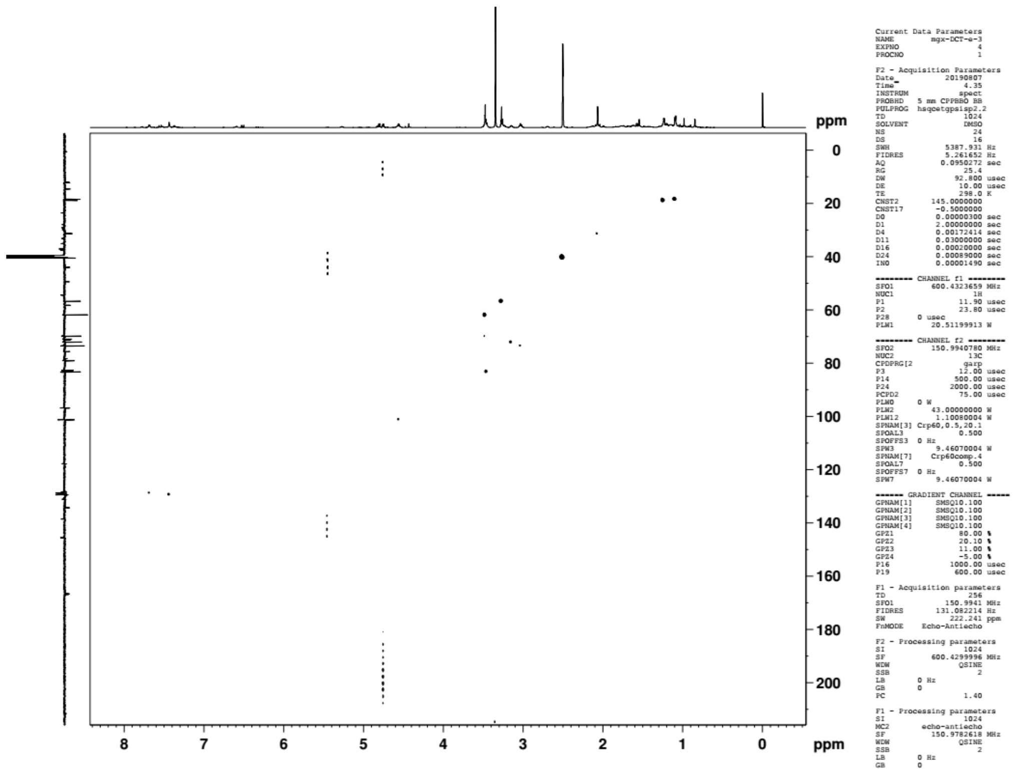

Figure S48. HMBC spectrum of the new compound **6**

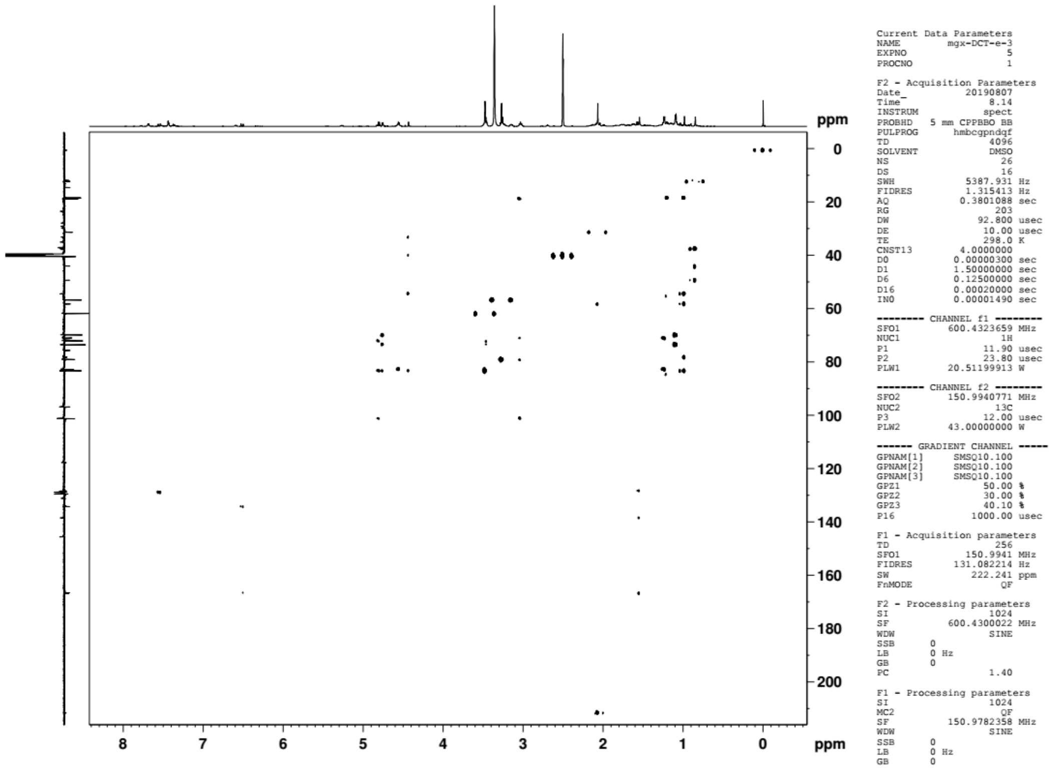

Figure S49. ^{1}H-^{1}H COSY spectrum of the new compound **6**

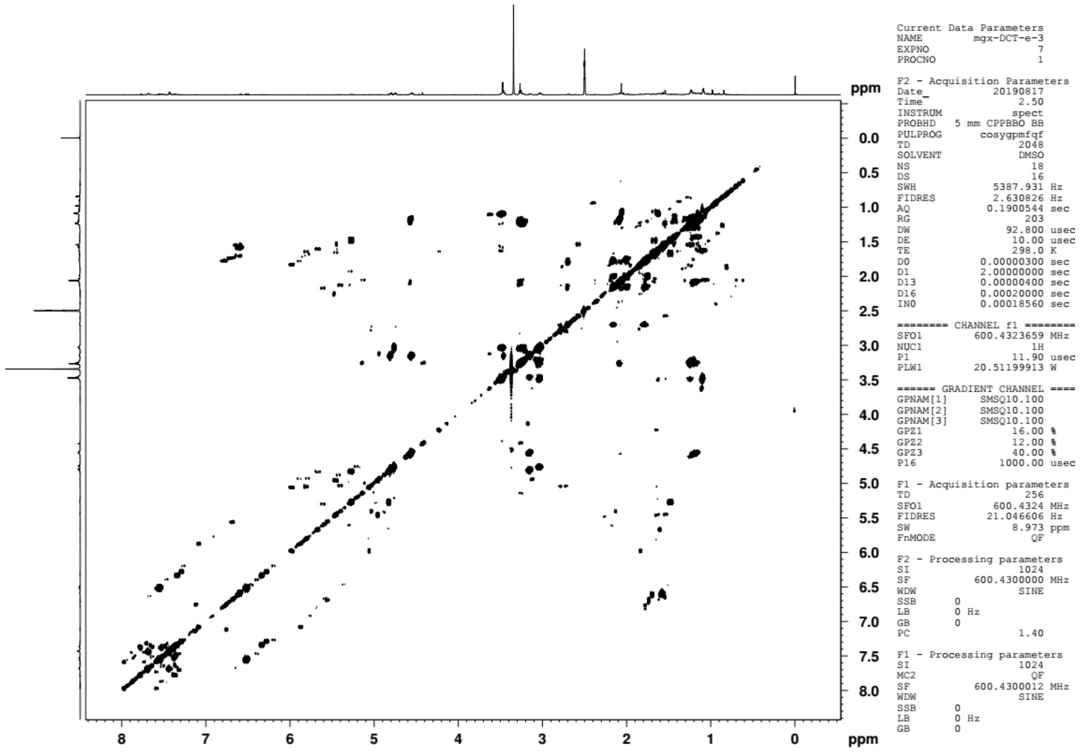

Figure S50. NOESY spectrum of the new compound **6**

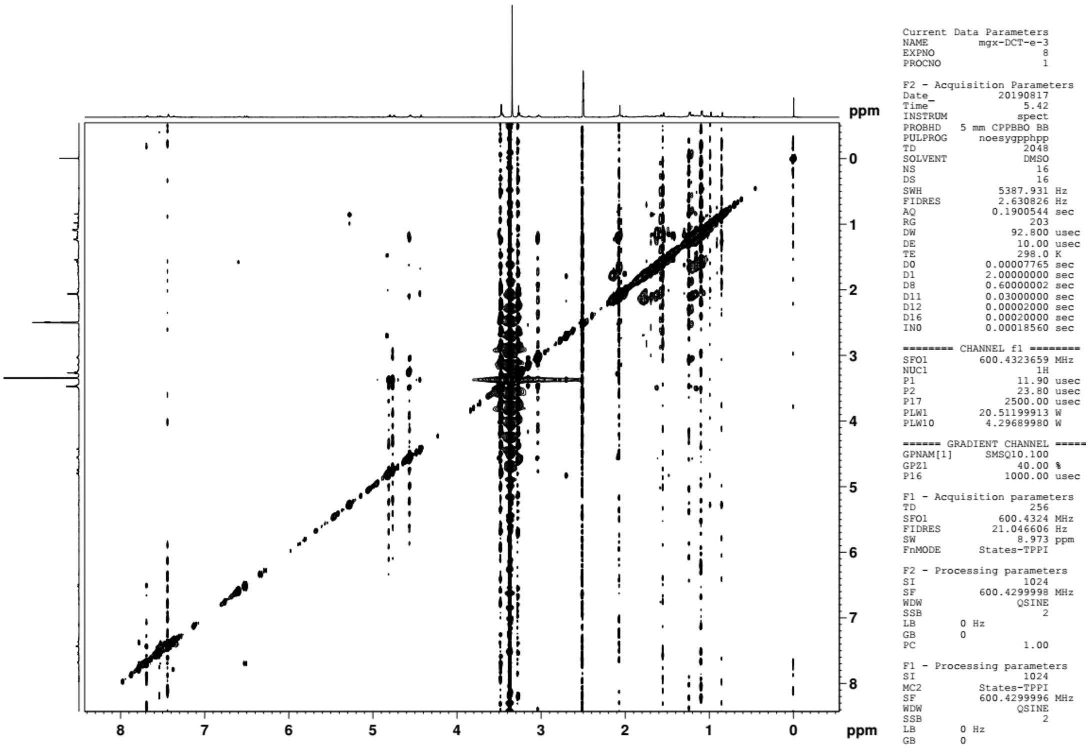

Figure S51. IR spectrum of the new compound **6**

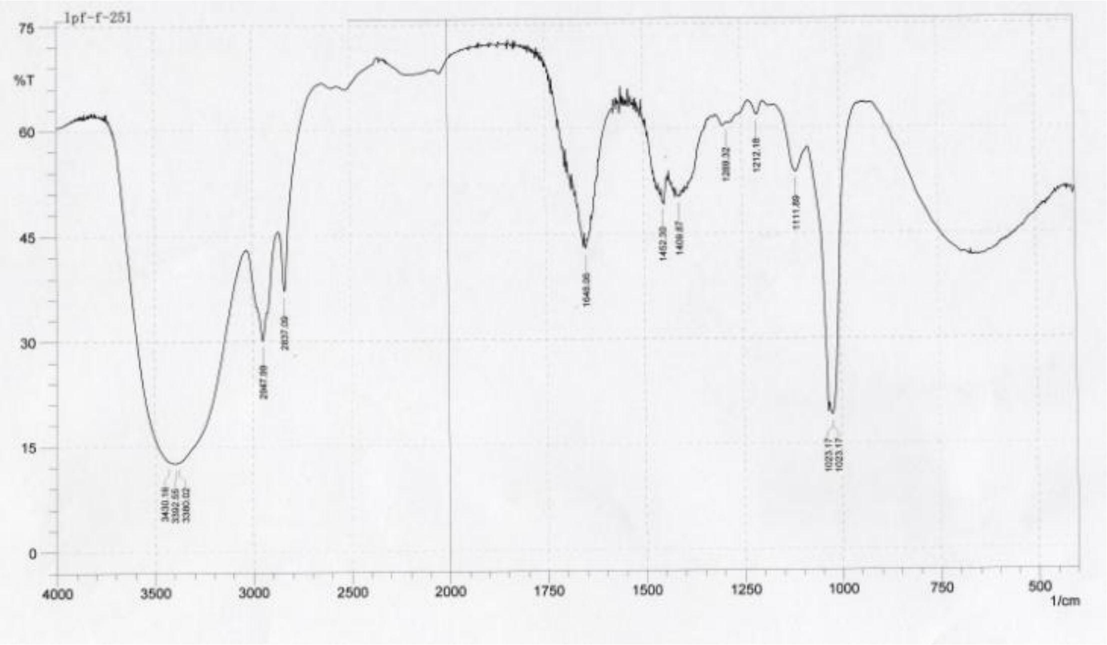

Figure S52. HRESIMS spectrum of the new compound **6**

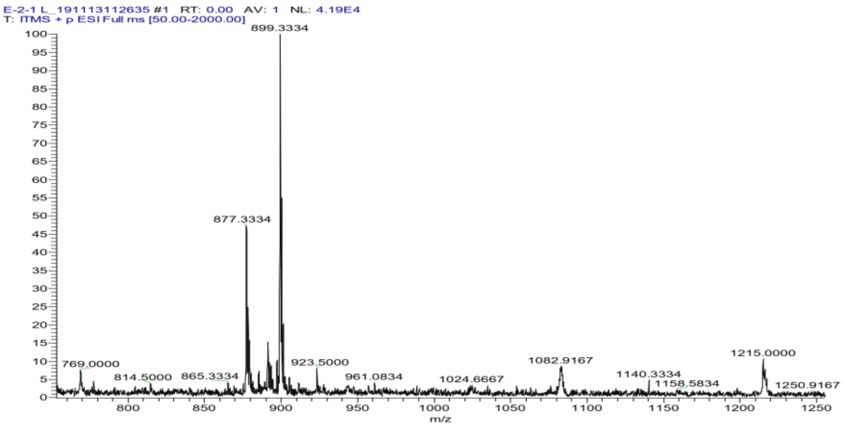

Figure S53. Key HMBC (Arrows) and ^{1}H-^{1}H COSY (Bonds) correlations of the new compound **6**

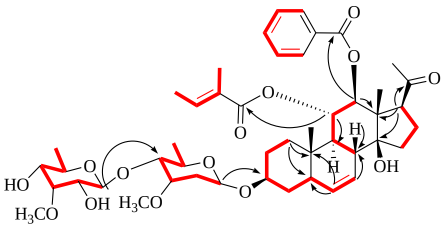

Figure S54. ^{1}H-NMR (600 MHz, DMSO) spectrum of the new compound **7**

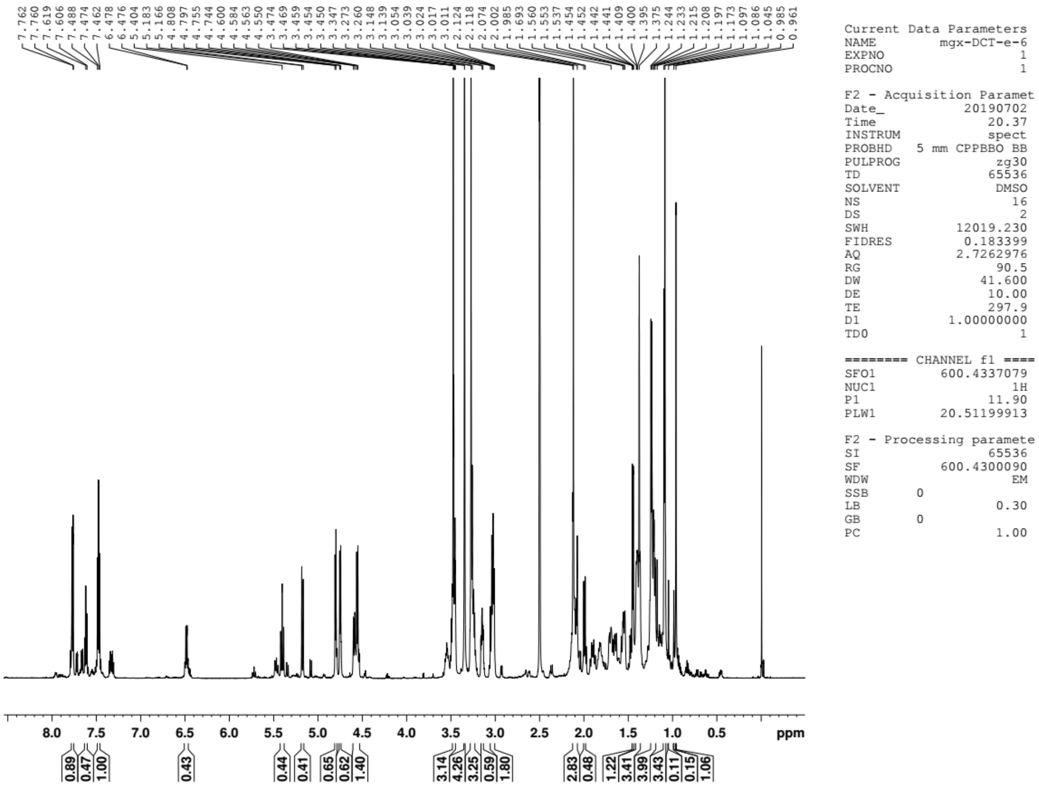

Figure S55. ^{13}C-APT (150 MHz, DMSO) spectrum of the new compound **7**

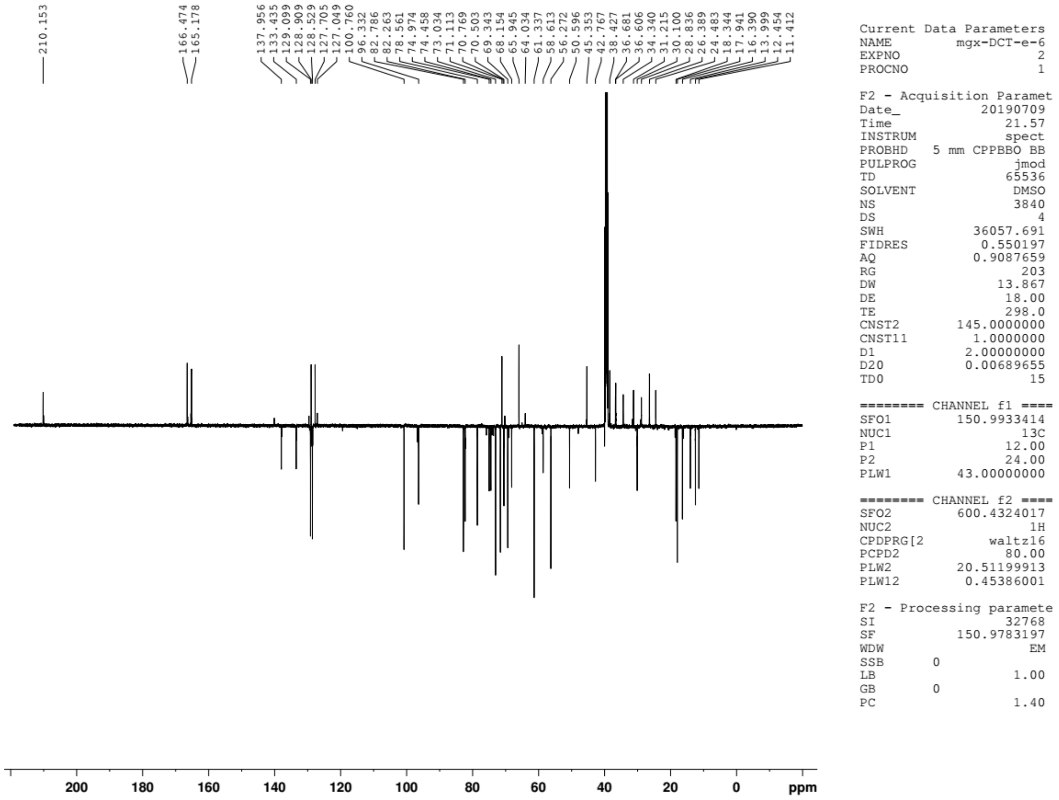

Figure S56. HSQC spectrum of the new compound **7**

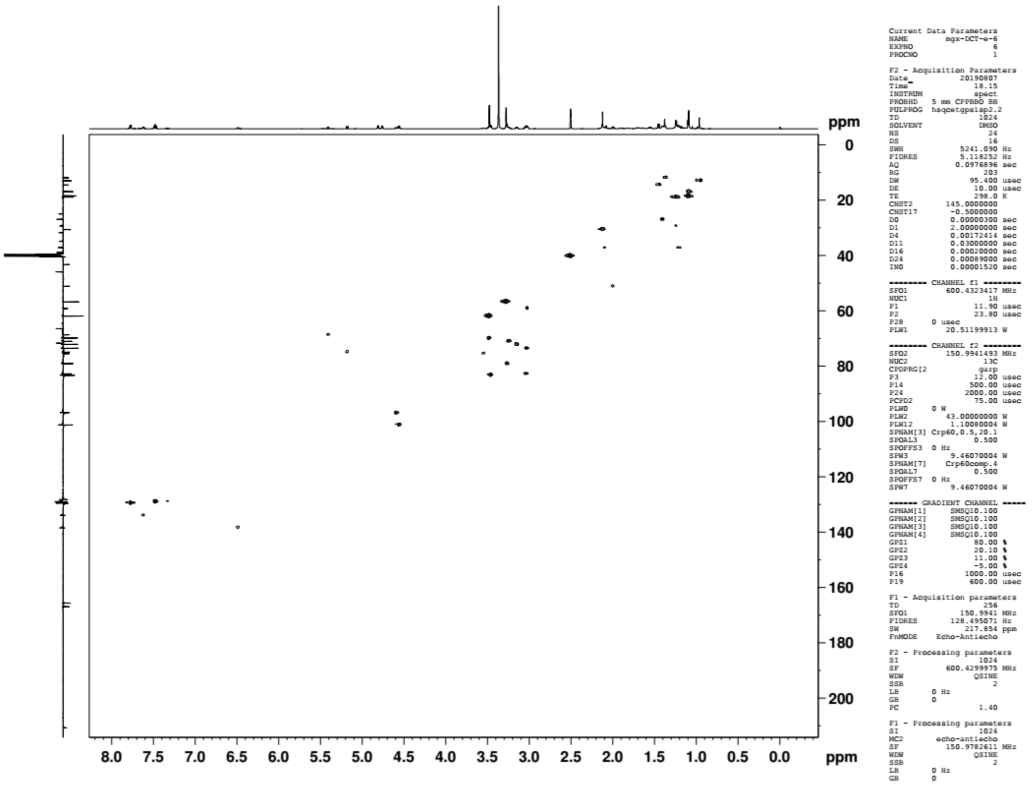

Figure S57. HMBC spectrum of the new compound **7**

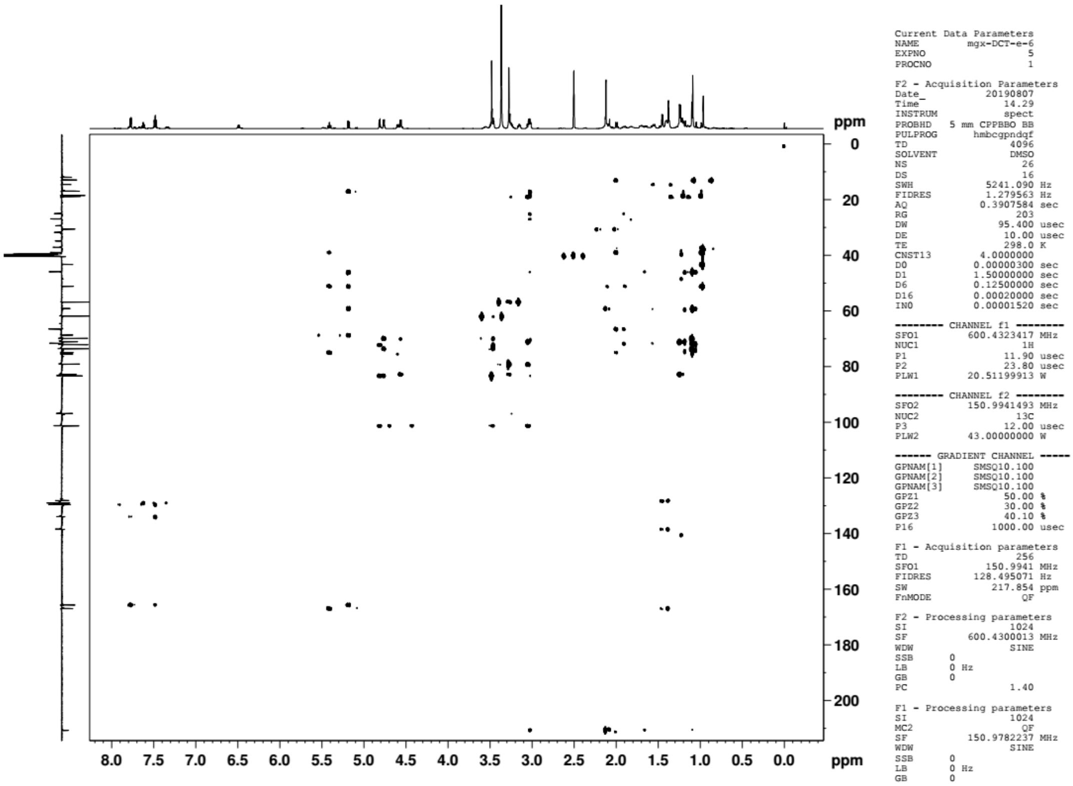

Figure S58. ^{1}H-^{1}H COSY spectrum of the new compound **7**

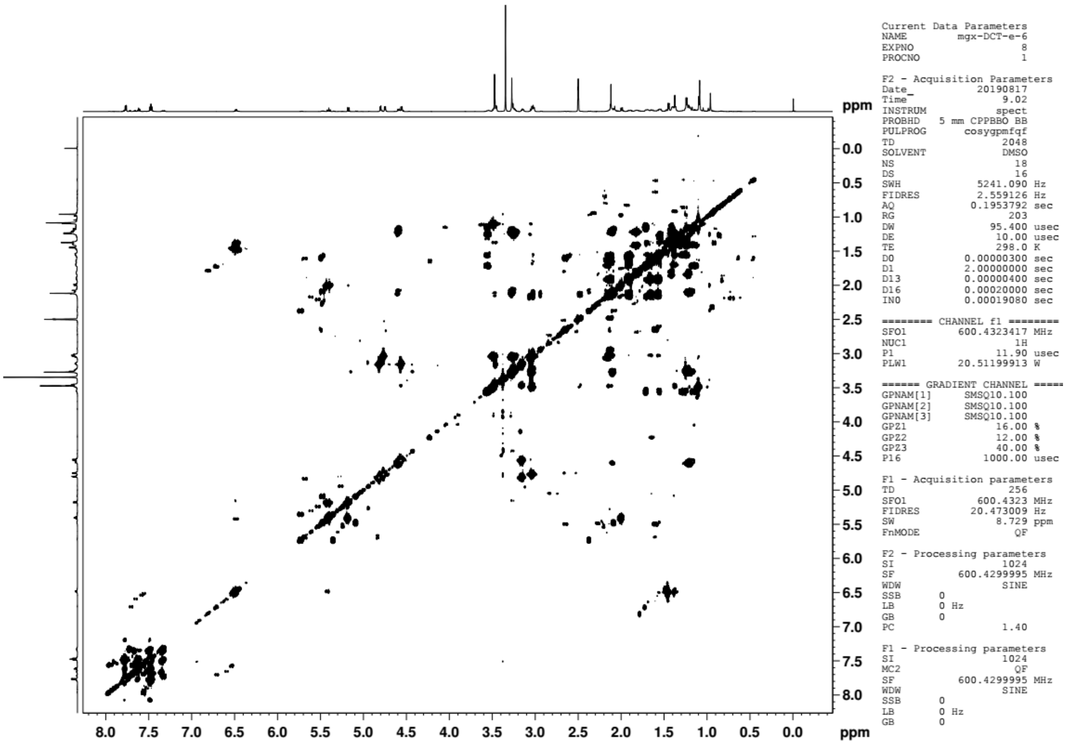

Figure S59. NOESY spectrum of the new compound **7**

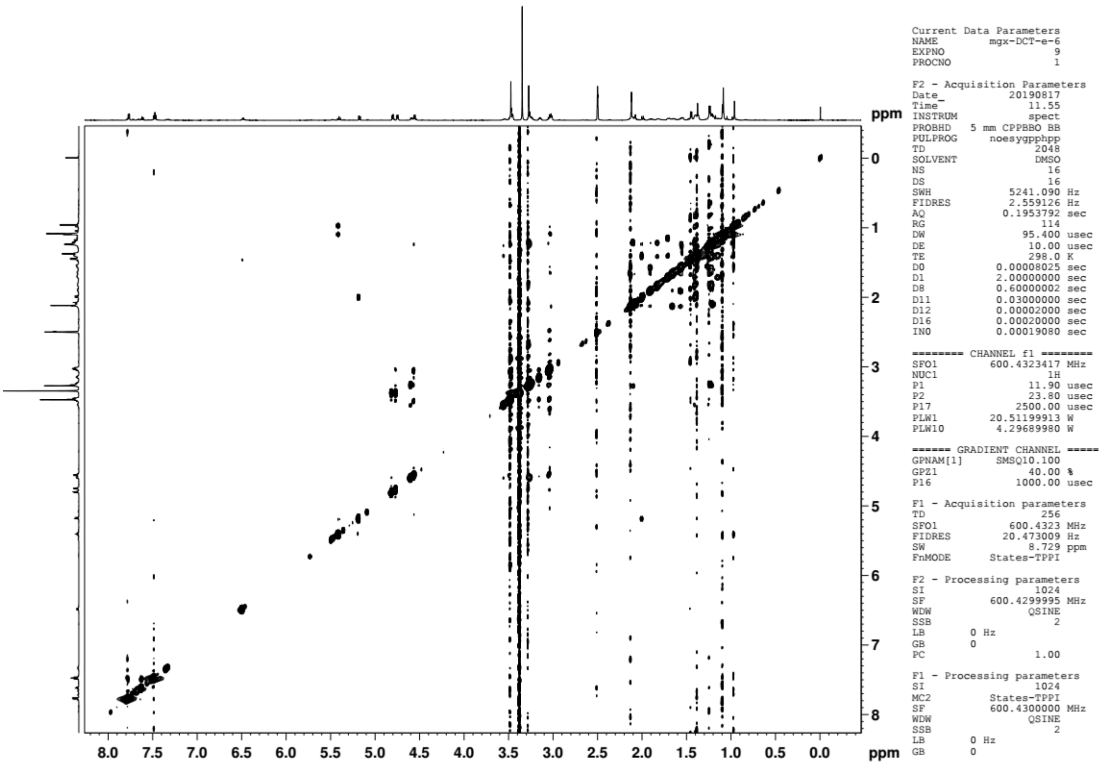

Figure S60. IR spectrum of the new compound **7**

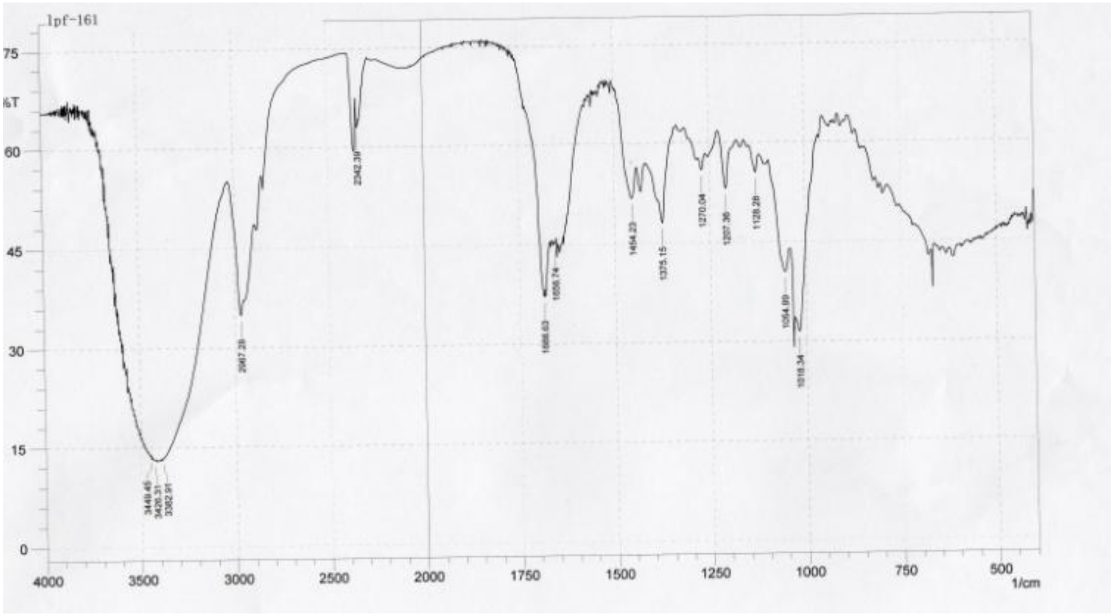

Figure S61. HRESIMS spectrum of the new compound **7**

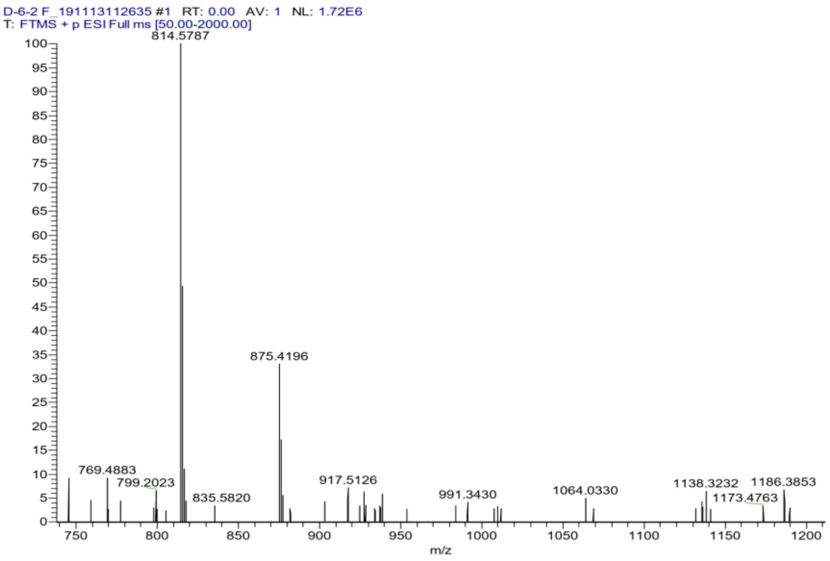

Figure S62. Key HMBC (Arrows) and ^{1}H-^{1}H COSY (Bonds) correlations of the new compound **7**

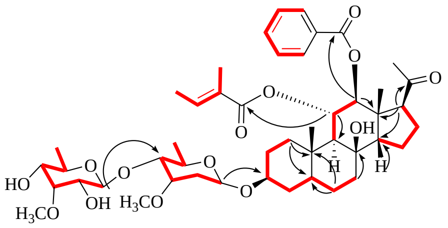

Figure S63. ^{1}H-NMR (600 MHz,DMSO) spectrum of the new compound **8**

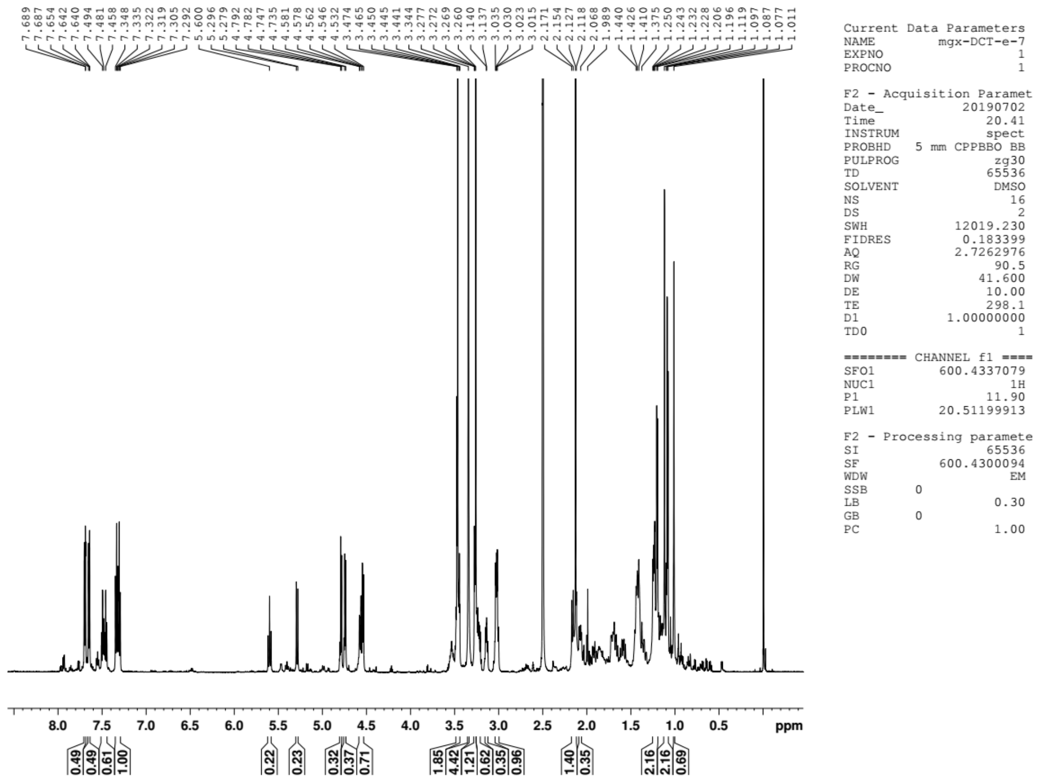

Figure S64. ^{13}C-APT (150 MHz, DMSO) spectrum of the new compound **8**

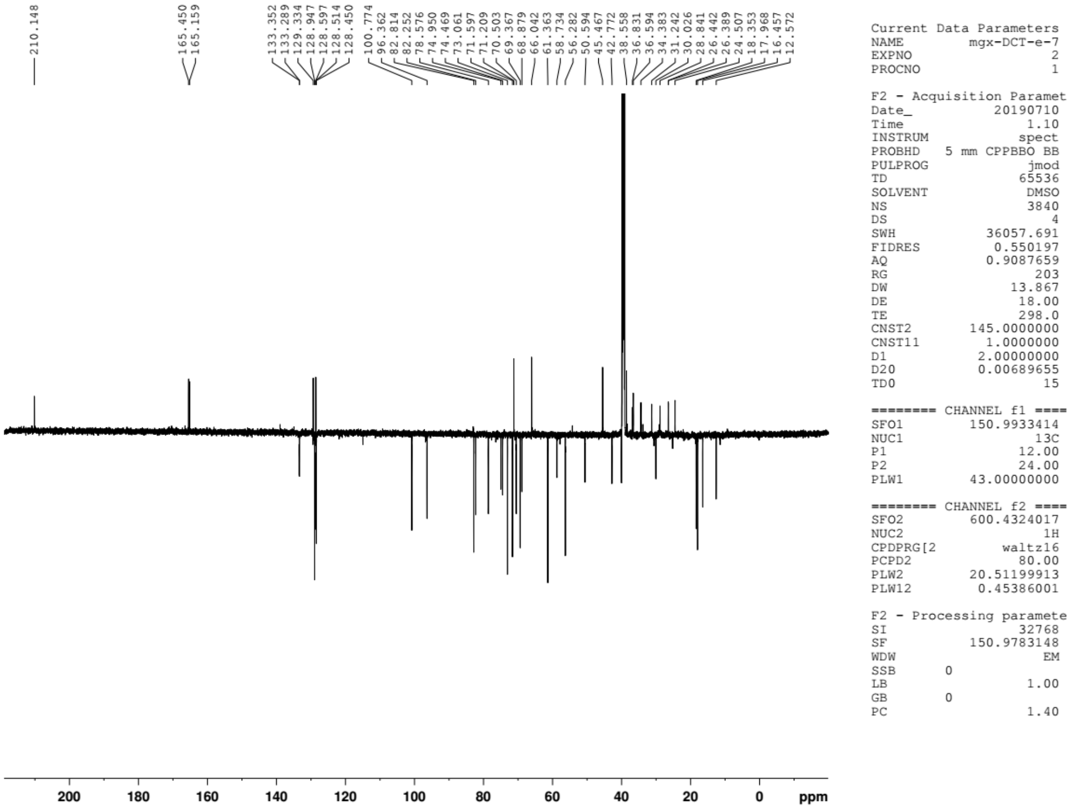

Figure S65. HSQC spectrum of the new compound **8**

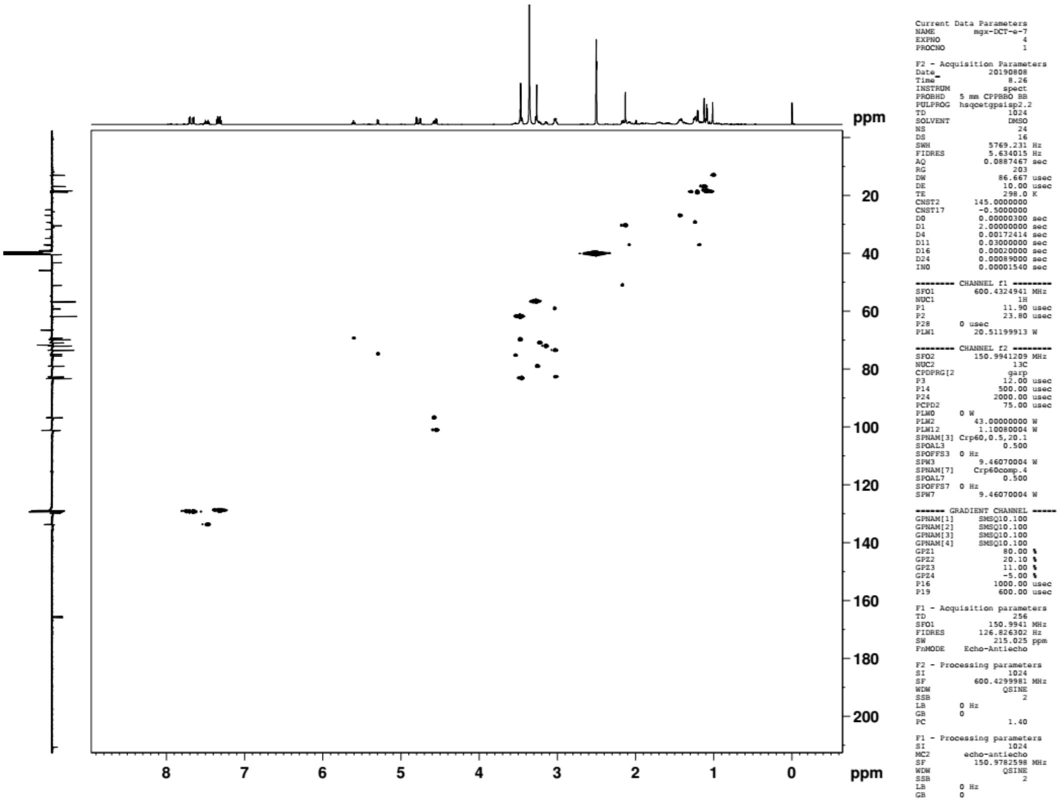

Figure S66. HMBC spectrum of the new compound **8**

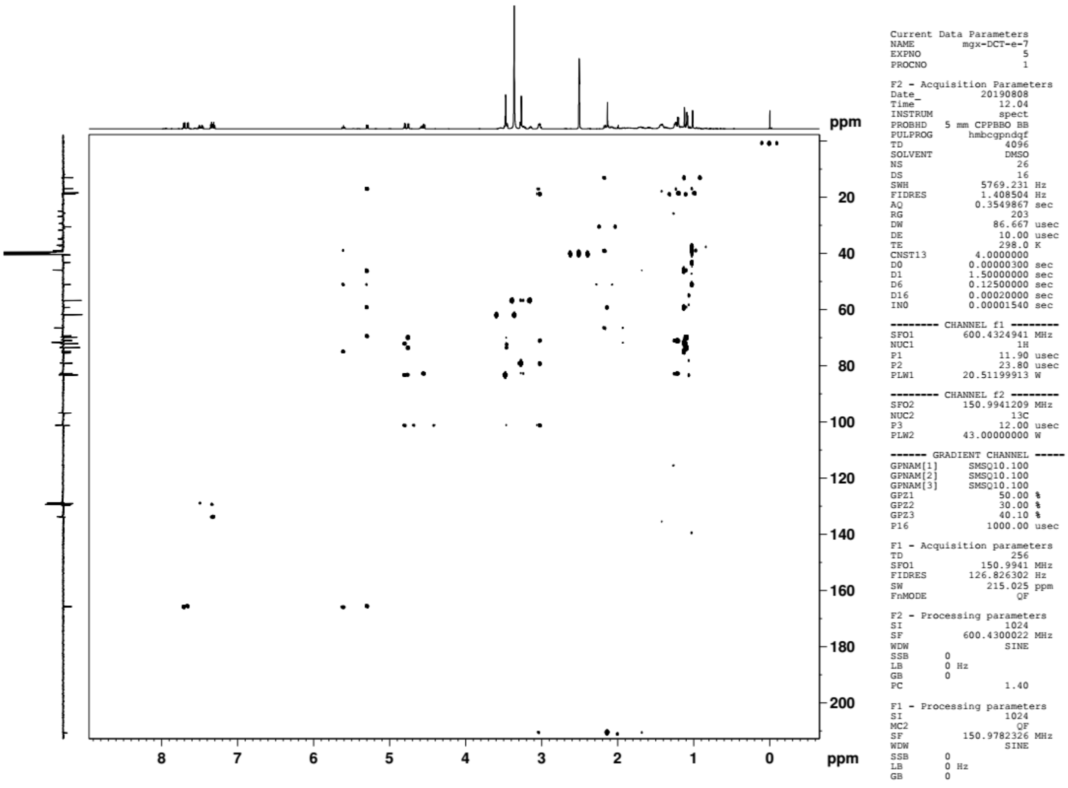

Figure S67. ^{1}H-^{1}H COSY spectrum of the new compound **8**

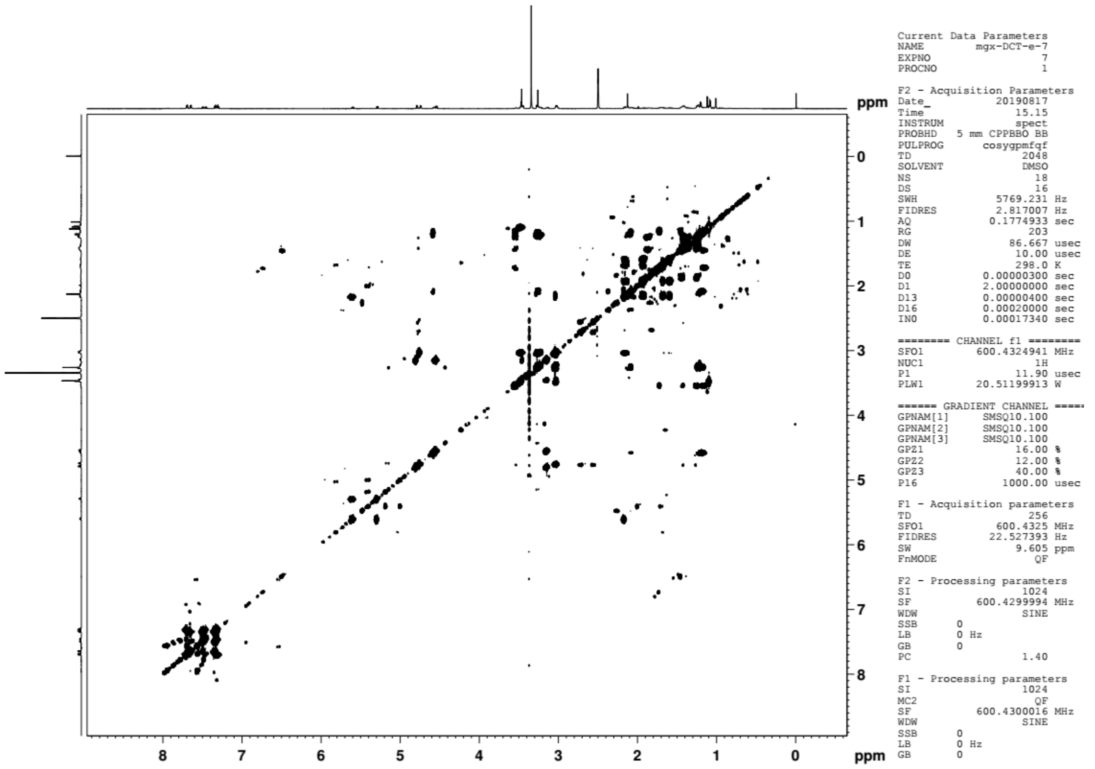

Figure S68. NOESY spectrum of the new compound **8**

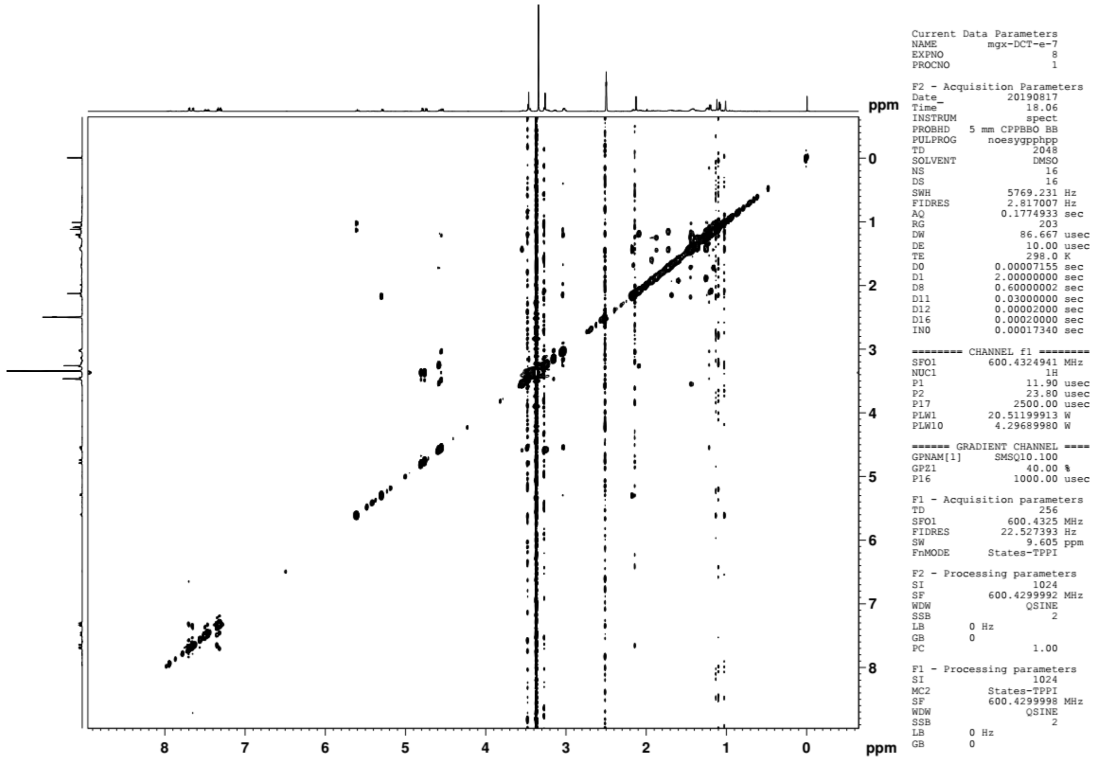

Figure S69. IR spectrum of the new compound **8**

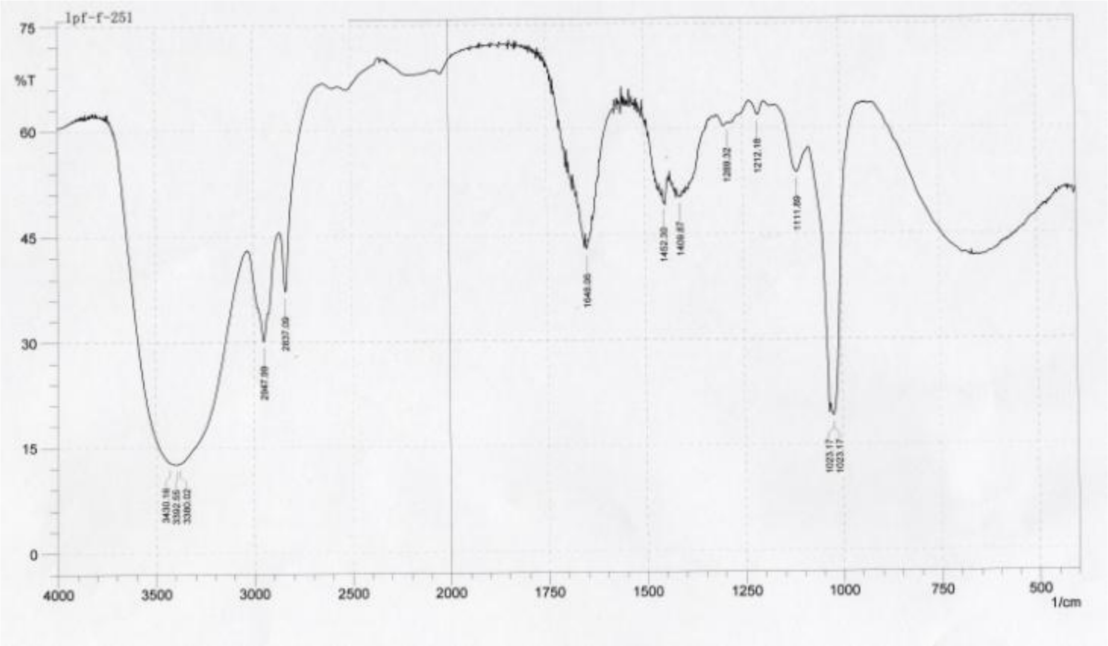

Figure S70. HRESIMS spectrum of the new compound **8**

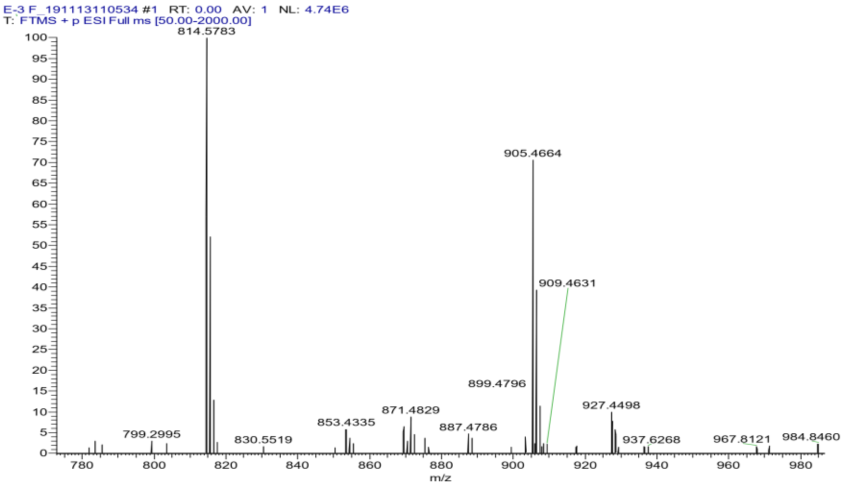

Figure S71. Key HMBC (Arrows) and ^1H-^1H COSY (Bonds) correlations of the new compound **8**

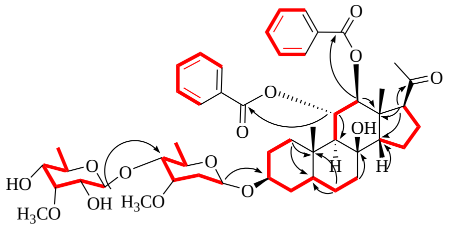

Figure S72. Calculated and experimental ECD spectrum of **1**

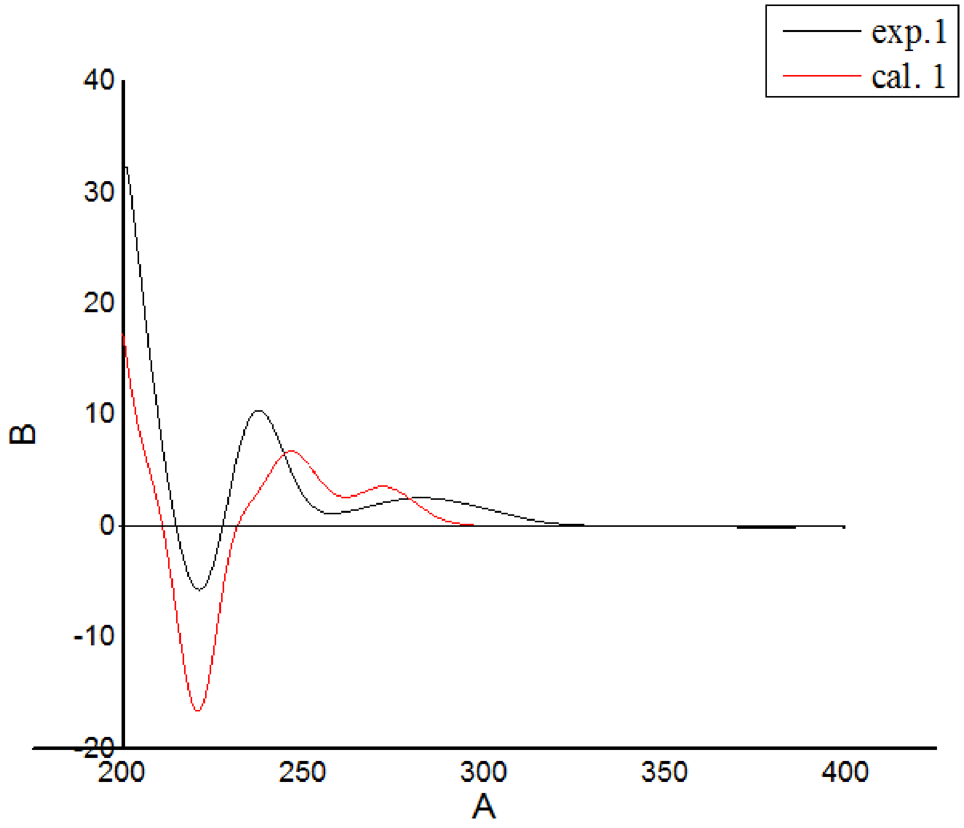

Figure S73. Calculated and experimental ECD spectrum of **2**

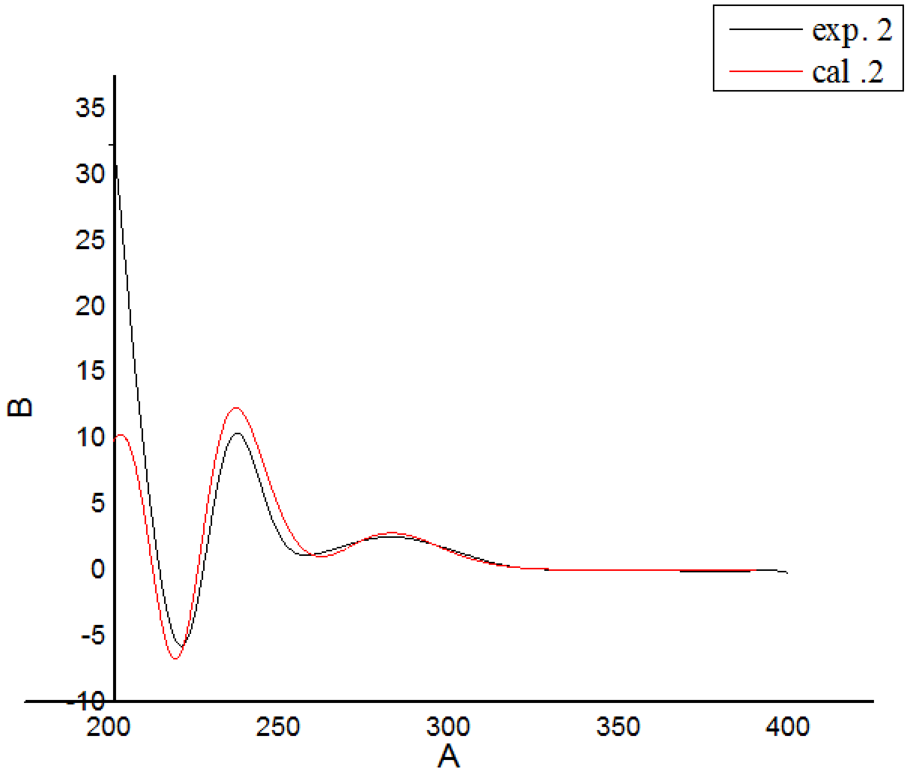

Figure S74. Calculated and experimental ECD spectrum of **3**

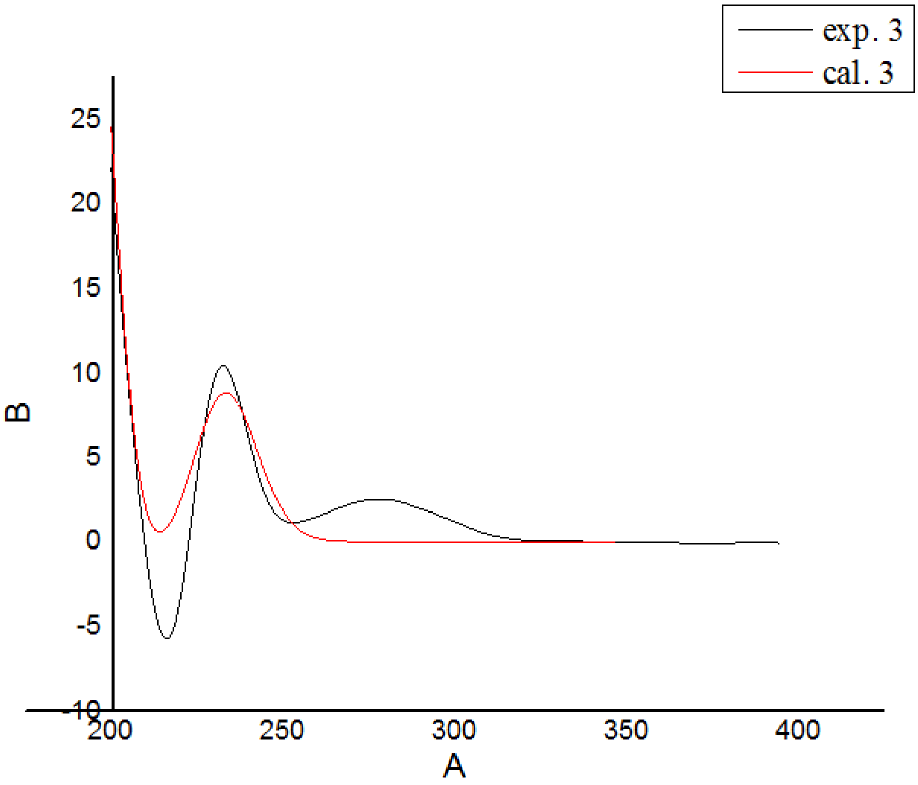

Figure S75. Calculated and experimental ECD spectrum of **4**

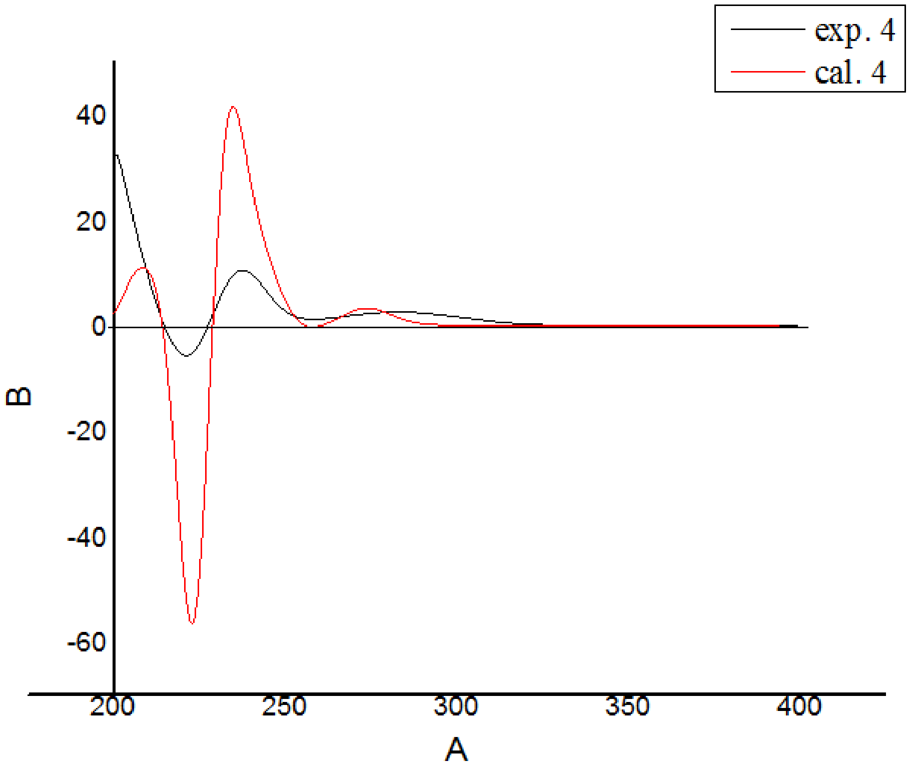

Figure S76. Calculated and experimental ECD spectrum of **5**

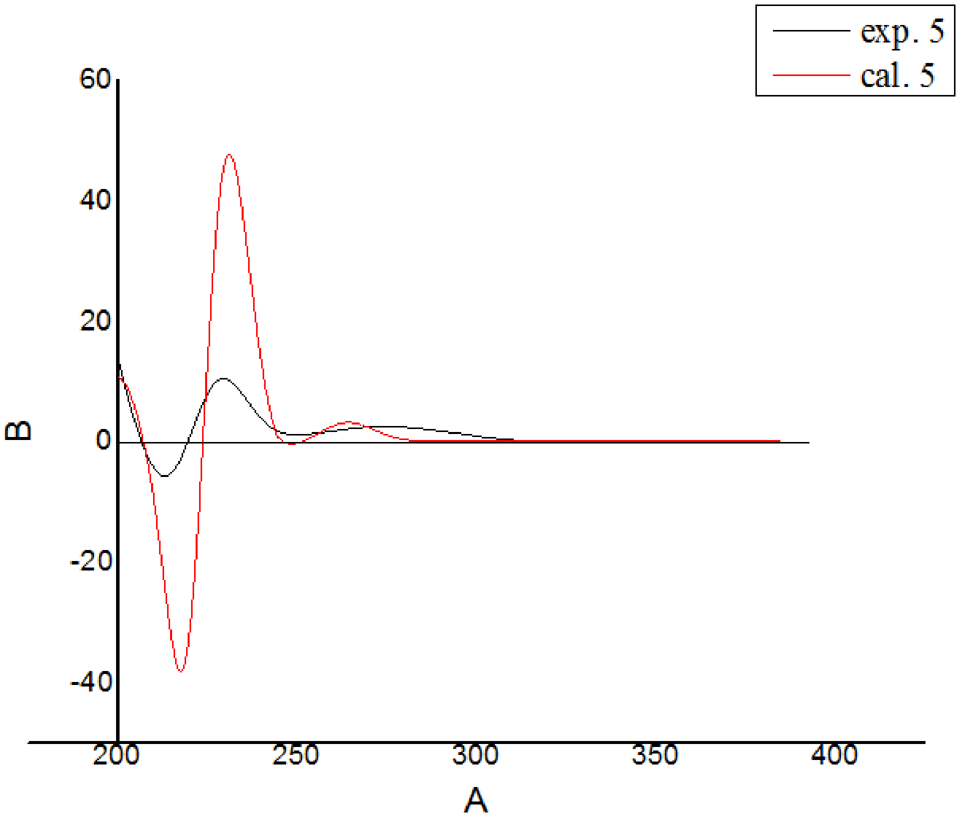

Figure S77. Calculated and experimental ECD spectrum of **6**

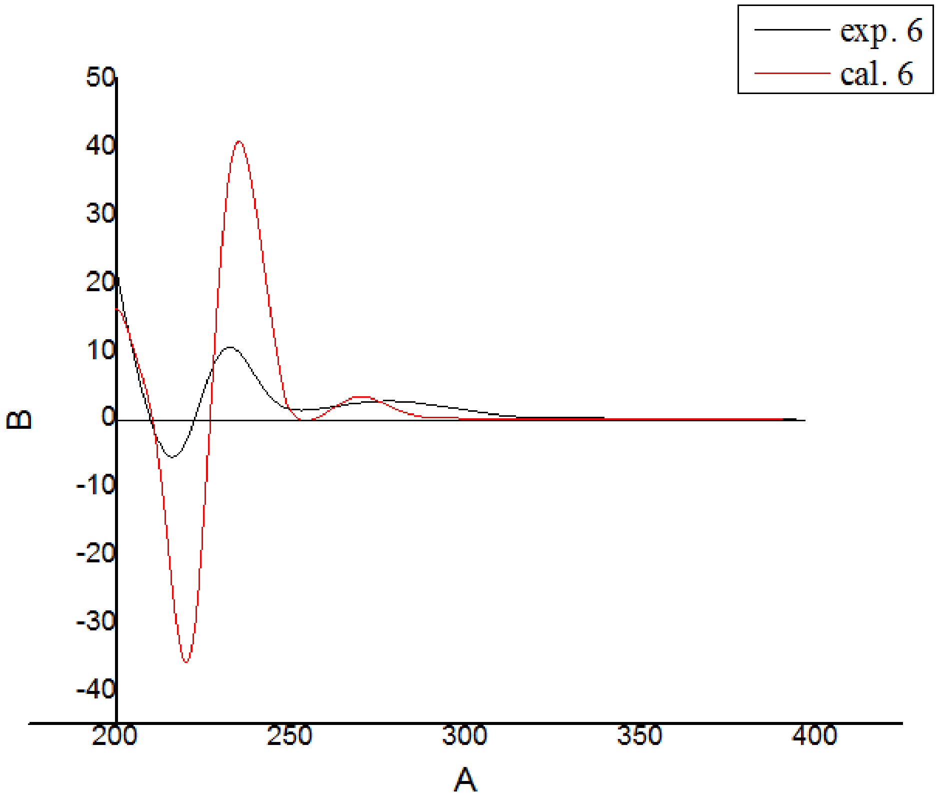

Figure S78. Calculated and experimental ECD spectrum of **7**

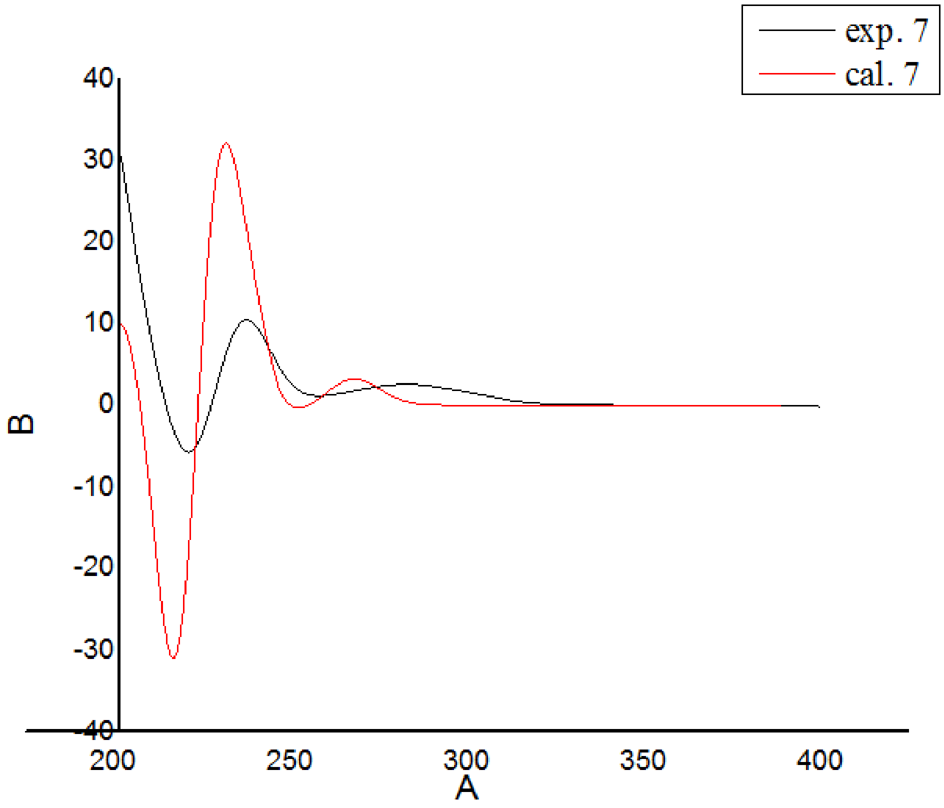

Figure S79. Calculated and experimental ECD spectrum of **8**

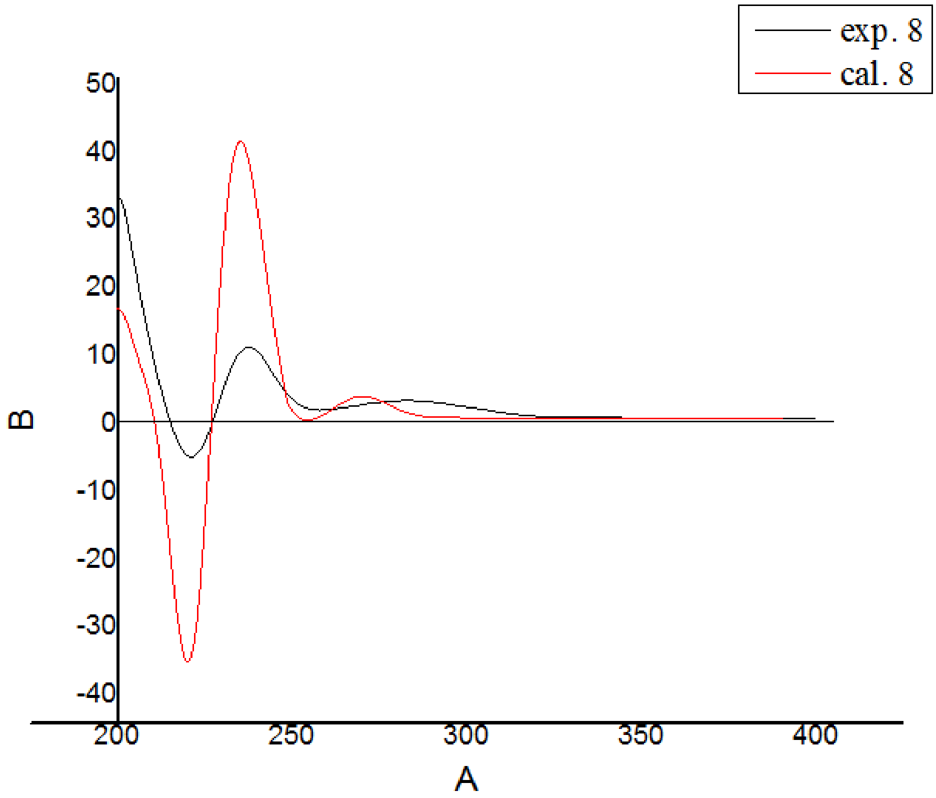

Supplement: Supplementary file 2 [file DataSheet1.pdf]
