# Supplementary material for: Discovery of Polyoxypregnane Derivatives From Aspidopterys obcordata With Their Potential Antitumor Activity
Source: Front Chem. 2022 Jan 5;9:799911. doi: 10.3389/fchem.2021.799911 (PMC8766633; doi:10.3389/fchem.2021.799911)
Supplement: Supplementary file 3 [file DataSheet2.ZIP › spectra/b-9/COSY.pdf]

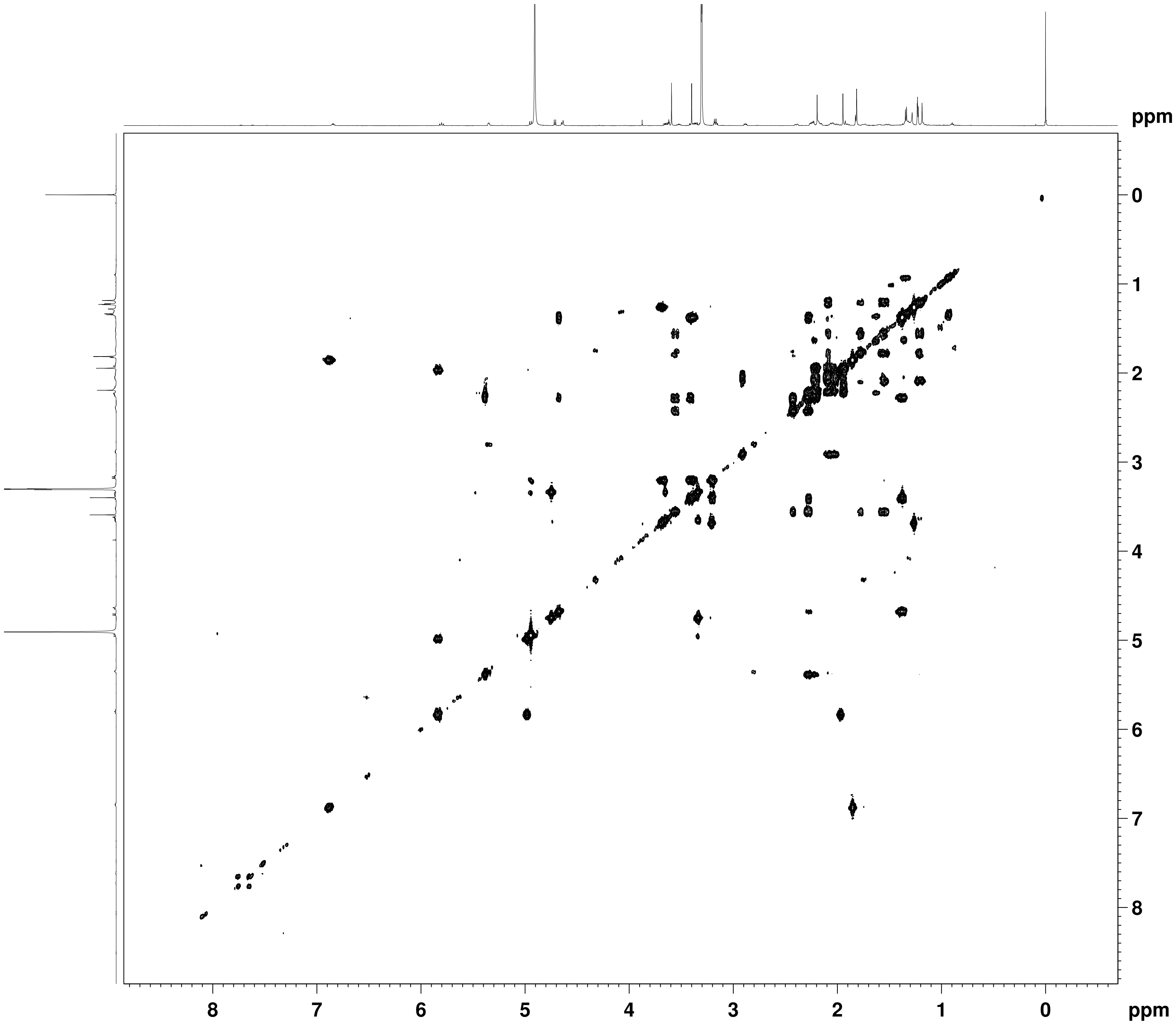


Current Data Parameters
NAME mgx-DCT-b-9
EXPNO 8
PROCNO 1

F2 - Acquisition Parameters
Date_ 20190816
Time 8.12
INSTRUM spect
PROBHD 5 mm CPPBBO BB
PULPROG cosygpmfqf
TD 2048
SOLVENT MeOD
NS 18
DS 16
SWH 5733.945 Hz
FIDRES 2.799778 Hz
AQ 0.1785856 sec
RG 203
DW 87.200 usec
DE 10.00 usec
TE 298.0 K
D0 0.00000300 sec
D1 2.00000000 sec
D13 0.00000400 sec
D16 0.00020000 sec
IN0 0.00017440 sec

======== CHANNEL f1 ========
SFO1 600.4324466 MHz
NUC1 1H
P1 11.90 usec
PLW1 20.51199913 W

====== GRADIENT CHANNEL =====
GPNAM[1] SMSQ10.100
GPNAM[2] SMSQ10.100
GPNAM[3] SMSQ10.100
GPZ1 16.00 %
GPZ2 12.00 %
GPZ3 40.00 %
P16 1000.00 usec

F1 - Acquisition parameters
TD 256
SFO1 600.4324 MHz
FIDRES 22.398222 Hz
SW 9.550 ppm
FnMODE QF

F2 - Processing parameters
SI 1024
SF 600.4299968 MHz
WDW SINE
SSB 0
LB 0 Hz
GB 0
PC 1.40

F1 - Processing parameters
SI 1024
MC2 QF
SF 600.4299953 MHz
WDW SINE
SSB 0
LB 0 Hz
GB 0
