# Supplementary material for: Discovery of Polyoxypregnane Derivatives From Aspidopterys obcordata With Their Potential Antitumor Activity
Source: Front Chem. 2022 Jan 5;9:799911. doi: 10.3389/fchem.2021.799911 (PMC8766633; doi:10.3389/fchem.2021.799911)
Supplement: Supplementary file 3 [file DataSheet2.ZIP › spectra/e-2-1/BC.pdf]

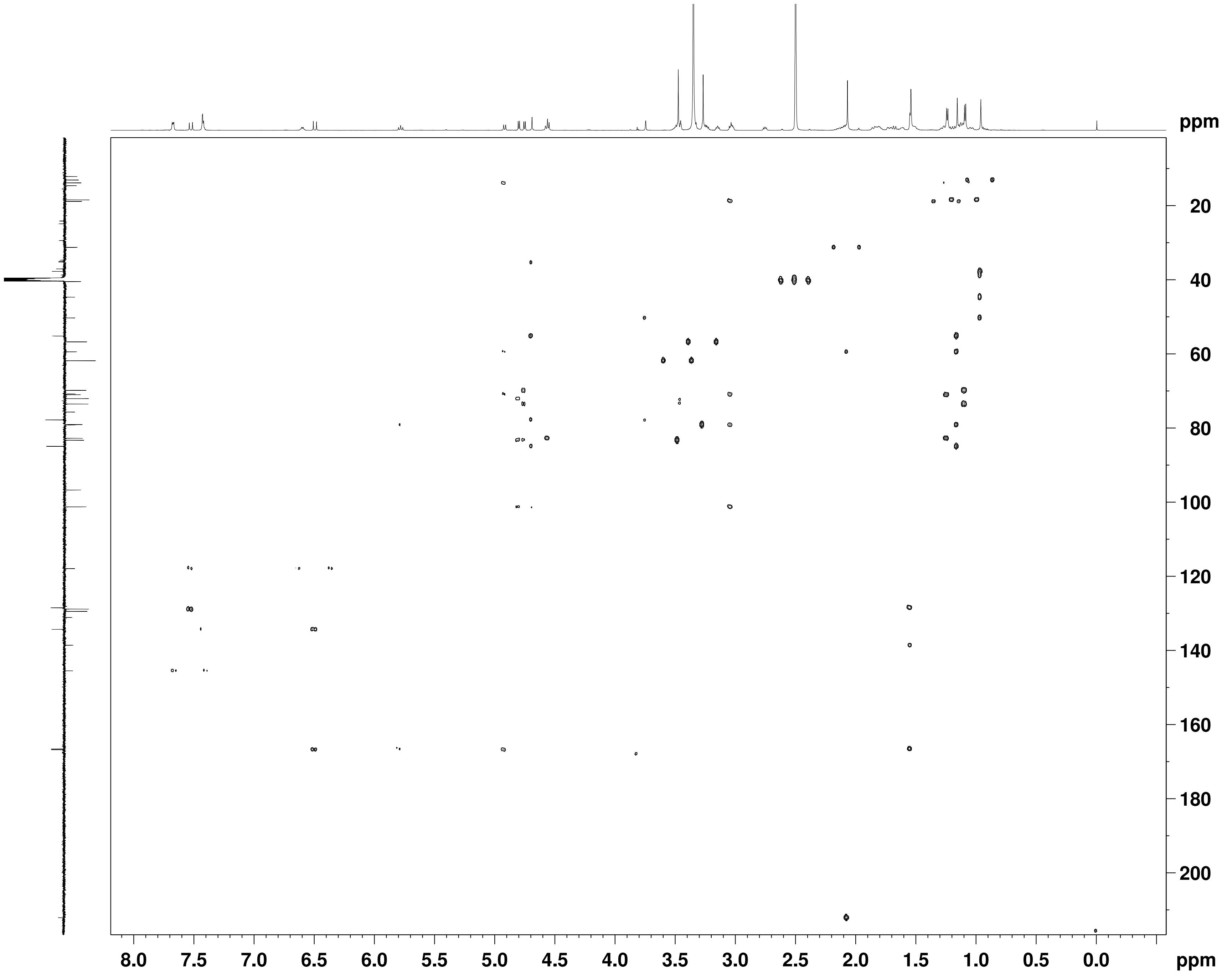

Current Data Parameters

NAME	mgx-DCT-e-2-1
EXPNO	5
PROCNO	1

F2 - Acquisition Parameter

Date_	20190823
Time	17.04
INSTRUM	spect
PROBHD	5 mm CPPBBO BB
PULPROG	hmbcgpndqf
TD	4096
SOLVENT	DMSO
NS	26
DS	16
SWH	5266.854 Hz
FIDRES	1.285853 Hz
AQ	0.3888469 se
RG	203
DW	94.933 us
DE	10.00 us
TE	298.0 K
CNST13	4.0000000
D0	0.00000300 se
D1	1.50000000 se
D6	0.12500000 se
D16	0.00020000 se
IN0	0.00001540 se

======== CHANNEL f1 ======

SFO1	600.4322848 MH
NUC1	1H
P1	11.90 us
P2	23.80 us
PLW1	20.51199913 W

======== CHANNEL f2 ======

SFO2	150.9947279 MH
NUC2	13C
P3	12.00 us
PLW2	43.00000000 W

====== GRADIENT CHANNEL ==

GPNAM[1]	SMSQ10.100
GPNAM[2]	SMSQ10.100
GPNAM[3]	SMSQ10.100
GPZ1	50.00 %
GPZ2	30.00 %
GPZ3	40.10 %
P16	1000.00 us

F1 - Acquisition parameter

TD	256
SFO1	150.9947 MH
FIDRES	126.826302 Hz
SW	215.024 pp
FnMODE	QF

F2 - Processing parameters

SI	1024
SF	600.4300000 MH
WDW	SINE
SSB	0
LB	0 Hz
GB	0
PC	1.40

F1 - Processing parameters

SI	1024
MC2	QF
SF	150.9782440 MH
WDW	SINE
SSB	0
LB	0 Hz
GB	0
