# Supplementary material for: Discovery of Polyoxypregnane Derivatives From Aspidopterys obcordata With Their Potential Antitumor Activity
Source: Front Chem. 2022 Jan 5;9:799911. doi: 10.3389/fchem.2021.799911 (PMC8766633; doi:10.3389/fchem.2021.799911)
Supplement: Supplementary file 3 [file DataSheet2.ZIP › spectra/e-2-1/C1.pdf]

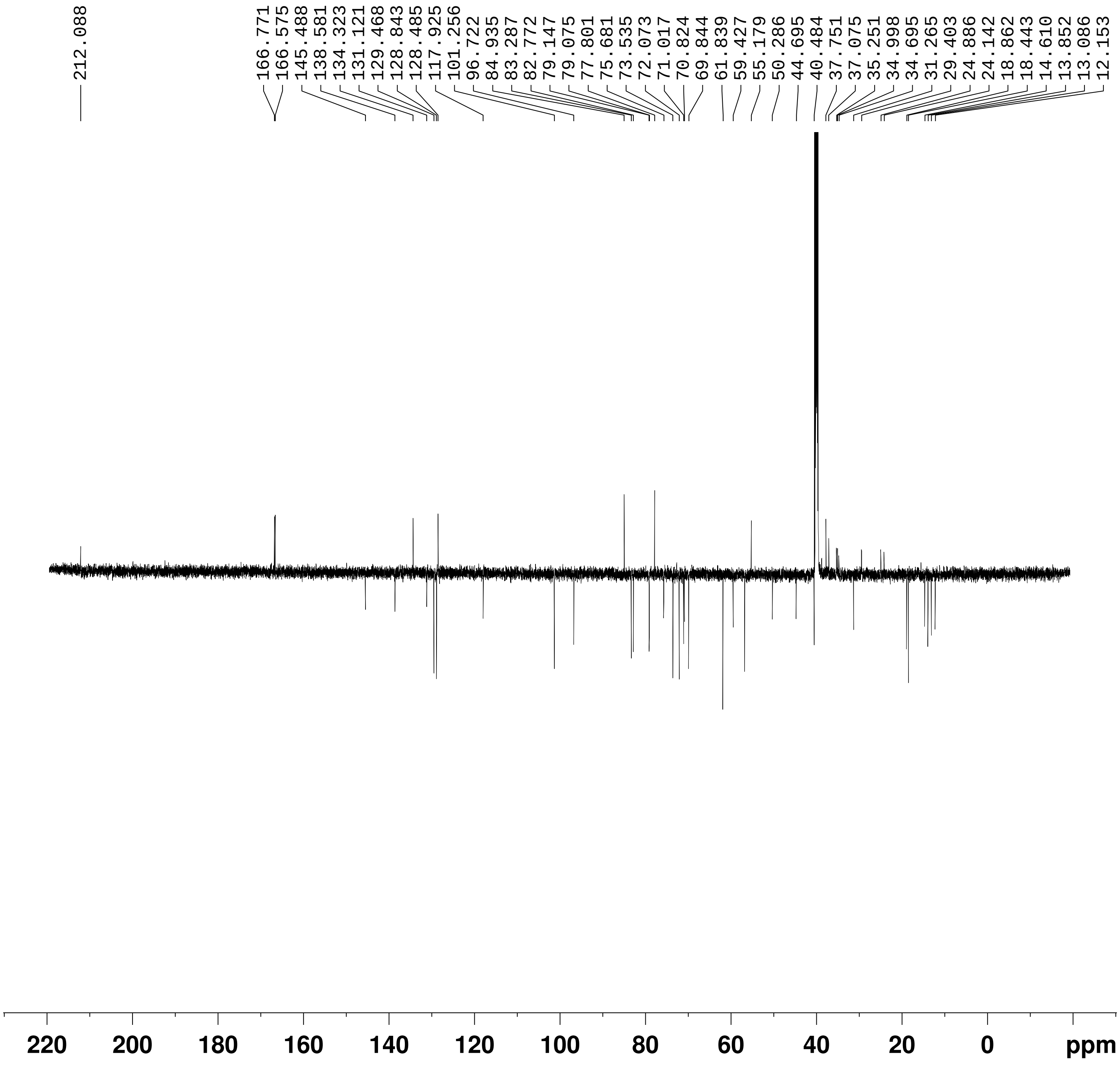


Current Data Parameters
NAME mgx-DCT-e-2-1
EXPNO 2
PROCNO 1

F2 - Acquisition Parameter
Date_ 20190822
Time 13.11
INSTRUM spect
PROBHD 5 mm CPPBBO BB
PULPROG jmod
TD 65536
SOLVENT DMSO
NS 1536
DS 4
SWH 36057.691 Hz
FIDRES 0.550197 Hz
AQ 0.9087659 se
RG 203
DW 13.867 us
DE 18.00 us
TE 298.0 K
CNST2 145.0000000
CNST11 1.0000000
D1 2.00000000 se
D20 0.00689655 se
TD0 6

======== CHANNEL f1 ======
SFO1 150.9933414 MH
NUC1 13C
P1 12.00 us
P2 24.00 us
PLW1 43.00000000 W

======== CHANNEL f2 ======
SFO2 600.4324017 MH
NUC2 1H
CPDPRG[2 waltz16
PCPD2 80.00 us
PLW2 20.51199913 W
PLW12 0.45386001 W

F2 - Processing parameters
SI 32768
SF 150.9782440 MH
WDW EM
SSB 0
LB 1.00 Hz
GB 0
PC 1.40
