# Supplementary material for: Discovery of Polyoxypregnane Derivatives From Aspidopterys obcordata With Their Potential Antitumor Activity
Source: Front Chem. 2022 Jan 5;9:799911. doi: 10.3389/fchem.2021.799911 (PMC8766633; doi:10.3389/fchem.2021.799911)
Supplement: Supplementary file 3 [file DataSheet2.ZIP › spectra/e-2-1/COSY.pdf]

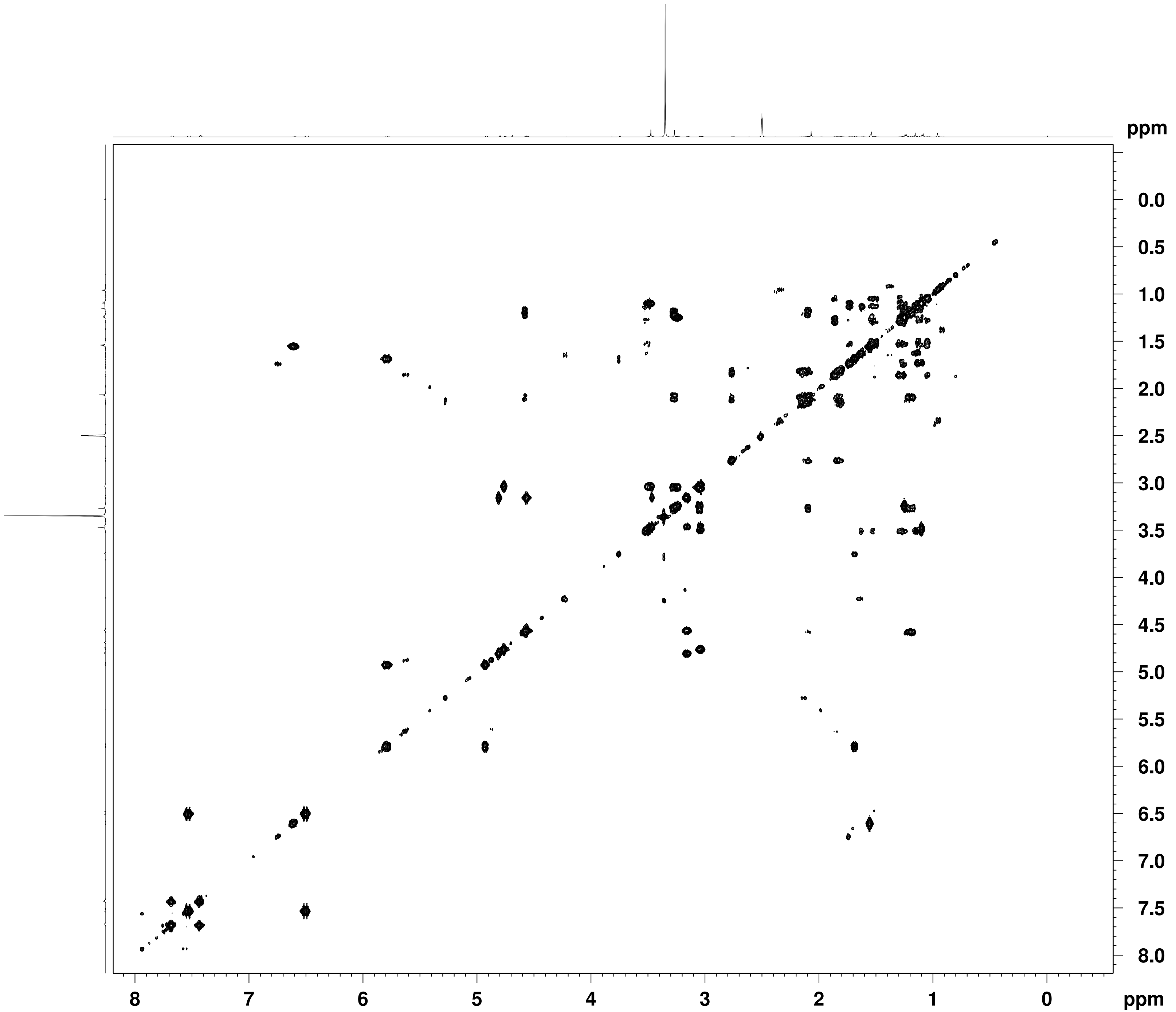


```
Current Data Parameters
NAME       mgx-DCT-e-2-1
EXPNO                  3
PROCNO                 1

F2 - Acquisition Parameters
Date_           20190823
Time               10.51
INSTRUM            spect
PROBHD   5 mm CPPBBO BB
PULPROG      cosygpmfqf
TD                  2048
SOLVENT             DMSO
NS                    16
DS                    16
SWH            5266.854 Hz
FIDRES         2.571706 Hz
AQ            0.1944235 sec
RG                   203
DW                94.933 usec
DE                 10.00 usec
TE                 298.0 K
D0            0.00000300 sec
D1            2.00000000 sec
D13           0.00000400 sec
D16           0.00020000 sec
IN0           0.00018980 sec

======== CHANNEL f1 ========
SFO1         600.4322848 MHz
NUC1                  1H
P1                 11.90 usec
PLW1         20.51199913 W

====== GRADIENT CHANNEL =====
GPNAM[1]      SMSQ10.100
GPNAM[2]      SMSQ10.100
GPNAM[3]      SMSQ10.100
GPZ1               16.00 %
GPZ2               12.00 %
GPZ3               40.00 %
P16              1000.00 usec

F1 - Acquisition parameters
TD                   256
SFO1            600.4323 MHz
FIDRES         20.580875 Hz
SW                 8.775 ppm
FnMODE                QF

F2 - Processing parameters
SI                  1024
SF           600.4300001 MHz
WDW                 SINE
SSB      0
LB       0 Hz
GB       0
PC                  1.40

F1 - Processing parameters
SI                  1024
MC2                   QF
SF           600.4300003 MHz
WDW                 SINE
SSB      0
LB       0 Hz
GB       0
```
