# Supplementary material for: Discovery of Polyoxypregnane Derivatives From Aspidopterys obcordata With Their Potential Antitumor Activity
Source: Front Chem. 2022 Jan 5;9:799911. doi: 10.3389/fchem.2021.799911 (PMC8766633; doi:10.3389/fchem.2021.799911)
Supplement: Supplementary file 3 [file DataSheet2.ZIP › spectra/e-2-1/H1.pdf]

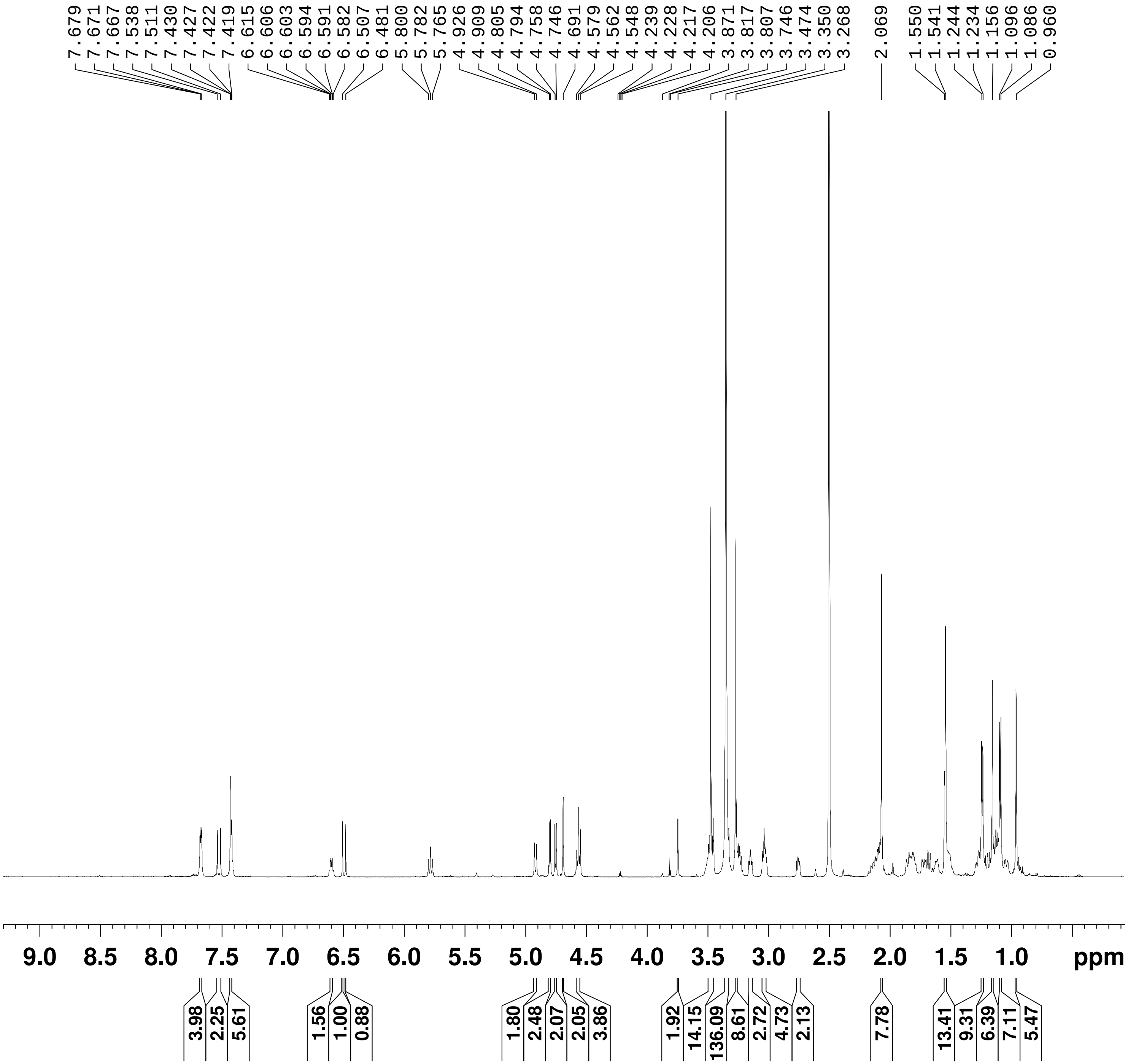


```
Current Data Parameters
NAME       mgx-DCT-e-2-1
EXPNO                  1
PROCNO                 1

F2 - Acquisition Parameter
Date_           20190820
Time               15.28
INSTRUM            spect
PROBHD    5 mm CPPBBO BB
PULPROG             zg30
TD                 65536
SOLVENT             DMSO
NS                    16
DS                     2
SWH            12019.230 Hz
FIDRES          0.183399 Hz
AQ             2.7262976 se
RG                  90.5
DW                41.600 us
DE                 10.00 us
TE                 298.0 K
D1            1.00000000 se
TD0                    1

======== CHANNEL f1 ======
SFO1         600.4337079 MH
NUC1                  1H
P1                 11.90 us
PLW1         20.51199913 W

F2 - Processing parameters
SI                 65536
SF           600.4300085 MH
WDW                   EM
SSB      0
LB                  0.30 Hz
GB       0
PC                  1.00
```
