# Supplementary material for: Discovery of Polyoxypregnane Derivatives From Aspidopterys obcordata With Their Potential Antitumor Activity
Source: Front Chem. 2022 Jan 5;9:799911. doi: 10.3389/fchem.2021.799911 (PMC8766633; doi:10.3389/fchem.2021.799911)
Supplement: Supplementary file 3 [file DataSheet2.ZIP › spectra/e-2-2/BC.pdf]

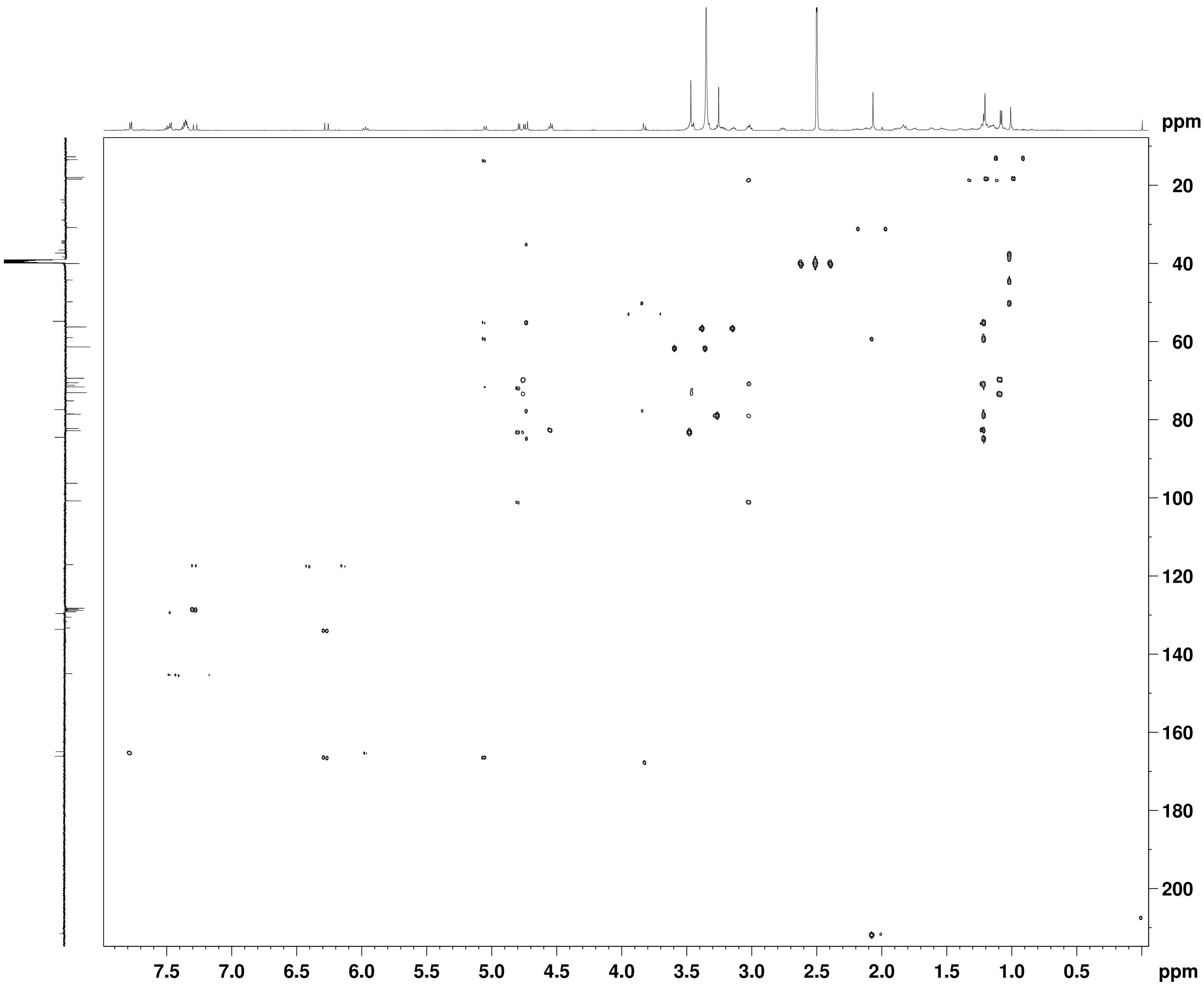

Current Data Parameters
NAME mgx-DCT-e-2-2
EXPNO 5
PROCNO 1

F2 - Acquisition Parameters
Date_ 20190824
Time 6.08
INSTRUM spect
PROBHD 5 mm CPPBBO BB
PULPROG hmbcgpndqf
TD 4096
SOLVENT DMSO
NS 26
DS 16
SWH 4826.255 Hz
FIDRES 1.178285 Hz
AQ 0.4243456 sec
RG 203
DW 103.600 usec
DE 10.00 usec
TE 298.0 K
CNST13 4.0000000
D0 0.00000300 sec
D1 1.50000000 sec
D6 0.12500000 sec
D16 0.00020000 sec
IN0 0.00001600 sec

======== CHANNEL f1 ========
SFO1 600.4323815 MHz
NUC1 1H
P1 11.90 usec
P2 23.80 usec
PLW1 20.51199913 W

======== CHANNEL f2 ========
SFO2 150.9950477 MHz
NUC2 13C
P3 12.00 usec
PLW2 43.00000000 W

====== GRADIENT CHANNEL =====
GPNAM[1] SMSQ10.100
GPNAM[2] SMSQ10.100
GPNAM[3] SMSQ10.100
GPZ1 50.00 %
GPZ2 30.00 %
GPZ3 40.10 %
P16 1000.00 usec

F1 - Acquisition parameters
TD 256
SFO1 150.995 MHz
FIDRES 122.070313 Hz
SW 206.960 ppm
FnMODE QF

F2 - Processing parameters
SI 1024
SF 600.4300000 MHz
WDW SINE
SSB 0
LB 0 Hz
GB 0
PC 1.40

F1 - Processing parameters
SI 1024
MC2 QF
SF 150.9782440 MHz
WDW SINE
SSB 0
LB 0 Hz
GB 0
