# Supplementary material for: Discovery of Polyoxypregnane Derivatives From Aspidopterys obcordata With Their Potential Antitumor Activity
Source: Front Chem. 2022 Jan 5;9:799911. doi: 10.3389/fchem.2021.799911 (PMC8766633; doi:10.3389/fchem.2021.799911)
Supplement: Supplementary file 3 [file DataSheet2.ZIP › spectra/e-2-2/COSY.pdf]

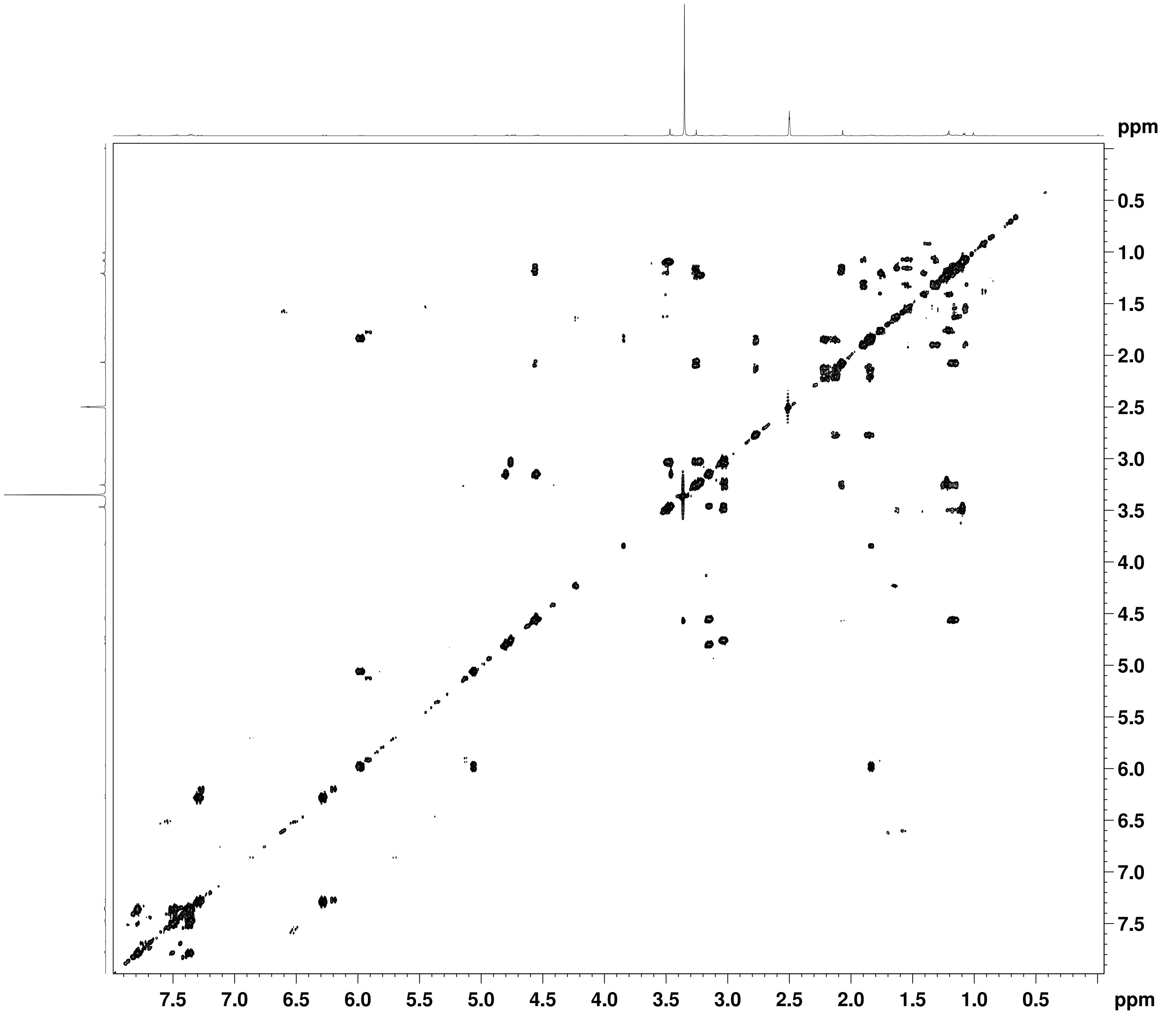


```
Current Data Parameters
NAME      mgx-DCT-e-2-2
EXPNO                 3
PROCNO                1

F2 - Acquisition Parameters
Date_          20190823
Time              23.53
INSTRUM           spect
PROBHD   5 mm CPPBBO BB
PULPROG       cosygpmfqf
TD                 2048
SOLVENT            DMSO
NS                   16
DS                   16
SWH            4826.255 Hz
FIDRES         2.356570 Hz
AQ            0.2121728 sec
RG                  203
DW              103.600 usec
DE                10.00 usec
TE                298.0 K
D0           0.00000300 sec
D1           2.00000000 sec
D13          0.00000400 sec
D16          0.00020000 sec
IN0          0.00020720 sec

======== CHANNEL f1 ========
SFO1        600.4323815 MHz
NUC1                 1H
P1                11.90 usec
PLW1        20.51199913 W

====== GRADIENT CHANNEL =====
GPNAM[1]     SMSQ10.100
GPNAM[2]     SMSQ10.100
GPNAM[3]     SMSQ10.100
GPZ1              16.00 %
GPZ2              12.00 %
GPZ3              40.00 %
P16             1000.00 usec

F1 - Acquisition parameters
TD                  256
SFO1           600.4324 MHz
FIDRES        18.852558 Hz
SW                8.038 ppm
FnMODE               QF

F2 - Processing parameters
SI                 1024
SF          600.4300002 MHz
WDW                SINE
SSB      0
LB       0 Hz
GB       0
PC                 1.40

F1 - Processing parameters
SI                 1024
MC2                  QF
SF          600.4299995 MHz
WDW                SINE
SSB      0
LB       0 Hz
GB       0
```
