# Supplementary material for: Discovery of Polyoxypregnane Derivatives From Aspidopterys obcordata With Their Potential Antitumor Activity
Source: Front Chem. 2022 Jan 5;9:799911. doi: 10.3389/fchem.2021.799911 (PMC8766633; doi:10.3389/fchem.2021.799911)
Supplement: Supplementary file 3 [file DataSheet2.ZIP › spectra/e-2-2/H.pdf]

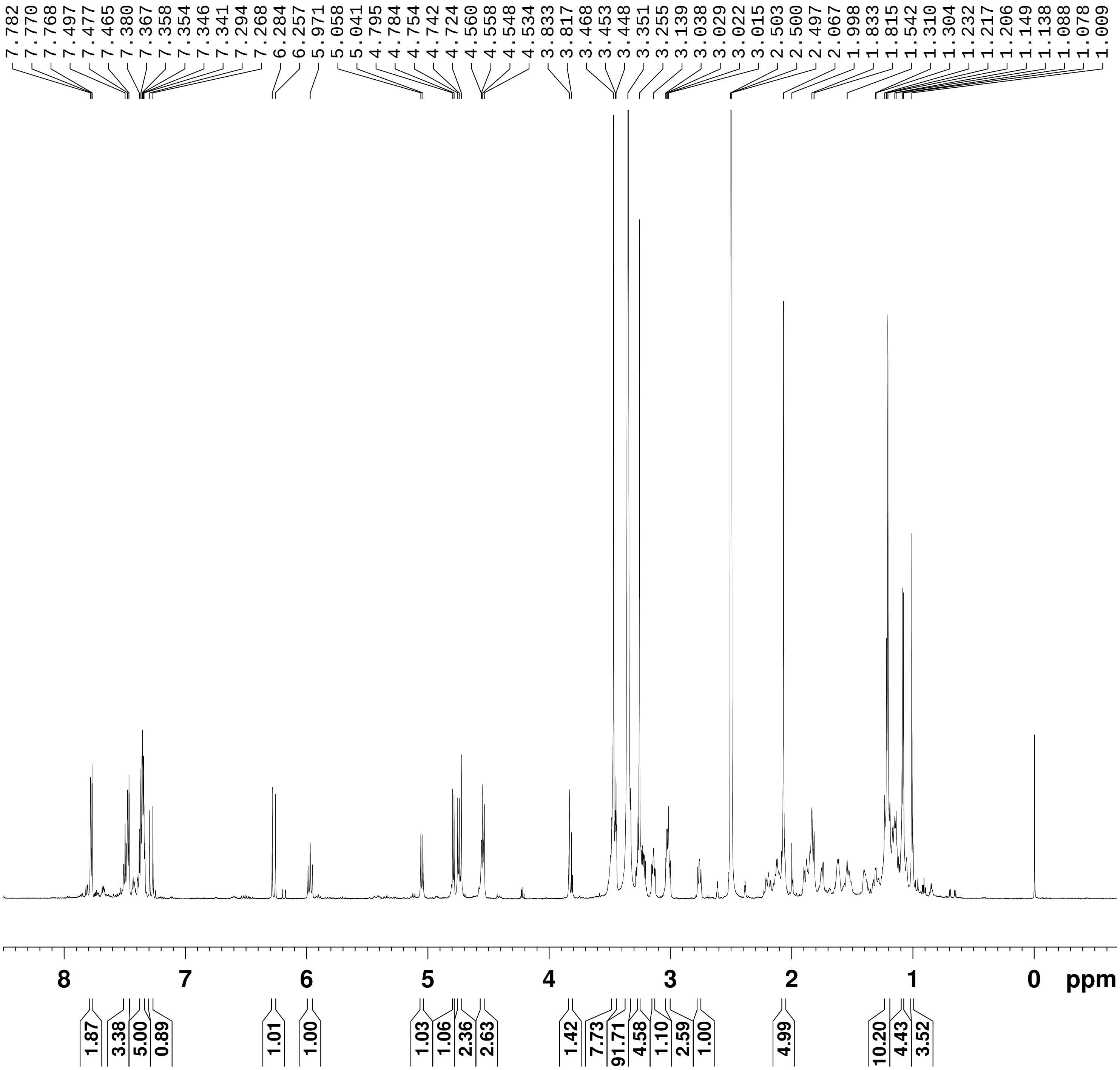


```
Current Data Parameters
NAME          mgx-DCT-e-2-2
EXPNO                     1
PROCNO                    1

F2 - Acquisition Parameter
Date_              20190820
Time                  15.33
INSTRUM               spect
PROBHD     5 mm CPPBBO BB
PULPROG                zg30
TD                    65536
SOLVENT                DMSO
NS                       16
DS                        2
SWH              12019.230 Hz
FIDRES            0.183399 Hz
AQ               2.7262976 se
RG                     90.5
DW                   41.600 us
DE                    10.00 us
TE                    298.0 K
D1               1.00000000 se
TD0                       1

======== CHANNEL f1 ======
SFO1           600.4337079 MH
NUC1                     1H
P1                    11.90 us
PLW1           20.51199913 W

F2 - Processing parameters
SI                    65536
SF             600.4300084 MH
WDW                      EM
SSB         0
LB                     0.30 Hz
GB          0
PC                     1.00
```
