# Supplementary material for: Discovery of Polyoxypregnane Derivatives From Aspidopterys obcordata With Their Potential Antitumor Activity
Source: Front Chem. 2022 Jan 5;9:799911. doi: 10.3389/fchem.2021.799911 (PMC8766633; doi:10.3389/fchem.2021.799911)
Supplement: Supplementary file 3 [file DataSheet2.ZIP › spectra/e-2-2/NOE.pdf]

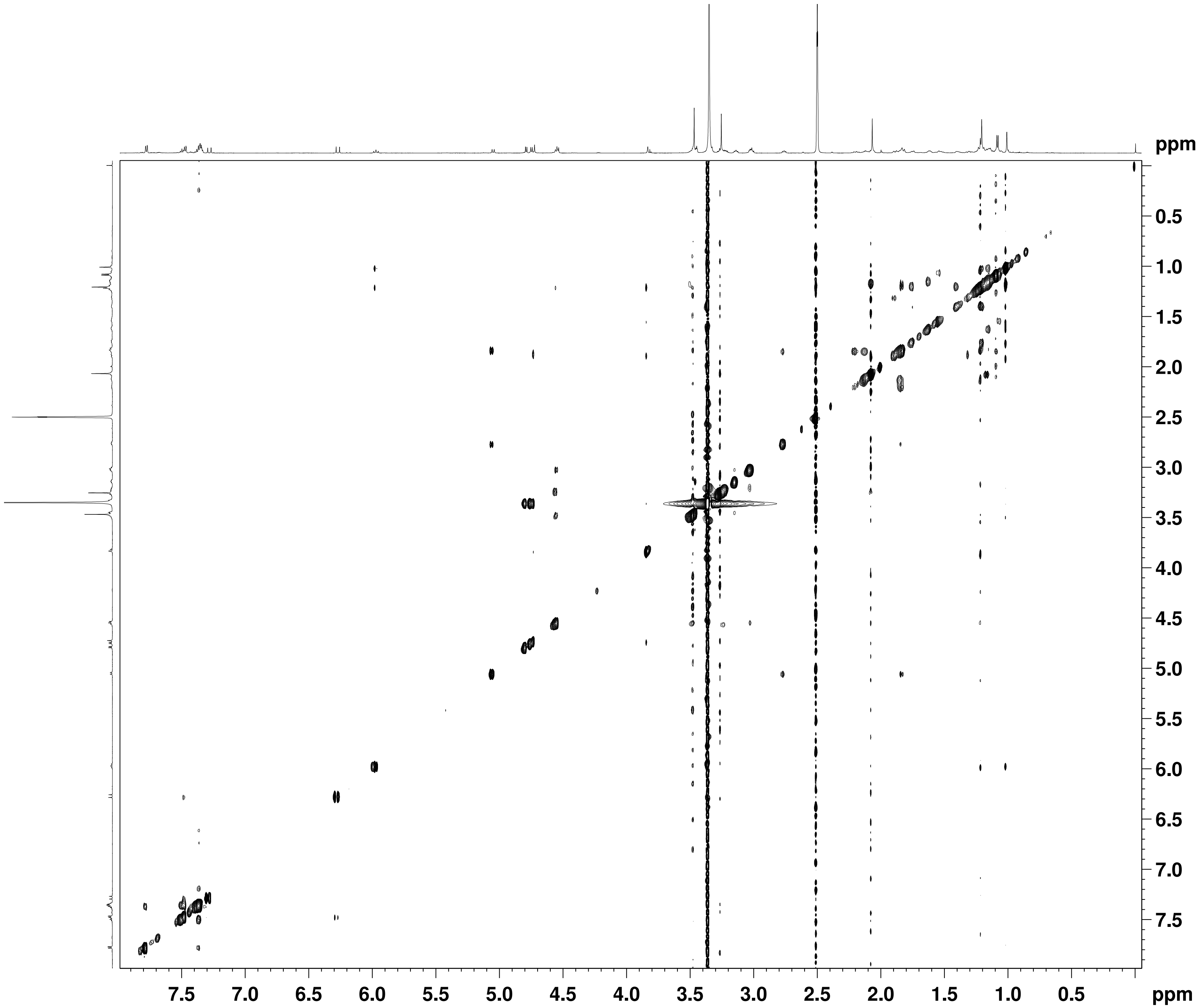

Current Data Parameters
NAME mgx-DCT-e-2-2
EXPNO 6
PROCNO 1

F2 - Acquisition Parameters
Date_ 20190824
Time 9.58
INSTRUM spect
PROBHD 5 mm CPPBBO BB
PULPROG noesygpphpp
TD 2048
SOLVENT DMSO
NS 16
DS 16
SWH 4826.255 Hz
FIDRES 2.356570 Hz
AQ 0.2121728 sec
RG 203
DW 103.600 usec
DE 10.00 usec
TE 298.0 K
D0 0.00008845 sec
D1 2.00000000 sec
D8 0.60000002 sec
D11 0.03000000 sec
D12 0.00002000 sec
D16 0.00020000 sec
IN0 0.00020720 sec

======== CHANNEL f1 ========
SFO1 600.4323815 MHz
NUC1 1H
P1 11.90 usec
P2 23.80 usec
P17 2500.00 usec
PLW1 20.51199913 W
PLW10 4.29689980 W

====== GRADIENT CHANNEL =====
GPNAM[1] SMSQ10.100
GPZ1 40.00 %
P16 1000.00 usec

F1 - Acquisition parameters
TD 256
SFO1 600.4324 MHz
FIDRES 18.852558 Hz
SW 8.038 ppm
FnMODE States-TPPI

F2 - Processing parameters
SI 1024
SF 600.4300000 MHz
WDW QSINE
SSB 2
LB 0 Hz
GB 0
PC 1.00

F1 - Processing parameters
SI 1024
MC2 States-TPPI
SF 600.4300000 MHz
WDW QSINE
SSB 2
LB 0 Hz
GB 0
