# Supplementary material for: Discovery of Polyoxypregnane Derivatives From Aspidopterys obcordata With Their Potential Antitumor Activity
Source: Front Chem. 2022 Jan 5;9:799911. doi: 10.3389/fchem.2021.799911 (PMC8766633; doi:10.3389/fchem.2021.799911)
Supplement: Supplementary file 3 [file DataSheet2.ZIP › spectra/e-2-2/QC.pdf]

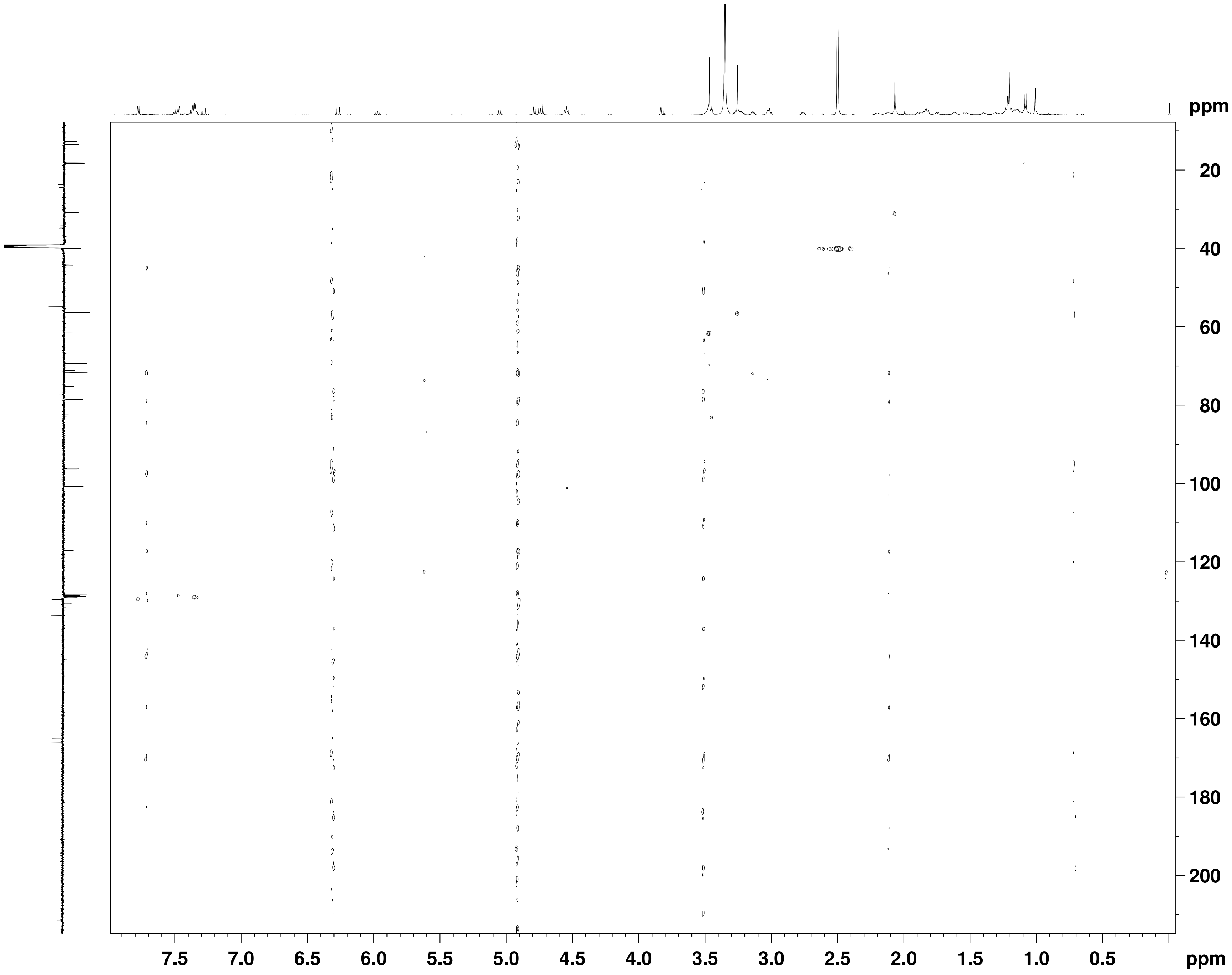


```
Current Data Parameters
NAME        mgx-DCT-e-2-2
EXPNO                   4
PROCNO                  1

F2 - Acquisition Parameters
Date_            20190824
Time                 2.28
INSTRUM             spect
PROBHD   5 mm CPPBBO BB
PULPROG  hsqcetgpsisp2.2
TD                   1024
SOLVENT              DMSO
NS                     24
DS                     16
SWH              4826.255 Hz
FIDRES           4.713140 Hz
AQ              0.1060864 sec
RG                    203
DW                103.600 usec
DE                  10.00 usec
TE                  298.0 K
CNST2         145.0000000
CNST17         -0.5000000
D0             0.00000300 sec
D1             2.00000000 sec
D4             0.00172414 sec
D11            0.03000000 sec
D16            0.00020000 sec
D24            0.00089000 sec
IN0            0.00001600 sec

======== CHANNEL f1 ========
SFO1        600.4323815 MHz
NUC1                   1H
P1                  11.90 usec
P2                  23.80 usec
P28      0 usec
PLW1          20.51199913 W

======== CHANNEL f2 ========
SFO2        150.9950477 MHz
NUC2                  13C
CPDPRG[2             garp
P3                  12.00 usec
P14                500.00 usec
P24               2000.00 usec
PCPD2               75.00 usec
PLW0     0 W
PLW2          43.00000000 W
PLW12          1.10080004 W
SPNAM[3] Crp60,0.5,20.1
SPOAL3              0.500
SPOFFS3  0 Hz
SPW3           9.46070004 W
SPNAM[7]    Crp60comp.4
SPOAL7              0.500
SPOFFS7  0 Hz
SPW7           9.46070004 W

====== GRADIENT CHANNEL =====
GPNAM[1]      SMSQ10.100
GPNAM[2]      SMSQ10.100
GPNAM[3]      SMSQ10.100
GPNAM[4]      SMSQ10.100
GPZ1                80.00 %
GPZ2                20.10 %
GPZ3                11.00 %
GPZ4                -5.00 %
P16               1000.00 usec
P19                600.00 usec

F1 - Acquisition parameters
TD                    256
SFO1              150.995 MHz
FIDRES         122.070313 Hz
SW                206.960 ppm
FnMODE     Echo-Antiecho

F2 - Processing parameters
SI                   1024
SF            600.4300000 MHz
WDW                 QSINE
SSB                     2
LB       0 Hz
GB       0
PC                   1.40

F1 - Processing parameters
SI                   1024
MC2          echo-antiecho
SF            150.9782440 MHz
WDW                 QSINE
SSB                     2
LB       0 Hz
GB       0
```
