# Supplementary material for: Discovery of Polyoxypregnane Derivatives From Aspidopterys obcordata With Their Potential Antitumor Activity
Source: Front Chem. 2022 Jan 5;9:799911. doi: 10.3389/fchem.2021.799911 (PMC8766633; doi:10.3389/fchem.2021.799911)
Supplement: Supplementary file 3 [file DataSheet2.ZIP › spectra/e-3/BC.pdf]

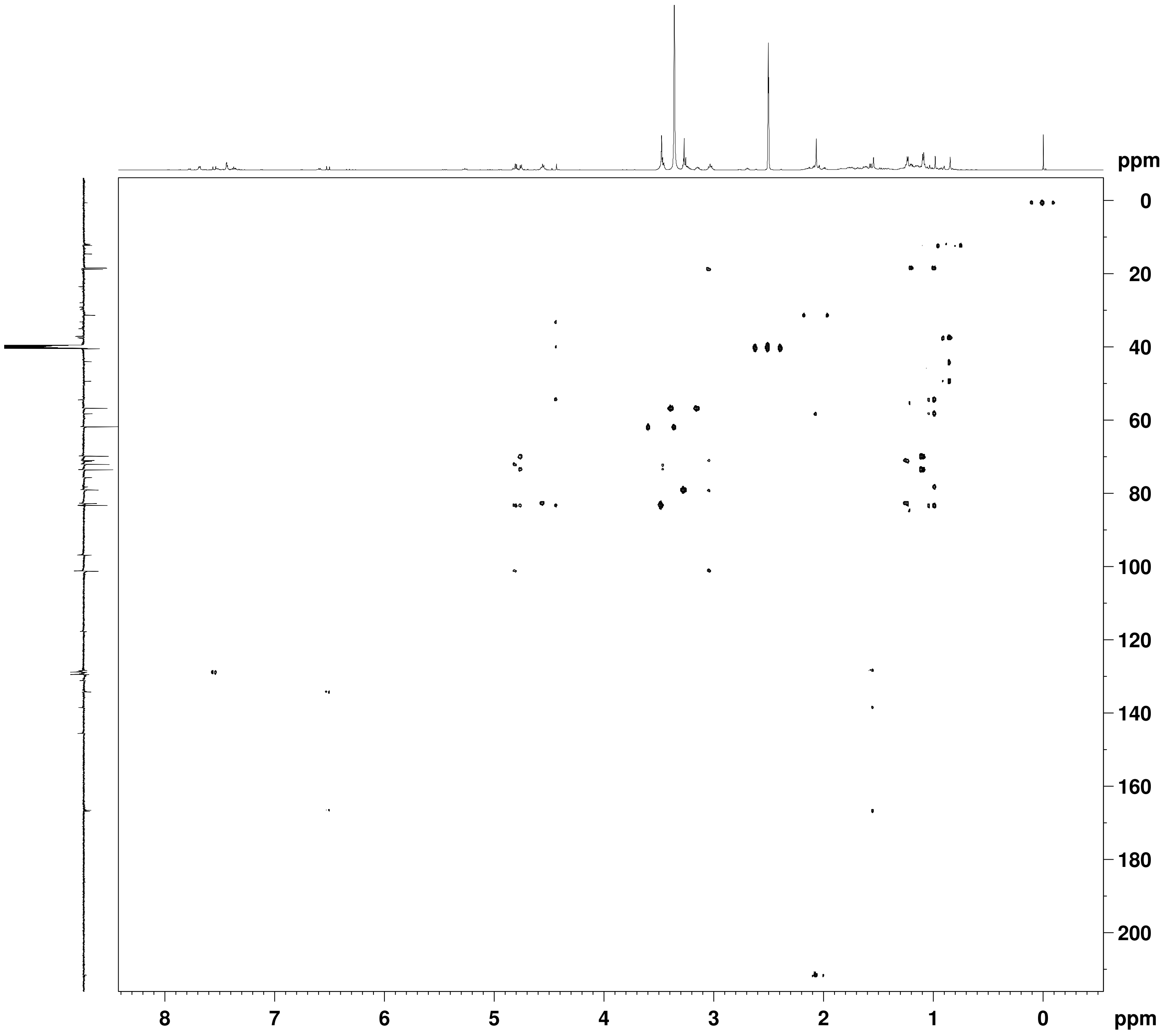


```
Current Data Parameters
NAME        mgx-DCT-e-3
EXPNO                 5
PROCNO                1

F2 - Acquisition Parameters
Date_          20190807
Time               8.14
INSTRUM           spect
PROBHD   5 mm CPPBBO BB
PULPROG      hmbcgpndqf
TD                 4096
SOLVENT            DMSO
NS                   26
DS                   16
SWH            5387.931 Hz
FIDRES         1.315413 Hz
AQ            0.3801088 sec
RG                  203
DW               92.800 usec
DE                10.00 usec
TE                298.0 K
CNST13        4.0000000
D0           0.00000300 sec
D1           1.50000000 sec
D6           0.12500000 sec
D16          0.00020000 sec
IN0          0.00001490 sec

======== CHANNEL f1 ========
SFO1        600.4323659 MHz
NUC1                 1H
P1                11.90 usec
P2                23.80 usec
PLW1        20.51199913 W

======== CHANNEL f2 ========
SFO2        150.9940771 MHz
NUC2                13C
P3                12.00 usec
PLW2        43.00000000 W

====== GRADIENT CHANNEL =====
GPNAM[1]     SMSQ10.100
GPNAM[2]     SMSQ10.100
GPNAM[3]     SMSQ10.100
GPZ1              50.00 %
GPZ2              30.00 %
GPZ3              40.10 %
P16             1000.00 usec

F1 - Acquisition parameters
TD                  256
SFO1           150.9941 MHz
FIDRES       131.082214 Hz
SW              222.241 ppm
FnMODE               QF

F2 - Processing parameters
SI                 1024
SF          600.4300022 MHz
WDW                SINE
SSB      0
LB       0 Hz
GB       0
PC                 1.40

F1 - Processing parameters
SI                 1024
MC2                  QF
SF          150.9782358 MHz
WDW                SINE
SSB      0
LB       0 Hz
GB       0
```
