# Supplementary material for: Discovery of Polyoxypregnane Derivatives From Aspidopterys obcordata With Their Potential Antitumor Activity
Source: Front Chem. 2022 Jan 5;9:799911. doi: 10.3389/fchem.2021.799911 (PMC8766633; doi:10.3389/fchem.2021.799911)
Supplement: Supplementary file 3 [file DataSheet2.ZIP › spectra/e-3/NOE.pdf]

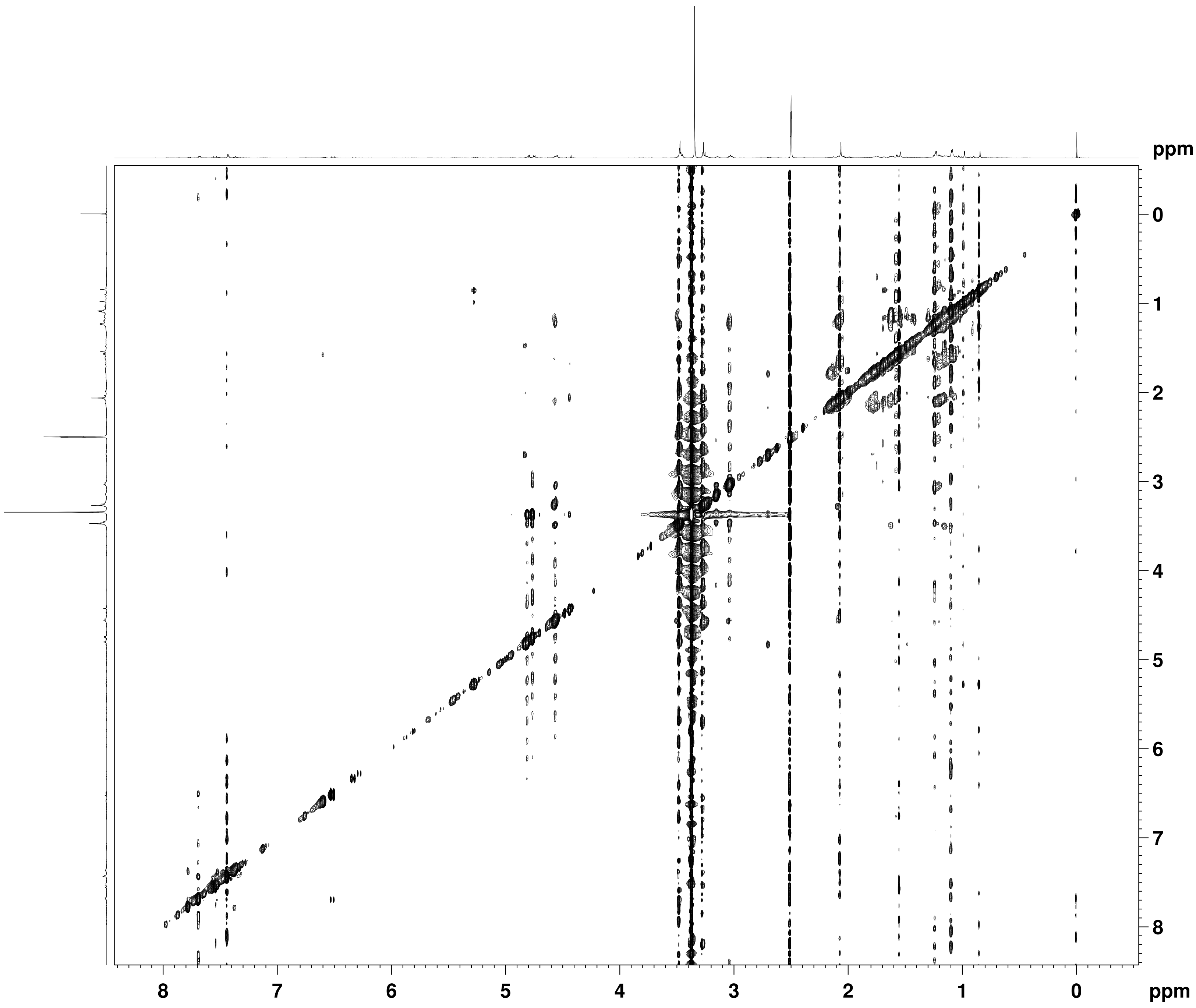


```
Current Data Parameters
NAME        mgx-DCT-e-3
EXPNO                 8
PROCNO                1

F2 - Acquisition Parameters
Date_          20190817
Time               5.42
INSTRUM           spect
PROBHD   5 mm CPPBBO BB
PULPROG     noesygpphpp
TD                 2048
SOLVENT            DMSO
NS                   16
DS                   16
SWH            5387.931 Hz
FIDRES         2.630826 Hz
AQ            0.1900544 sec
RG                  203
DW               92.800 usec
DE                10.00 usec
TE                298.0 K
D0           0.00007765 sec
D1           2.00000000 sec
D8           0.60000002 sec
D11          0.03000000 sec
D12          0.00002000 sec
D16          0.00020000 sec
IN0          0.00018560 sec

======== CHANNEL f1 ========
SFO1        600.4323659 MHz
NUC1                 1H
P1                11.90 usec
P2                23.80 usec
P17             2500.00 usec
PLW1        20.51199913 W
PLW10        4.29689980 W

====== GRADIENT CHANNEL =====
GPNAM[1]     SMSQ10.100
GPZ1              40.00 %
P16             1000.00 usec

F1 - Acquisition parameters
TD                  256
SFO1           600.4324 MHz
FIDRES        21.046606 Hz
SW                8.973 ppm
FnMODE      States-TPPI

F2 - Processing parameters
SI                 1024
SF          600.4299998 MHz
WDW               QSINE
SSB                   2
LB       0 Hz
GB       0
PC                 1.00

F1 - Processing parameters
SI                 1024
MC2         States-TPPI
SF          600.4299996 MHz
WDW               QSINE
SSB                   2
LB       0 Hz
GB       0
```
