# Supplementary material for: Discovery of Polyoxypregnane Derivatives From Aspidopterys obcordata With Their Potential Antitumor Activity
Source: Front Chem. 2022 Jan 5;9:799911. doi: 10.3389/fchem.2021.799911 (PMC8766633; doi:10.3389/fchem.2021.799911)
Supplement: Supplementary file 3 [file DataSheet2.ZIP › spectra/e-3/QC.pdf]

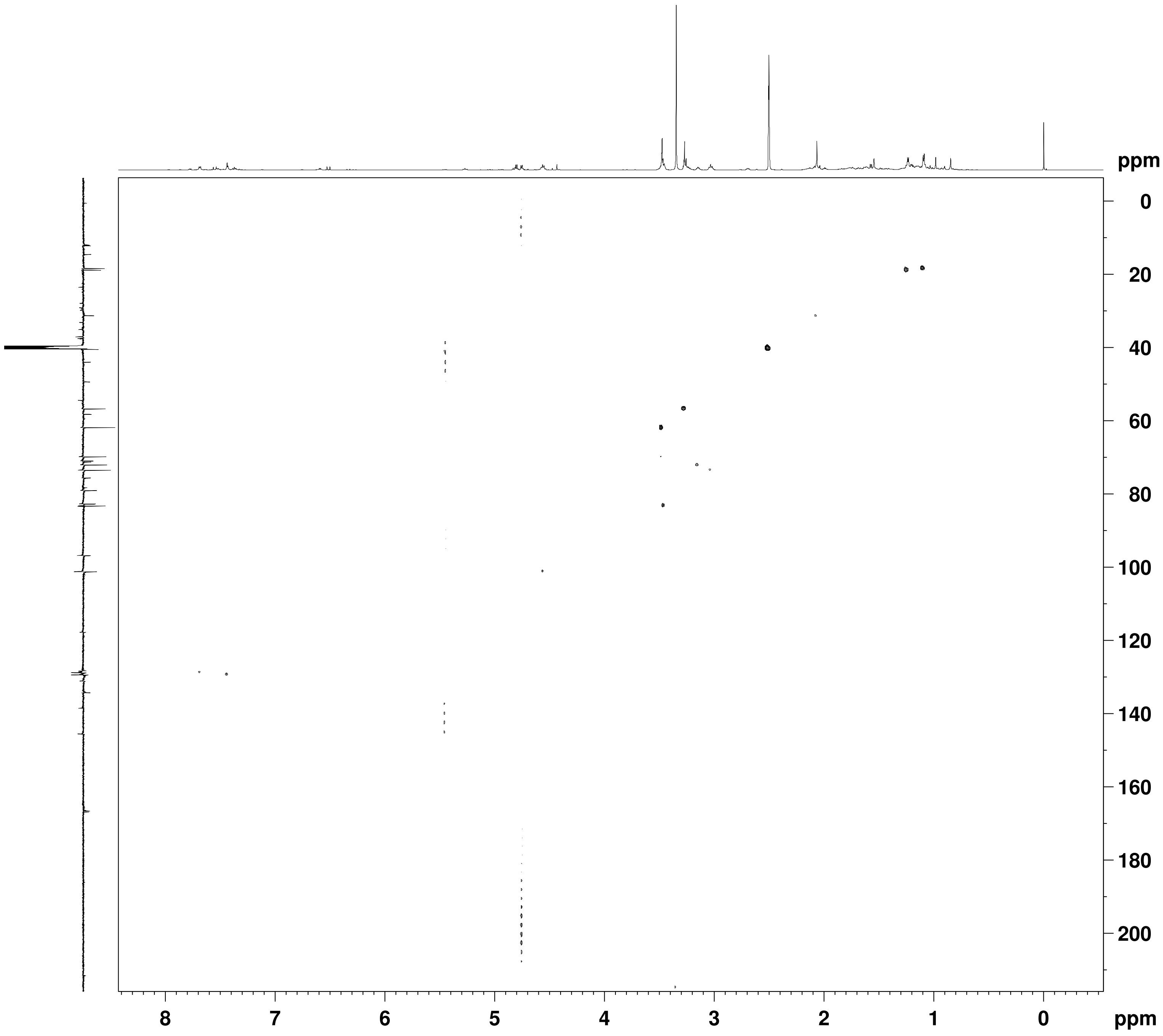


```
Current Data Parameters
NAME        mgx-DCT-e-3
EXPNO                 4
PROCNO                1

F2 - Acquisition Parameters
Date_          20190807
Time               4.35
INSTRUM           spect
PROBHD   5 mm CPPBBO BB
PULPROG  hsqcetgpsisp2.2
TD                 1024
SOLVENT            DMSO
NS                   24
DS                   16
SWH            5387.931 Hz
FIDRES         5.261652 Hz
AQ            0.0950272 sec
RG                 25.4
DW               92.800 usec
DE                10.00 usec
TE                298.0 K
CNST2       145.0000000
CNST17       -0.5000000
D0           0.00000300 sec
D1           2.00000000 sec
D4           0.00172414 sec
D11          0.03000000 sec
D16          0.00020000 sec
D24          0.00089000 sec
IN0          0.00001490 sec

======== CHANNEL f1 ========
SFO1       600.4323659 MHz
NUC1                 1H
P1                11.90 usec
P2                23.80 usec
P28      0 usec
PLW1        20.51199913 W

======== CHANNEL f2 ========
SFO2       150.9940780 MHz
NUC2                13C
CPDPRG[2           garp
P3                12.00 usec
P14              500.00 usec
P24             2000.00 usec
PCPD2             75.00 usec
PLW0     0 W
PLW2        43.00000000 W
PLW12        1.10080004 W
SPNAM[3] Crp60,0.5,20.1
SPOAL3            0.500
SPOFFS3  0 Hz
SPW3         9.46070004 W
SPNAM[7]    Crp60comp.4
SPOAL7            0.500
SPOFFS7  0 Hz
SPW7         9.46070004 W

====== GRADIENT CHANNEL =====
GPNAM[1]     SMSQ10.100
GPNAM[2]     SMSQ10.100
GPNAM[3]     SMSQ10.100
GPNAM[4]     SMSQ10.100
GPZ1              80.00 %
GPZ2              20.10 %
GPZ3              11.00 %
GPZ4              -5.00 %
P16             1000.00 usec
P19              600.00 usec

F1 - Acquisition parameters
TD                  256
SFO1           150.9941 MHz
FIDRES       131.082214 Hz
SW              222.241 ppm
FnMODE    Echo-Antiecho

F2 - Processing parameters
SI                 1024
SF          600.4299996 MHz
WDW               QSINE
SSB                   2
LB       0 Hz
GB       0
PC                 1.40

F1 - Processing parameters
SI                 1024
MC2        echo-antiecho
SF          150.9782618 MHz
WDW               QSINE
SSB                   2
LB       0 Hz
GB       0
```
