# Supplementary material for: Discovery of Polyoxypregnane Derivatives From Aspidopterys obcordata With Their Potential Antitumor Activity
Source: Front Chem. 2022 Jan 5;9:799911. doi: 10.3389/fchem.2021.799911 (PMC8766633; doi:10.3389/fchem.2021.799911)
Supplement: Supplementary file 3 [file DataSheet2.ZIP › spectra/e-5-1/BC.pdf]

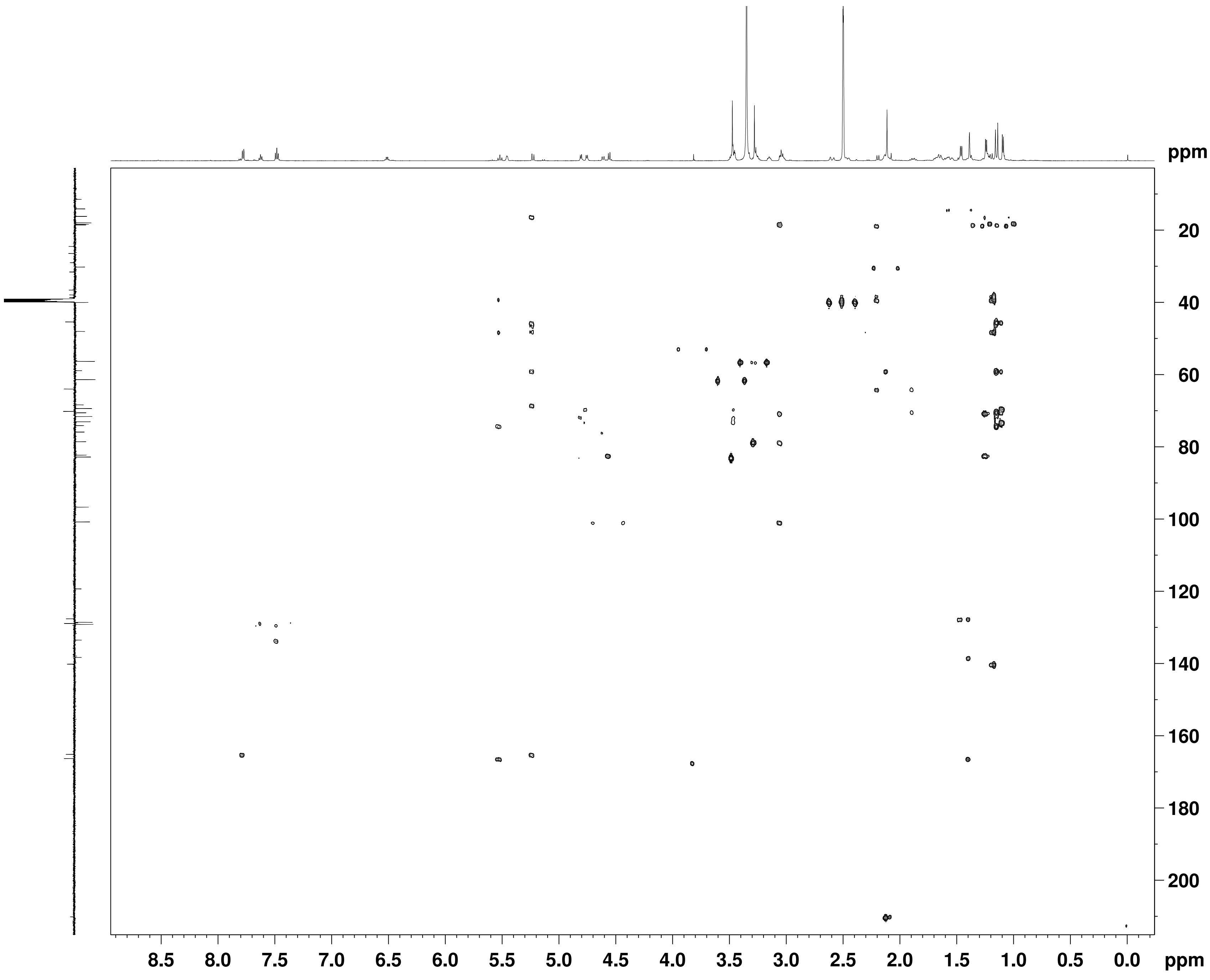


```
Current Data Parameters
NAME      mgx-DCT-e-5-1
EXPNO                 5
PROCNO                1

F2 - Acquisition Parameter
Date_          20190825
Time               8.52
INSTRUM           spect
PROBHD   5 mm CPPBBO BB
PULPROG      hmbcgpndqf
TD                 4096
SOLVENT            DMSO
NS                   26
DS                   16
SWH            5514.706 Hz
FIDRES         1.346364 Hz
AQ            0.3713707 se
RG                  203
DW               90.667 us
DE                10.00 us
TE                298.0 K
CNST13        4.0000000
D0           0.00000300 se
D1           1.50000000 se
D6           0.12500000 se
D16          0.00020000 se
IN0          0.00001560 se

======== CHANNEL f1 ======
SFO1        600.4326127 MH
NUC1                 1H
P1                11.90 us
P2                23.80 us
PLW1        20.51199913 W

======== CHANNEL f2 ======
SFO2        150.9946996 MH
NUC2                13C
P3                12.00 us
PLW2        43.00000000 W

====== GRADIENT CHANNEL ==
GPNAM[1]     SMSQ10.100
GPNAM[2]     SMSQ10.100
GPNAM[3]     SMSQ10.100
GPZ1              50.00 %
GPZ2              30.00 %
GPZ3              40.10 %
P16             1000.00 us

F1 - Acquisition parameter
TD                  256
SFO1           150.9947 MH
FIDRES       125.200317 Hz
SW              212.268 pp
FnMODE               QF

F2 - Processing parameters
SI                 1024
SF          600.4299998 MH
WDW                SINE
SSB      0
LB       0 Hz
GB       0
PC                 1.40

F1 - Processing parameters
SI                 1024
MC2                  QF
SF          150.9782498 MH
WDW                SINE
SSB      0
LB       0 Hz
GB       0
```
