# Supplementary material for: Discovery of Polyoxypregnane Derivatives From Aspidopterys obcordata With Their Potential Antitumor Activity
Source: Front Chem. 2022 Jan 5;9:799911. doi: 10.3389/fchem.2021.799911 (PMC8766633; doi:10.3389/fchem.2021.799911)
Supplement: Supplementary file 3 [file DataSheet2.ZIP › spectra/e-5-1/C.pdf]

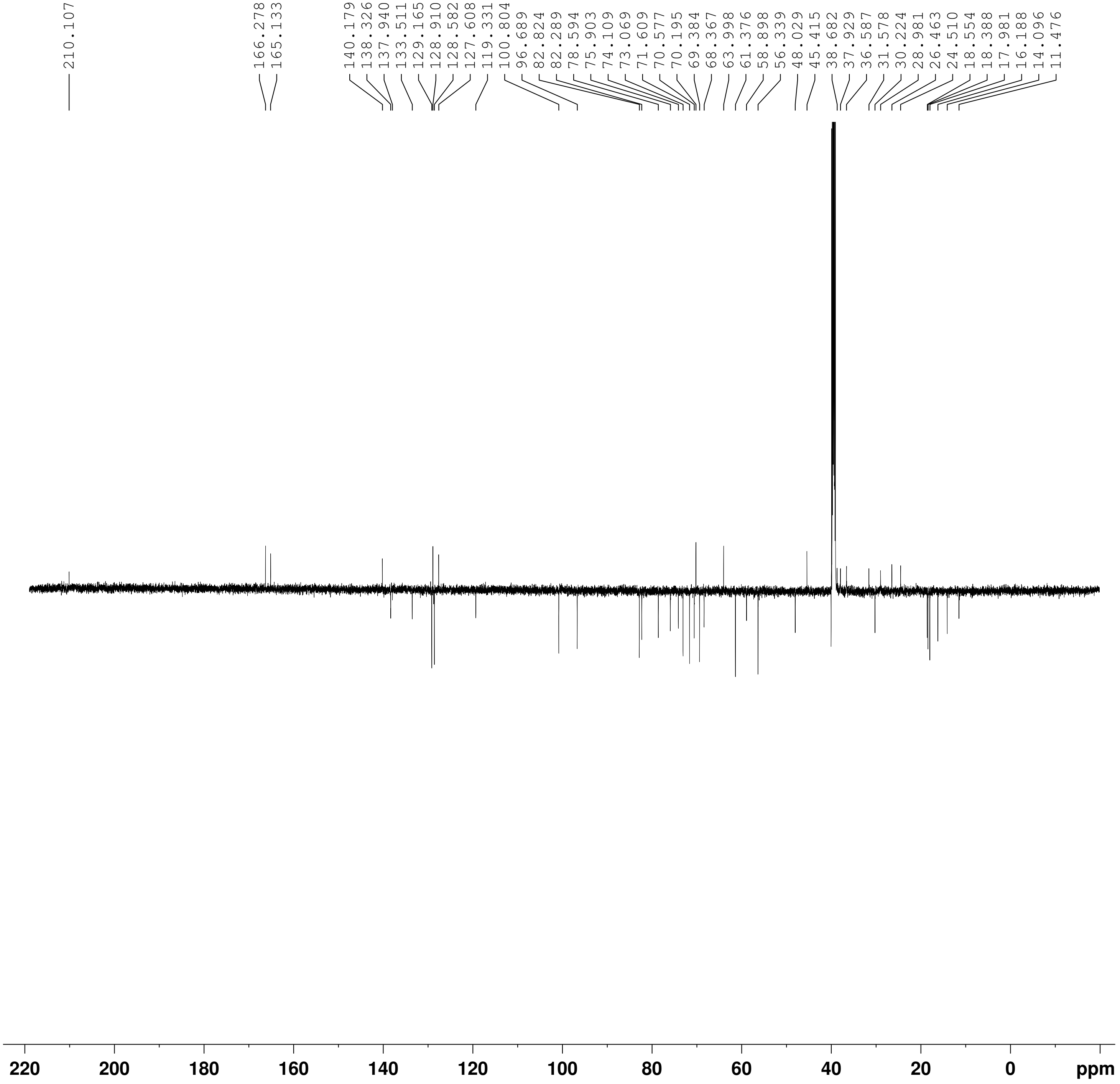

Current Data Parameters

NAME	mgx-DCT-e-5-1
EXPNO	2
PROCNO	1

F2 - Acquisition Paramet

Date_	20190822
Time	20.06
INSTRUM	spect
PROBHD	5 mm CPPBBO BB
PULPROG	jmod
TD	65536
SOLVENT	DMSO
NS	1536
DS	4
SWH	36057.691
FIDRES	0.550197
AQ	0.9087659
RG	203
DW	13.867
DE	18.00
TE	298.0
CNST2	145.0000000
CNST11	1.0000000
D1	2.00000000
D20	0.00689655
TD0	6

======== CHANNEL f1 ====

SFO1	150.9933414
NUC1	13C
P1	12.00
P2	24.00
PLW1	43.00000000

======== CHANNEL f2 ====

SFO2	600.4324017
NUC2	1H
CPDPRG[2	waltz16
PCPD2	80.00
PLW2	20.51199913
PLW12	0.45386001

F2 - Processing paramete

SI		32768
SF		150.9783140
WDW		EM
SSB	0	
LB		1.00
GB	0	
PC		1.40
