# Supplementary material for: Discovery of Polyoxypregnane Derivatives From Aspidopterys obcordata With Their Potential Antitumor Activity
Source: Front Chem. 2022 Jan 5;9:799911. doi: 10.3389/fchem.2021.799911 (PMC8766633; doi:10.3389/fchem.2021.799911)
Supplement: Supplementary file 3 [file DataSheet2.ZIP › spectra/e-5-1/H.pdf]

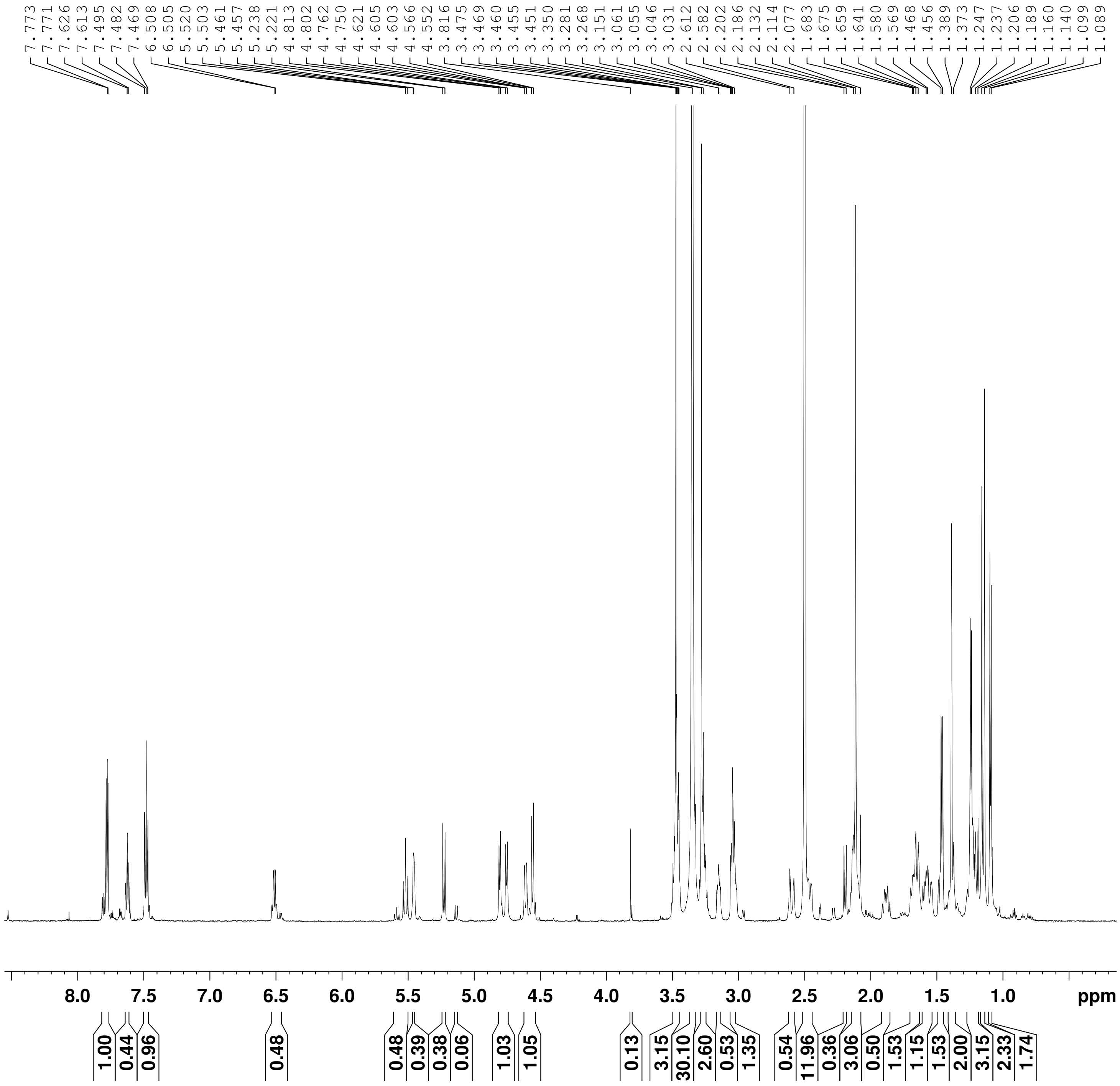


```
Current Data Parameters
NAME        mgx-DCT-e-5-1
EXPNO                   1
PROCNO                  1

F2 - Acquisition Paramet
Date_            20190820
Time                15.42
INSTRUM             spect
PROBHD   5 mm CPPBBO BB
PULPROG              zg30
TD                  65536
SOLVENT              DMSO
NS                     16
DS                      2
SWH             12019.230
FIDRES           0.183399
AQ              2.7262976
RG                   90.5
DW                 41.600
DE                  10.00
TE                  298.0
D1             1.00000000
TD0                     1

======== CHANNEL f1 ====
SFO1         600.4337079
NUC1                  1H
P1                  11.90
PLW1         20.51199913

F2 - Processing paramete
SI                  65536
SF           600.4300086
WDW                    EM
SSB      0
LB                   0.30
GB       0
PC                   1.00
```
