# Supplementary material for: Discovery of Polyoxypregnane Derivatives From Aspidopterys obcordata With Their Potential Antitumor Activity
Source: Front Chem. 2022 Jan 5;9:799911. doi: 10.3389/fchem.2021.799911 (PMC8766633; doi:10.3389/fchem.2021.799911)
Supplement: Supplementary file 3 [file DataSheet2.ZIP › spectra/e-5-1/QC.pdf]

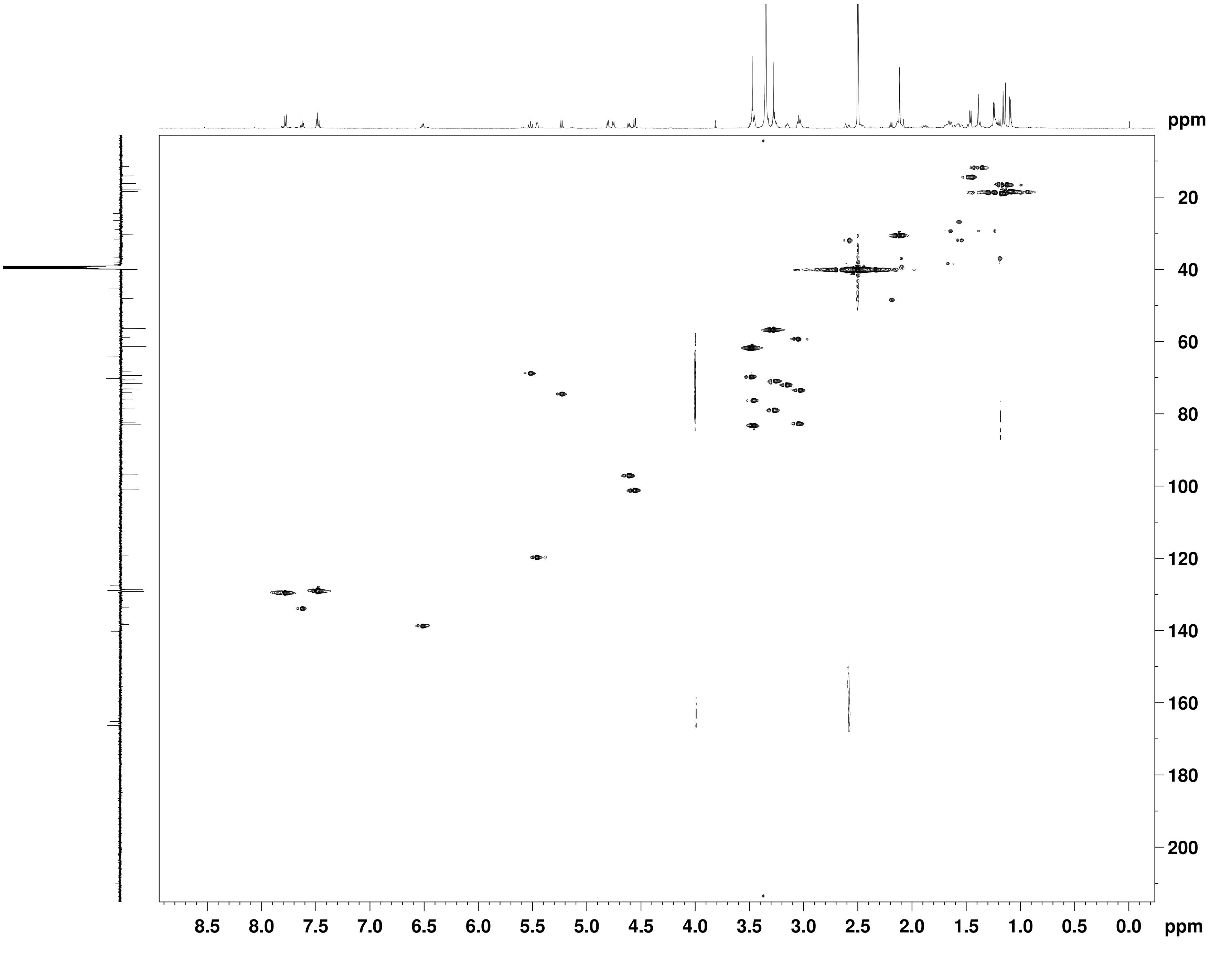

Current Data Parameters
NAME mgx-DCT-e-5-1
EXPNO 4
PROCNO 1

F2 - Acquisition Parameter
Date_ 20190825
Time 5.14
INSTRUM spect
PROBHD 5 mm CPPBBO BB
PULPROG hsqcetgpsisp2.2
TD 1024
SOLVENT DMSO
NS 24
DS 16
SWH 5514.706 Hz
FIDRES 5.385455 Hz
AQ 0.0928427 se
RG 203
DW 90.667 us
DE 10.00 us
TE 298.0 K
CNST2 145.0000000
CNST17 -0.5000000
D0 0.00000300 se
D1 2.00000000 se
D4 0.00172414 se
D11 0.03000000 se
D16 0.00020000 se
D24 0.00089000 se
IN0 0.00001560 se

======== CHANNEL f1 ======
SFO1 600.4326127 MH
NUC1 1H
P1 11.90 us
P2 23.80 us
P28 0 usec
PLW1 20.51199913 W

======== CHANNEL f2 ======
SFO2 150.9946996 MH
NUC2 13C
CPDPRG[2 garp
P3 12.00 us
P14 500.00 us
P24 2000.00 us
PCPD2 75.00 us
PLW0 0 W
PLW2 43.00000000 W
PLW12 1.10080004 W
SPNAM[3] Crp60,0.5,20.1
SPOAL3 0.500
SPOFFS3 0 Hz
SPW3 9.46070004 W
SPNAM[7] Crp60comp.4
SPOAL7 0.500
SPOFFS7 0 Hz
SPW7 9.46070004 W

====== GRADIENT CHANNEL ==
GPNAM[1] SMSQ10.100
GPNAM[2] SMSQ10.100
GPNAM[3] SMSQ10.100
GPNAM[4] SMSQ10.100
GPZ1 80.00 %
GPZ2 20.10 %
GPZ3 11.00 %
GPZ4 -5.00 %
P16 1000.00 us
P19 600.00 us

F1 - Acquisition parameter
TD 256
SFO1 150.9947 MH
FIDRES 125.200317 Hz
SW 212.268 pp
FnMODE Echo-Antiecho

F2 - Processing parameters
SI 1024
SF 600.4299997 MH
WDW QSINE
SSB 2
LB 0 Hz
GB 0
PC 1.40

F1 - Processing parameters
SI 1024
MC2 echo-antiecho
SF 150.9782419 MH
WDW QSINE
SSB 2
LB 0 Hz
GB 0
