# Supplementary material for: Discovery of Polyoxypregnane Derivatives From Aspidopterys obcordata With Their Potential Antitumor Activity
Source: Front Chem. 2022 Jan 5;9:799911. doi: 10.3389/fchem.2021.799911 (PMC8766633; doi:10.3389/fchem.2021.799911)
Supplement: Supplementary file 3 [file DataSheet2.ZIP › spectra/e-7/H.pdf]

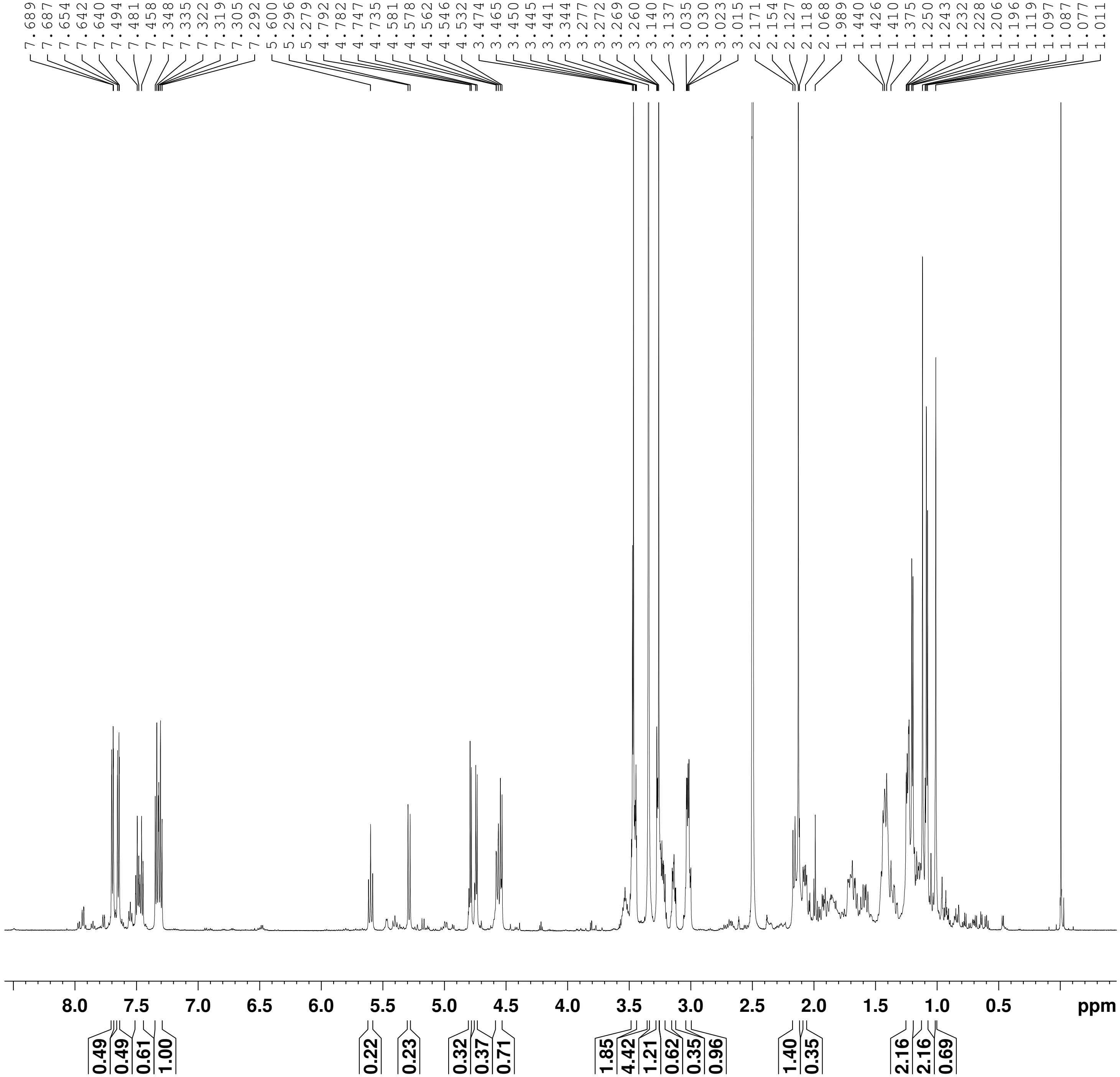

Current Data Parameters
NAME mgx-DCT-e-7
EXPNO 1
PROCNO 1

F2 - Acquisition Paramet
Date_ 20190702
Time 20.41
INSTRUM spect
PROBHD 5 mm CPPBBO BB
PULPROG zg30
TD 65536
SOLVENT DMSO
NS 16
DS 2
SWH 12019.230
FIDRES 0.183399
AQ 2.7262976
RG 90.5
DW 41.600
DE 10.00
TE 298.1
D1 1.00000000
TD0 1

======== CHANNEL f1 ====
SFO1 600.4337079
NUC1 1H
P1 11.90
PLW1 20.51199913

F2 - Processing paramete
SI 65536
SF 600.4300094
WDW EM
SSB 0
LB 0.30
GB 0
PC 1.00
