# Supplementary material for: Discovery of Polyoxypregnane Derivatives From Aspidopterys obcordata With Their Potential Antitumor Activity
Source: Front Chem. 2022 Jan 5;9:799911. doi: 10.3389/fchem.2021.799911 (PMC8766633; doi:10.3389/fchem.2021.799911)
Supplement: Supplementary file 3 [file DataSheet2.ZIP › spectra/h-1/BC.pdf]

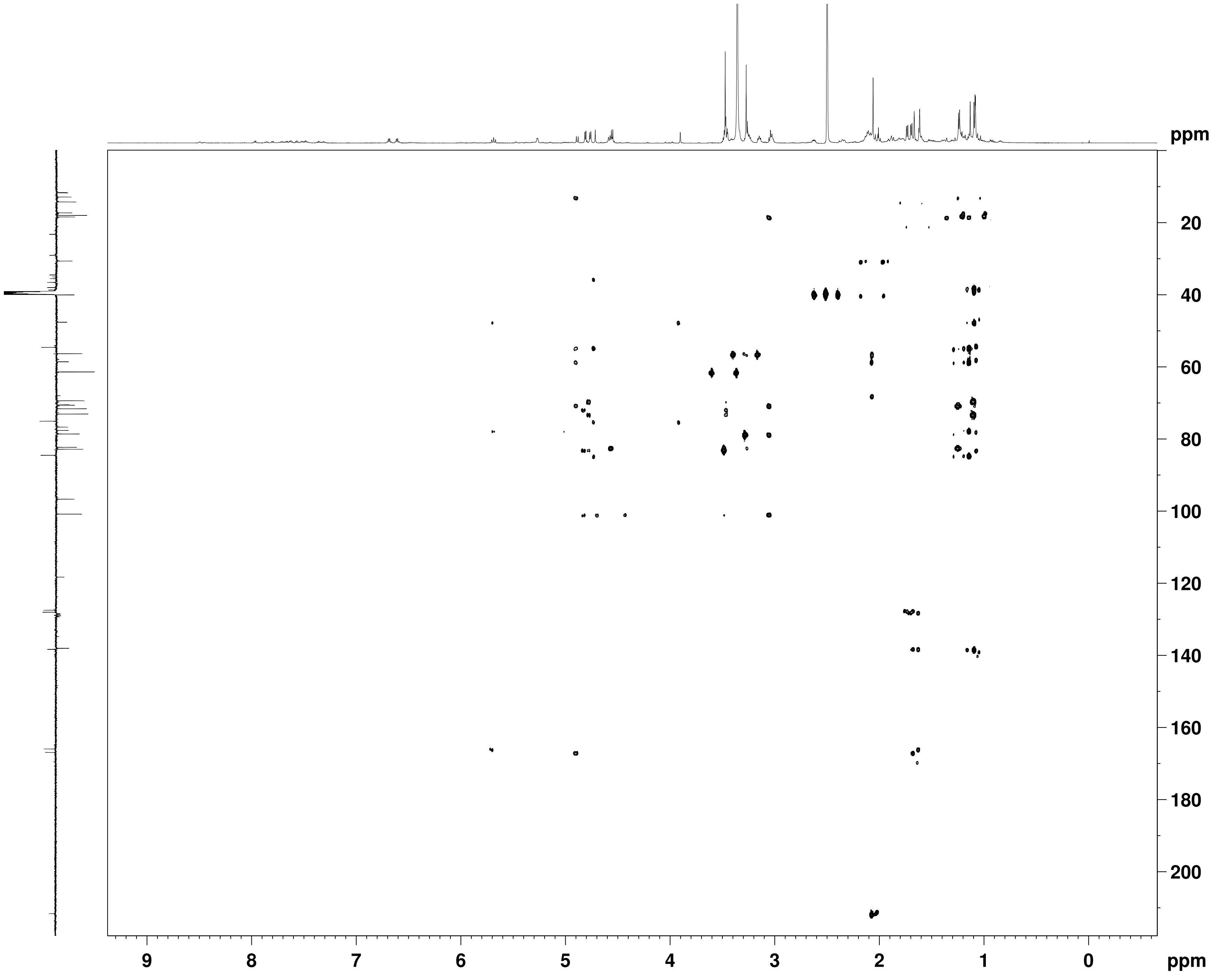

Current Data Parameters
NAME mgx-DCT-h-1
EXPNO 6
PROCNO 1

F2 - Acquisition Paramete
Date_ 20191014
Time 6.03
INSTRUM spect
PROBHD 5 mm CPPBBO BB
PULPROG hmbcgpndqf
TD 4096
SOLVENT DMSO
NS 32
DS 16
SWH 6024.096 H
FIDRES 1.470727 H
AQ 0.3399680 s
RG 203
DW 83.000 u
DE 10.00 u
TE 295.8 K
CNST13 4.0000000
D0 0.00000300 s
D1 1.50000000 s
D6 0.12500000 s
D16 0.00020000 s
IN0 0.00001520 s

======== CHANNEL f1 =====
SFO1 600.4326176 M
NUC1 1H
P1 11.90 u
P2 23.80 u
PLW1 20.51199913 W

======== CHANNEL f2 =====
SFO2 150.9946829 M
NUC2 13C
P3 12.00 u
PLW2 43.00000000 W

====== GRADIENT CHANNEL =
GPNAM[1] SMSQ10.100
GPNAM[2] SMSQ10.100
GPNAM[3] SMSQ10.100
GPZ1 50.00 %
GPZ2 30.00 %
GPZ3 40.10 %
P16 1000.00 u

F1 - Acquisition paramete
TD 256
SFO1 150.9947 M
FIDRES 128.495071 H
SW 217.854 p
FnMODE QF

F2 - Processing parameter
SI 1024
SF 600.4299997 M
WDW SINE
SSB 0
LB 0 Hz
GB 0
PC 1.40

F1 - Processing parameter
SI 1024
MC2 QF
SF 150.9782512 M
WDW SINE
SSB 0
LB 0 Hz
GB 0
