# Supplementary material for: Discovery of Polyoxypregnane Derivatives From Aspidopterys obcordata With Their Potential Antitumor Activity
Source: Front Chem. 2022 Jan 5;9:799911. doi: 10.3389/fchem.2021.799911 (PMC8766633; doi:10.3389/fchem.2021.799911)
Supplement: Supplementary file 3 [file DataSheet2.ZIP › spectra/h-1/C.pdf]

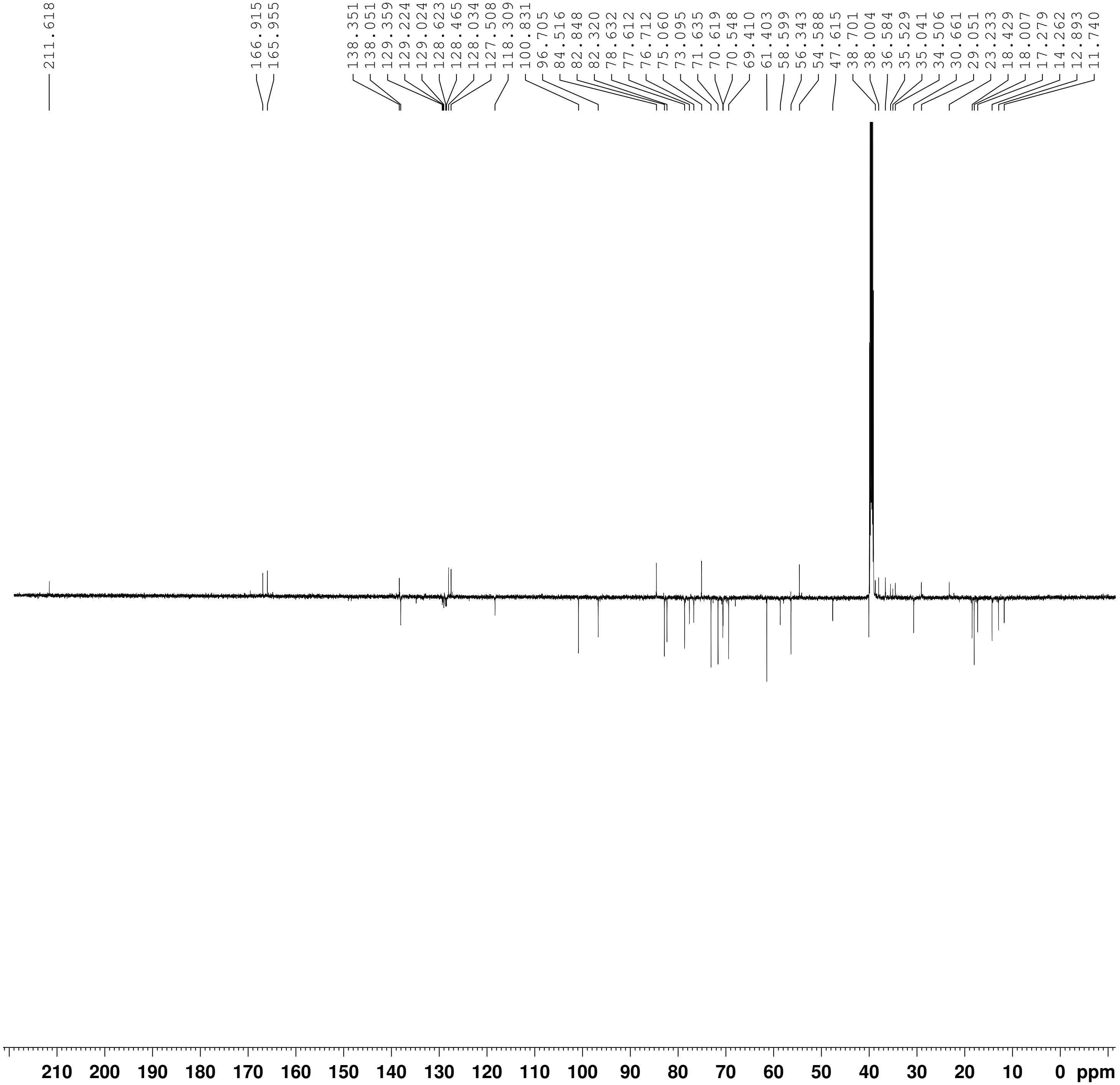

Current Data Parameters
NAME mgx-DCT-h-1
EXPNO 1
PROCNO 1

F2 - Acquisition Paramet
Date_ 20191001
Time 11.31
INSTRUM spect
PROBHD 5 mm CPPBBO BB
PULPROG jmod
TD 65536
SOLVENT DMSO
NS 5120
DS 4
SWH 36057.691
FIDRES 0.550197
AQ 0.9087659
RG 203
DW 13.867
DE 18.00
TE 298.0
CNST2 145.0000000
CNST11 1.0000000
D1 2.00000000
D20 0.00689655
TD0 20

======== CHANNEL f1 ====
SFO1 150.9933414
NUC1 13C
P1 12.00
P2 24.00
PLW1 43.00000000

======== CHANNEL f2 ====
SFO2 600.4324017
NUC2 1H
CPDPRG[2 waltz16
PCPD2 80.00
PLW2 20.51199913
PLW12 0.45386001

F2 - Processing paramete
SI 32768
SF 150.9783098
WDW EM
SSB 0
LB 1.00
GB 0
PC 1.40
