# Supplementary material for: Discovery of Polyoxypregnane Derivatives From Aspidopterys obcordata With Their Potential Antitumor Activity
Source: Front Chem. 2022 Jan 5;9:799911. doi: 10.3389/fchem.2021.799911 (PMC8766633; doi:10.3389/fchem.2021.799911)
Supplement: Supplementary file 3 [file DataSheet2.ZIP › spectra/h-1/H.pdf]

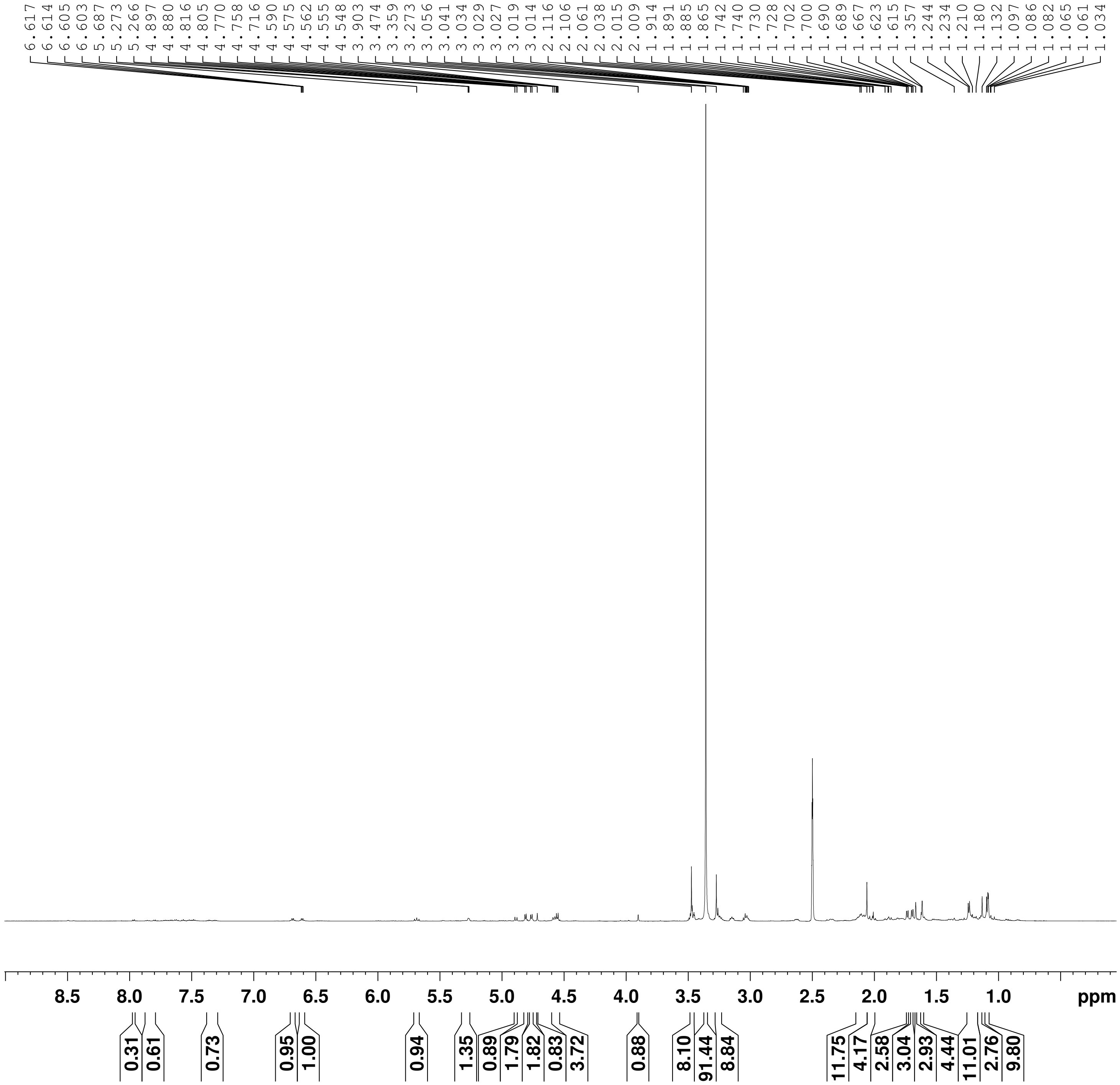


```
Current Data Parameters
NAME        mgx-DCT-h-1
EXPNO                 3
PROCNO                1

F2 - Acquisition Paramet
Date_          20191013
Time              22.39
INSTRUM           spect
PROBHD   5 mm CPPBBO BB
PULPROG            zg30
TD                65536
SOLVENT            DMSO
NS                   16
DS                    2
SWH           12019.230
FIDRES         0.183399
AQ            2.7262976
RG                 90.5
DW               41.600
DE                10.00
TE                295.5
D1           1.00000000
TD0                   1

======== CHANNEL f1 ====
SFO1        600.4337079
NUC1                 1H
P1                11.90
PLW1        20.51199913

F2 - Processing paramete
SI                65536
SF          600.4300088
WDW                  EM
SSB      0
LB                 0.30
GB       0
PC                 1.00
```
